# Food environments and dietary intakes among adults: does the type of spatial exposure measurement matter? A systematic review

**DOI:** 10.1186/s12942-018-0139-7

**Published:** 2018-06-09

**Authors:** Alexia Bivoltsis, Eleanor Cervigni, Gina Trapp, Matthew Knuiman, Paula Hooper, Gina Leslie Ambrosini

**Affiliations:** 1 School of Population and Global Health, The University of Western Australia, M451, 35 Stirling Highway, Crawley, Perth, WA 6009 Australia; 2 School of Human Sciences, The University of Western Australia, 35 Stirling Highway, Crawley, WA 6009 Australia; 3 Telethon Kids Institute, The University of Western Australia, PO Box 855, West Perth, WA 6872 Australia; 4 School of Agriculture and Environment and the School of Human Sciences, The University of Western Australia, 35 Stirling Highway, Crawley, WA 6009 Australia

**Keywords:** Community food environment, Diet, Geographic Information Systems, Spatial, Access

## Abstract

**Background:**

The relationships between food environments and dietary intake have been assessed via a range of methodologically diverse measures of spatial exposure to food outlets, resulting in a largely inconclusive body of evidence, limiting informed policy intervention.

**Objective:**

This systematic review aims to evaluate the influence of methodological choice on study outcomes by examining the within-study effect of availability (e.g., counts) versus accessibility (e.g., proximity) spatial exposure measures on associations with diet.

**Methods:**

(PROSPERO registration: CRD42018085250). PubMed, Web of Science, Scopus and ScienceDirect databases were searched for empirical studies from 1980 to 2017, in the English language, involving adults and reporting on the statistical association between a dietary outcome and spatial exposure measures of both availability *and* accessibility. Studies were appraised using an eight-point quality criteria with a narrative synthesis of results.

**Results:**

A total of 205 associations and 44 relationships (i.e., multiple measures of spatial exposure relating to a particular food outlet type and dietary outcome) were extracted from 14 eligible articles. Comparative measures were dominated by counts (availability) and proximity (accessibility). Few studies compared more complex measures and all counts were derived from place-based measures of exposure. Sixteen of the 44 relationships had a significant effect involving an availability measure whilst only 8 had a significant effect from an accessibility measure. The largest effect sizes in relationships were mostly for availability measures. After stratification by scale, availability measure had the greatest effect size in 139 of the 176 pairwise comparisons. Of the 33% (68/205) of associations that reached significance, 53/68 (78%) were from availability measures. There was no relationship between study quality and reported study outcomes.

**Conclusions:**

The limited evidence suggests that availability measures may produce significant and greater effect sizes than accessibility measures. However, both availability and accessibility measures may be important concepts of spatial exposure depending on the food outlet type and dietary outcome examined. More studies reporting on multi-method effects are required to differentiate findings by the type of spatial exposure assessment and build an evidence base regarding the appropriateness and robustness of measures under different circumstances.

**Electronic supplementary material:**

The online version of this article (10.1186/s12942-018-0139-7) contains supplementary material, which is available to authorized users.

## Background

Dietary risk factors are the leading cause of global illness, disability and death, largely due to cardiovascular disease, cancer and diabetes [1]. The community food environment (CFE), defined as the location, type and number of food outlets [2], is recognised as an important factor influencing dietary choices. As such, a number of studies have investigated the link between spatial exposure to food outlet types and dietary outcomes using geographic information systems (GIS) methods [3–6]. The resulting body of evidence is based on a methodologically diverse range of spatial exposure measures and mixed results, with the majority reporting null findings [3]. It is still unclear what influence the type of spatial exposure measure has on reported associations with diet. A better understanding of the methodological influence on study outcomes is required for effective application of CFE planning and policy interventions aimed at improving population dietary choices.

At present, the two main measures of spatial exposure frequently applied within the CFE-diet literature are density and proximity [5, 7, 8]. For the purpose of this study, we describe density measures as belonging to the spatial dimension of “availability” and are based on the CFE within a defined area (neighbourhood) in terms of the presence, ratio, variety, count, relative density or diversity of outlets. In contrast, proximity measures are described as “accessibility” measures based on the distance between a reference point and the surrounding CFE. Proximity measures are usually expressed as road network distances, straight line distances, travel times or spatial interaction models including gravity models that quantify the distance decay relationship between two locations where utilisation declines with increasing distance from a point of reference. Many variations of availability and accessibility measures have been employed that are determined using different methods of calculation (e.g., accessibility measures determined using straight line Euclidean distances or road network distances and availability measures determined using probability density functions such as kernel density estimations or simple counts within defined pre-buffers) [9]. Heterogeneity of measures is continuously cited as a challenge when interpreting outcomes across multiple studies [10–13] and contributes to the current conflicting CFE-diet evidence base.

Studies examining disparities in the CFE using measures of availability and accessibility have begun to explore the sensitivity of these measures [8, 14–22]. Accessibility measures have been shown to be robust to variations in the method of calculation (e.g., Euclidean versus road network distances) [8, 14, 15]. For example, correlation coefficients calculated for road network and Euclidean distances to supermarkets (r = 0.97) and convenience stores (r = 0.96) suggest a high degree of similarity between these measures [16]. By comparison, availability measures are more sensitive to variations in the method of calculation [15]. For example, road network buffers tend to produce smaller neighbourhood sizes than Euclidean buffers, potentially altering measures of availability such as counts or relative densities [14, 17, 18]. However, there is less consensus regarding the similarity between availability and accessibility measures. Some research suggests that measures such as counts and proximity produce different results [19–21] and differing associations with neighbourhood socio-economic disadvantage [22]. However, others conclude that relative density and/or count and proximity measures are largely comparable [14, 15]. Availability and accessibility measures belong to distinctly different theoretical concepts of exposure [7], thus are expected to produce distinct measures. Yet the similarities and differences between these measures are largely unknown.

Individuals may interact with surrounding food outlets in different ways. To address this, studies are beginning to employ more than one spatial measure to account for multiple concepts of exposure. Yet few have assessed the effect that different spatial exposure measures have on the relationship with diet [23]. The type of spatial measure could potentially under- or over-estimate the degree of exposure and influence reported associations with diet. For example, the association between supermarkets and individual dietary intake has been demonstrated to vary depending on the type of spatial exposure measurement [23]. However, it is unclear whether these findings apply to other food outlet types (e.g., fast food outlets) and a range of dietary outcomes (e.g., fast food intake or diet quality). Overall, little is known about how the CFE-diet relationship differs depending on the measure of spatial exposure for certain food outlet types and dietary outcomes.

As governments and policy-makers are increasingly looking for interventions to address the current global obesity epidemic, there is demand for a greater understanding of the CFE-diet relationship [24]. Spatial information regarding the CFE-diet relationship can provide specific evidence to both planners and policy-makers to inform environment level interventions with the potential for widespread effects on population dietary intakes (e.g., modifying the location, type and number of food outlets). This reinforces the need for a timely review of studies that have utilised more than one measure of spatial exposure, to assess how methodological variations influence the CFE-diet relationship.

Recent reviews of the CFE-diet relationship have not assessed study quality or distinguished between the effects of spatial exposure measure on study outcomes [3, 4, 11, 25, 26]. Furthermore, previous methodological reviews have mostly focused on weight related outcomes rather than diet [27], looked at variations in statistical techniques and not exposure methodology [28], been limited to descriptive evaluations of spatial exposure measures (i.e., provided a summary and definition of the spatial exposure measures employed within the literature without discussion of their relative effects on study outcomes) [5, 29], or compared results by type of spatial exposure measurement across studies and not examined within-study effects [5, 30].

This systematic review aimed to (1) identify and characterise studies that have employed measures of both availability and accessibility to examine the CFE-diet relationship, and (2) evaluate current within-study evidence to determine what effect the choice of spatial exposure measure (availability versus accessibility) had on the CFE-diet relationship. Specifically, we investigated the following research question: *Does the choice of within*-*study spatial exposure measure (availability versus accessibility) influence associations between the community food environment and diet?* Findings will contribute to a greater understanding of the similarities and differences among spatial exposure measures, help guide future research decisions regarding the choice of spatial exposure measure, and contribute towards establishing indicators of food outlet exposure linked with dietary outcomes. Finally, we provide recommendations to improve future studies involving the use of spatial exposure measurements.

## Methods

The systematic steps outlined in the PRISMA guidelines were used in this review [31]. See also PRISMA checklist (Additional file 1). The full review protocol and PROSPERO registration details are available in the public domain (PROSPERO registration number: CRD42018085250). https://www.crd.york.ac.uk/PROSPERO/display_record.php?RecordID=85250.

### Search strategy

Citations were retrieved through a series of searches in the PubMed, Web of Science, Scopus and ScienceDirect databases. Searches were conducted using combinations of keywords within the title and abstract: (density OR proximity OR GIS OR geographic OR spatial OR exposure OR access* OR location) AND (“food environment” OR neigh* OR “built environment” OR retail OR outlet* OR store OR “nutrition environment” OR foodscape OR supermarket OR shop) AND (diet* OR intake OR fruit OR vegetable OR food OR consumption OR purchase OR health* OR nutrition) AND NOT (child* OR school* OR adolescent*). Keywords were initially obtained from relevant reviews and articles and guided by Medical Subject Heading (MeSH) terms relevant to each concept (see Additional file 2). Final keyword combinations were refined through a series of iterative searches. Further searches were conducted using combinations of MeSH terms: (Environment Design”[Mesh] AND “Spatial Analysis”[Mesh]), (Food Analysis”[Mesh] AND “Food Supply”[Mesh]). Database searches were supplemented with cited reference searching using all included articles in the Scopus database and additional citations retrieved manually from relevant reviews [3–6, 10–13, 25, 26, 32–36] and the reference lists of included articles. The search included articles published from 1980 up to December 2017. A detailed outline of the search strategy, keywords and restrictions applied is available in Additional file 2.

### Inclusion and exclusion criteria

The inclusion criteria were: original journal articles of published, peer-reviewed, empirical studies; in the English language; involving adult (≥ 18 years) human participants; using observational or experimental design; measuring spatial exposure to the CFE surrounding residences or within residential areas; involving a dietary outcome (purchase or intake); and reporting on the statistical associations between spatial exposure measures of both availability *and* accessibility. We included studies that derived neighbourhood size using all known methods (e.g., self-defined, network based, and via the use of global positioning system (GPS) technologies). Studies were excluded if: they examined the CFE surrounding work places or schools; they involved mobile food outlets and/or vending machines; or comparative spatial exposure measures were not of the same food outlet type or dietary outcome.

We chose the residential environment because it is the most frequently studied. Similarly, the review was limited to adults due to the increased availability of published studies and because the underlying relationship between the CFE and diet may differ for children and adolescents. Although it is acknowledged that other aspects of the food environment (e.g., price, within store food availability, store preference) may influence diet, these were beyond the scope of this review.

### Study selection

The titles of all retrieved citations were initially screened by one reviewer (author one) and excluded if they were outside the study scope or duplicates. The remaining citations were imported into Abstrackr [37] and abstracts examined for eligibility and inclusion by two independent reviewers (author one and two). The full text articles of included abstracts were retrieved and further screened by the same two reviewers to determine final eligibility. Discrepancies between reviewers surrounding the eligibility of a particular study were resolved by further evaluation and consensus.

### Data extraction

Data were independently extracted (author one) from each included article relating to study design, study population, location, sample size and response rate, exposure measurement details (measurement type, geographic scale and level of data aggregation, food outlet data sources, year of food outlet data collection, food outlet type and method of classification), dietary outcomes, dietary assessment method, statistical analyses, adjustment variables, and study results for each exposure–outcome relationship examined (i.e., for each published association of food outlet type, spatial exposure measure and dietary outcome). All extracted associations included a measure of the effect estimate, and where possible, the precision [i.e., 95% confidence interval (CI) or standard error (SE)]. In addition, the *p* value or significance level was also extracted (i.e., having a significant association in the expected, unexpected direction or null (non-significant) findings). Results were extracted from final adjusted models, or best fit with a significance level of ≤ 0.05. When articles published the standard deviation (SD) of exposure variables, this information was also extracted and used to calculate a standardised effect size.

### Data analyses

Extracted odds ratios (OR) were converted to beta (β) regression coefficients (i.e., β = ln (OR)), for ease of interpretation across studies and, where possible, standardised (i.e., standardised effect estimate = β*SD). Standardisation of β regression coefficients enabled meaningful interpretation of within-study effects when spatial exposure measures were measured in different units. Once standardised, β regression coefficients referred to the estimated change in a dietary outcome variable per standard deviation increase in the spatial exposure predictor variable. When it was not possible to calculate a standardised effect size, p-values or significance levels were used in combination with effect estimates as an indicator of the magnitude of each association. A quantitative, meta-analysis could not be performed due to the heterogeneity in outcome and spatial exposure measurements across studies. As such, between-study comparisons of effect size and significance were limited to qualitative, descriptive summaries (narrative synthesis). Within-study effect estimates were stratified by scale (i.e., the size of road network or Euclidean buffers) to ensure a valid comparison and account for the potential influence of geographic scale on measures of spatial exposure. Pairwise comparisons of all within-study effect estimates were made. To assess the within-study effect of availability versus accessibility measures on the relationship with diet, effect estimates from each study were stratified by food outlet type and dietary outcome to determine the largest within-study effect for each of these sub-groups. Stratification was done to allow for the examination of any differential associations that may exist between particular food outlet types and dietary outcomes.

### Study quality

An assessment of study quality was made for all included articles. This was done to obtain some measure of the methodological robustness of available within-study evidence. Criteria were based on questions from the National Institutes of Health (NIH) study quality assessment tools and selected to reflect quality concerns previously acknowledged to potentially alter study outcomes [10, 27, 30]. The scoring tool consisted of 8 criteria, with scores ranging from 0 to 3: *1. Data aggregation*: individual residential address or location (1); arbitrary administrative boundaries with aggregation of data (0). *2.* *Food outlet data source*: primary data source (i.e., field audit) (3); > one secondary data set (i.e., a combination of commercial and government sources or use of multiple online sites to obtain data) (2); single secondary data set (1); not reported or clearly specified (0). *3. Food outlet validation*: ground-truth validation (2); virtual verification (1); no validation or verification, not reported or clearly specified (0). *4. Food outlet classification*: classification based on standard government industry codes or own criteria clearly reported (2); own criteria not adequately described or justified (1); classification criteria not reported or clearly specified (0). *5. Study design*: ≤ 2 years between environment and participant data sources (2); temporal mismatch of > 2 years between environment and participant data sources (1); not reported or clearly specified (0). *6. Dietary assessment*: validated, quantitative assessment tool likely to represent usual dietary intake (i.e., food diary or validated food frequency questionnaire) (2); quantitative assessment tool not validated (1); not representative of usual intake (i.e., 24-h recall or single item questions), or not reported or clearly specified (0). *7. Response fraction*: > 70% (3); 70–50% (2); < 50% (1); not reported or clearly specified (0). *8. Data analysis*: adjustments made for relevant confounders (2); limited adjustment (1); no adjustment, not reported or clearly specified (0). Scores were weighted, whereby the score for each criterion was divided by the maximum possible value, so that each criterion had the same weighting in the final total score. Final scores were summed for each article and expressed as a percentage of the total possible, weighted score (n = 8). Other reviews have employed similar techniques to summarise study quality [10, 27, 30]. Assessment of sample sizes was based on previously applied cut-offs within the literature (0 = ≤ 50, 1 = 51–100, 2 = > 100) [36, 37]. Given that all studies had a sample size greater than 100, this quality criterion was excluded. Full scoring is available in Additional file 3. Study outcomes were examined in relation to quality scores via a scatter plot of the proportion of associations that were significant (%) against study quality score (%) for each article.

## Results

### Search results

**Fig. 1:**
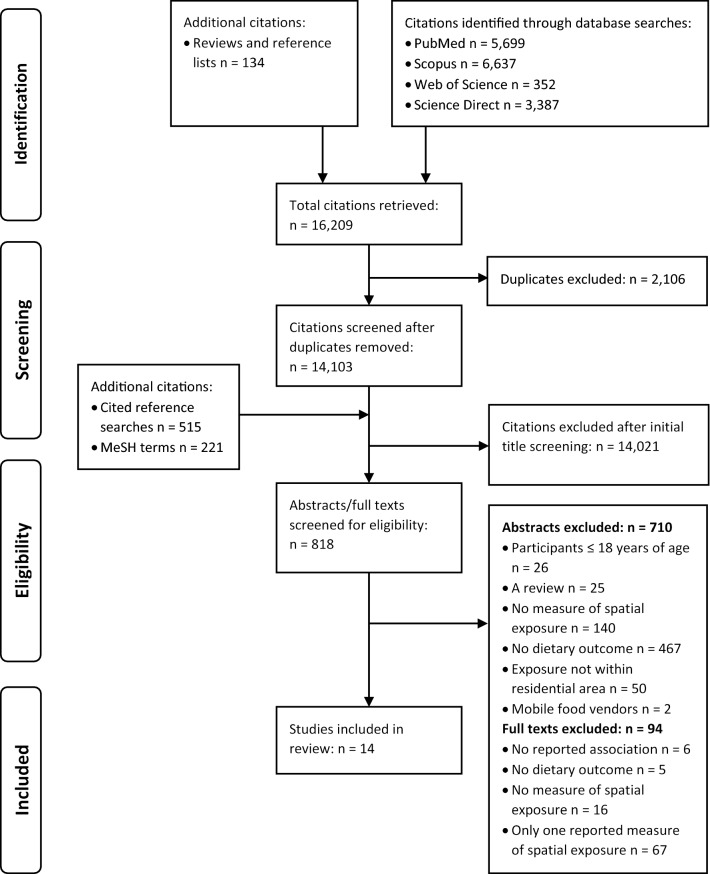
PRISMA flow diagram of the systematic search process

A total of 16,209 citations were retrieved, of which 2106 duplicates were removed based on a match between the title, authors, year of publication and Journal using reference software. A further 515 and 221 citations were retrieved from cited reference searches and MeSH searches, respectively. The titles of all remaining citations were initially screened with 14,021 excluded because they were irrelevant and outside the scope of this review. A remaining 818 citations were identified for abstract examination. A further 710 citations were excluded based on a review of the articles abstract, leaving 108 articles for full text review. Following full text review of the remaining 108 articles, we excluded studies that did not report on the statistical associations between spatial exposure measures and dietary outcomes (n = 6), did not incorporate a dietary outcome (n = 5), included no measure of spatial exposure (n = 16), or only reported on measures of accessibility *or* availability (n = 67) (i.e., they either examined the same spatial exposure measure at different buffer scales or examined multiple measures of availability *or* multiple measures of accessibility. A total of 14 articles were identified as eligible for inclusion within this review [23, 38–50] (Fig. 1). See Additional file 2 for a list of excluded citations with reason for exclusion.

### Study characteristics

The main characteristics of the 14 included articles are presented in Table 1. All were published post 2008, of cross-sectional design, and took place in urban (n = 10), mixed (n = 2) or rural (n = 2) areas. Most were conducted in the US (n = 6), with others in Australia (n = 3), Ireland (n = 1), Canada (n = 1) and the UK (n = 1), Brazil (n = 1) and Denmark (n = 1). Most (n = 10) used the home address of individual participants as the geographic location from which spatial exposure measures were determined. Following this, the nearest intersection to a participant’s home address was used (n = 1). Remaining articles utilised arbitrary administrative boundaries, either postal codes (n = 1), census collection districts (CCDs) (n = 1) or census blocks (n = 1) from which the geometric centroid or population weighted centroid was calculated and used to determine spatial exposure measures. Food outlet classifications were defined using several different approaches including government coding systems (n = 1), business names of commercial food chains (n = 2), Standard Industrial Classification Codes (n = 2), or unique systems based on author-defined features (n = 9), often with little justification or consistency among studies. This resulted in large variation in the definition and classification of food outlets. To locate food outlets, most articles utilised secondary data sources (n = 9), usually government databases or online commercial datasets. Sample sizes ranged from 102 to 48,305 participants. Studies frequently adjusted for common socio-demographic factors (e.g., sex, age, education and income). Less frequent adjustments were made for physical activity levels, weight status, car ownership, perceptions of the environment or area-level socio-economic status.

**Table 1:** Characteristics of included articles

First author (date)	Location	Study design (year of data collection)	Sample size (RF %)	Dietary outcome	Dietary assessment method	Food outlet	Food outlet classification	Food outlet data source	Geographic unit	Spatial exposure measure	Statistical analyses (adjustment variables)	Number of relationships	Number of associations	Study quality (%)
Athens (2016) [38]	Philadelphia and Baltimore, US	Cross-sectional, random digit dial (2009–2010)	1598 (11)	FF m/w	FFQ	FF S	Standard Industrial Classification codes, annual gross sales	Info USA 2011	Nearest intersection to participant’s home address	Count Presence Proximity	Negative binomial regression (time period, sex, race, age, education, census tract poverty level and population density)	2	14	65
Bodor (2008) [42]	New Orleans, US	Cross-sectional, Random digit dial (2001)	102 (50)	F s/d V s/d	24-h recall	S SS	Louisiana Office of Public Health annual gross sales codes	Louisiana Office of Public Health 2001, ground-truth validation 2001	Participant’s home address	Presence Proximity	Multivariable linear regression (sex, ethnicity, age, income, food assistance participation, car ownership)	4	8	75
Dunn (2012) [43]	Texas, US	Cross-sectional, Random digit dial, Brazos Valley Health Community Assessment, BVHA, rural, < 75 years (2006)	1064 (73.8)	FF m/w	FFQ	FF	Own criteria based on service style	Brazos Valley Food Environment Project (BVFEP) comprehensive ground-truth survey 2006	Participant’s home address	Count Proximity	Ordered logistic regression (census tract fixed effects (not stated), instrumental variable (IV) of shortest distance to major roadway)	1	3	94
Layte (2011) [44]	Ireland	Cross-sectional, Irish Survey of Lifestyle Attitudes and Nutrition, SLAN (2007)	7501 (72)	DASH score	Validated Willett FFQ	S CS	NR	NR	Participant’s home address	Count Proximity (Euclidean and network distance)	Fixed effects, ordinary least squares regression of participants with outlet within 2 km of home (sex, age, marital status, education, household income, population density, car ownership)	2	9	50
Minaker (2013) [45]	Ontario, Canada	Cross-sectional, neighbourhood environments in Waterloo Region Patterns of Transportation and Health, NEWPATH, women only (2009–2010)	1170 (64)	HEI-C	2-d food diary	R FS CS + S	Own criteria, NR	Local Public Health Inspection Database 2010, ground-truth survey 2010	Participant’s home address	Count Proximity Diversity RFEI	Multilevel linear regression (age, education, household income, car ownership, perceptions of food access and affordability)	3	6	81
Sharkey (2011) [46]	Texas, US	Cross-sectional, random digit dial Brazos Valley Health Community Assessment, BVHA, rural (2006)	1409 (73.8)	FF m/w	FFQ	TFF NFF TFF + NFF	Own criteria based on service style and place of consumption	Brazos Valley Food Environment Project (BVFEP) comprehensive ground-truth survey 2006	Participant’s home address	Count Proximity	Multivariable linear regression (sex, age, household income, race, BMI, household size, employment status)	3	12	94
Thornton (2012) [23]	Glasgow, UK	Cross-sectional, health and wellbeing survey, HWB (2002)	1041 (67)	FV s/d F s/d V s/d	FFQ	S	Six chain supermarket: Asda, the Co-op, Morrisons, Sainsbury’s, Somerfield, Tesco	Online yellow pages and company websites 2010, validated via street view and local knowledge	Participants’ post code	Count (Euclidean and network buffer) Presence (Euclidean and network buffer) Proximity Euclidean kernel density estimation	Multilevel linear regression (sex, age, education)	3	69	54
Thornton (2009) [47]	Melbourne, Australia	Cross-sectional, Victorian Lifestyle and Neighbourhood Environment Study, VicLANES (2003)	2547 (64)	FF purchase m/m Weekly Monthly	FFQ	FF	Five FF chains: Red Rooster, McDonalds, Kentucky Fried Chicken, Hungry Jacks, Pizza Hut	White pages phone directory 2003–2004	Participant’s home address	Count Proximity Variety	Multilevel multinomial regression (age, country of birth, household composition, education, occupation, income, attitudes and perceptions relating to food access; preference: taste and health, area-level disadvantage)	2	6	69
Turrell (2008) [48]	Brisbane, Australia	Cross-sectional, Brisbane Food Study (2000)	1001 (66.4)	TA purchase m/m	FFQ	FFF ITA ATA OTA C HTA STA	Own criteria based on preparation, service/sale method and main type of food sold	Brisbane City Council maps 2000, ground-truth survey 2000	Census Collection Districts	Proximity Average proximity Density	Ordered multinomial regression (sex, age, family size, country of birth)	7	21	69
Williams (2010) [49]	Melbourne, Australia	Cross-sectional, socioeconomic status and activity in women, SESAW (2004)	351 (58)	F s/d V s/d	FFQ	S FVS	Own criteria NR, supermarkets included major and minor chains, independent and small grocers	Local government and company websites, databases and online phone directories	Participant’s home address	Count Proximity	Logistic regression bivariate associations	4	8	48
Zenk (2009) [50]	Detroit, US	Cross-sectional, ≥ 25 years (2002–2003)	919 (55)	FV mean s/d	Semi-quantitative FFQ	S + GS	Own criteria NR, full-service chain grocery stores or super centres	Michigan Department of Agriculture 2001, paper/online telephone directories, company websites 2001–2002, ground-truth survey 2002	Census blocks	Presence Proximity	Two level hierarchical linear regression (sex, age, household size, years in neighbourhood, marital status, race, education, income, employment, car ownership)	1	2	67
LeDoux (2014) [41]	Detroit, US	Cross-sectional, low income African American neighbourhood, (NR)	258 (10.3)	FV s/m Soda and juice s/m Sweet and salty snacks s/m	FFQ	S CS FF	Own criteria clearly reported	Michigan Department of Agriculture, Detroit Economic Group, phone and internet directories, date NR	Participant’s home address	Count Proximity	Negative binomial regression (sex, age, education, household income, exercise)	9	27	44
Bernsdorf (2017) [39]	Copenhagen, Denmark	Cross-sectional, Danish Capital Regional Health Survey (2010)	48,305 (52.3)	FF ≥ once/w	FFQ	FF	Danish industrial classification system DB03, Own criteria clearly reported	Ministry of Environment and Food Register, ground-truth survey 2010	Participant’s home address	Count Proximity	Multilevel logistic regression (sex, age ethnicity, education, urbanicity, area SES)	1	8	77
Duran (2016) [40]	Sao Paulo, Brazil	Cross-sectional, (2011)	1842 (NR)	FV ≥ 5 d/w SSD ≥ 5 d/w	FFQ, validated	S + GS + FVS	Own criteria clearly reported	Ground-truth survey 2010–2011	Participant’s home address	Count Proximity	Poisson generalised estimating equations (sex, age, education, income)	2	12	75

*RF* response fraction (%), *NR* not reported, *F* fruit, *FF* fast-food, *V* vegetables, *FV* fruit and vegetables, *SSD* sugar sweetened drinks, *DASH* dietary approaches to stop hypertension, *HEI*-*C* healthy eating index adapted from Canada guidelines, *m/w* meals per week, *s/d* serves per day, *s/m* serves/month, *d/w* days per week, *m/m* meals per month, *FFQ* food frequency questionnaire, *S* supermarket, *GS* grocery store, *SS* small food store, *TA* takeaway, *CS* convenience store, *FFF* major chain fast food franchise, *ITA* general independent takeaway store, *ATA* Asian takeaway restaurant, *OTA* other ethnic takeaway restaurant, *C* café/coffee shop, *HTA* healthier takeaway store, *STA* sweet food takeaway, *FVS* fruit and vegetable store, *FS* food store, *R* restaurant, *TFF* traditional fast food, *NFF* non-traditional fast food, *BMI* body mass index (kg/m^2^), *SES* socioeconomic status

**Fig. 2:**
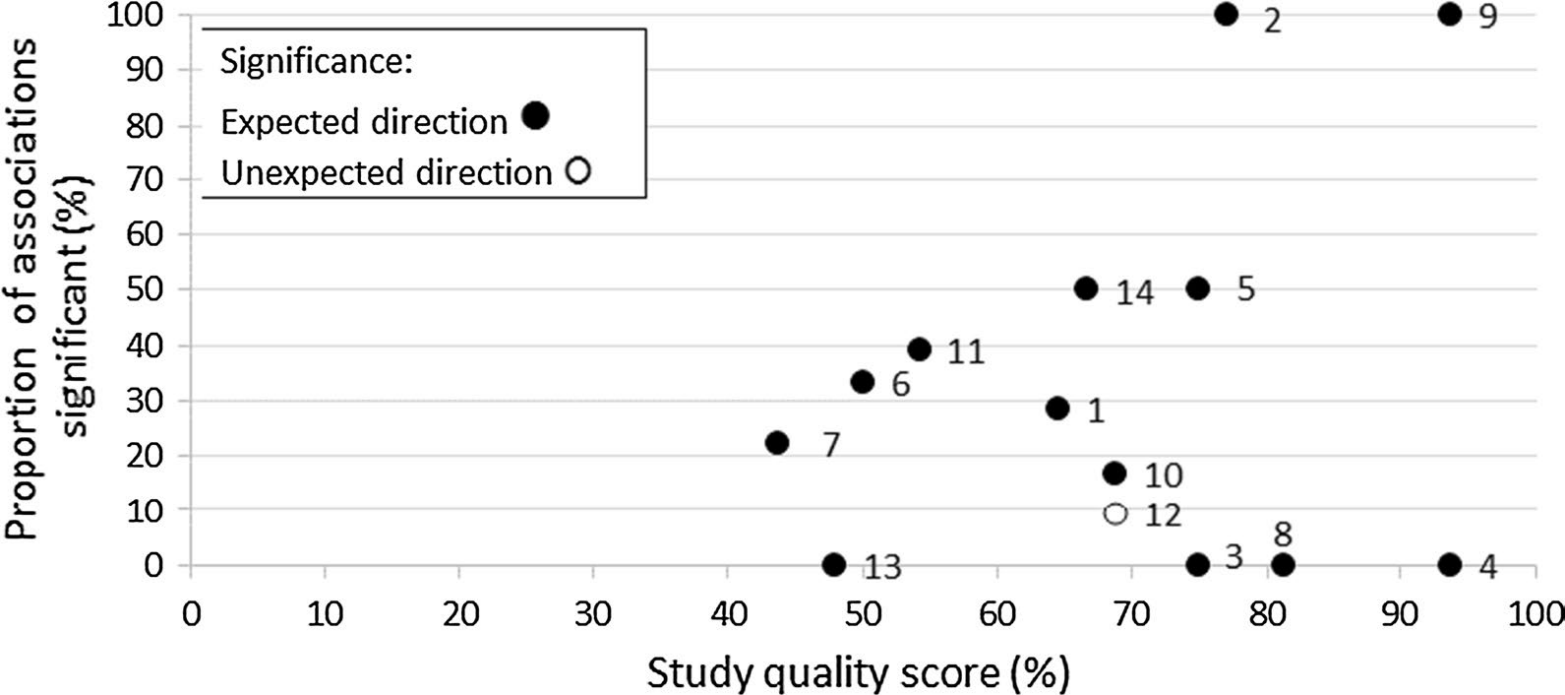
Relationship between the proportion of associations that were significant (%) and study quality score (%) for each article. Article 1: [38], 2: [39], 3: [42], 4: [43], 5: [40], 6: [44], 7: [41], 8: [45], 9: [46], 10: [47], 11: [23], 12: [48], 13: [49], 14: [50]

Study quality scores ranged from 44 to 94%, and most articles scored in the upper third of the scale (Table 1). There was no visible trend in study quality scores by study outcomes, with those articles reporting significant associations having a similar range of quality scores compared to those articles that reported only null findings (Fig. 2).

### Summary of extracted associations and relationships

Many articles examined multiple dietary outcomes and food outlet types. As such, the number of extracted associations refers to the individual association between a particular spatial measure, food outlet type and dietary outcome. Whereas an extracted relationship refers to the collection of associations for a particular food outlet type and dietary outcome (involving more than one spatial exposure measure). Therefore, within a study, the number of relationships is the number of different dietary outcomes multiplied by the number of different food outlet types examined (Table 1).

#### Extracted associations

A total of 205 individual associations were extracted, each relating to a particular spatial exposure measure (155 availability and 50 accessibility), food outlet type and dietary outcome. Extracted effect estimates for associations included the odds ratio (OR) [39, 40, 47, 49], unstandardised beta (β) regression coefficients [23, 41–46, 48, 50], and percent change [38] and in all cases these were converted to a standardised (where possible) or unstandardised β coefficient. For reported percent change, β = ln (1 + percent change/100). Six articles published sufficient information to calculate standardised β estimates [23, 38, 43, 46, 48, 50].

**Fig. 3:**
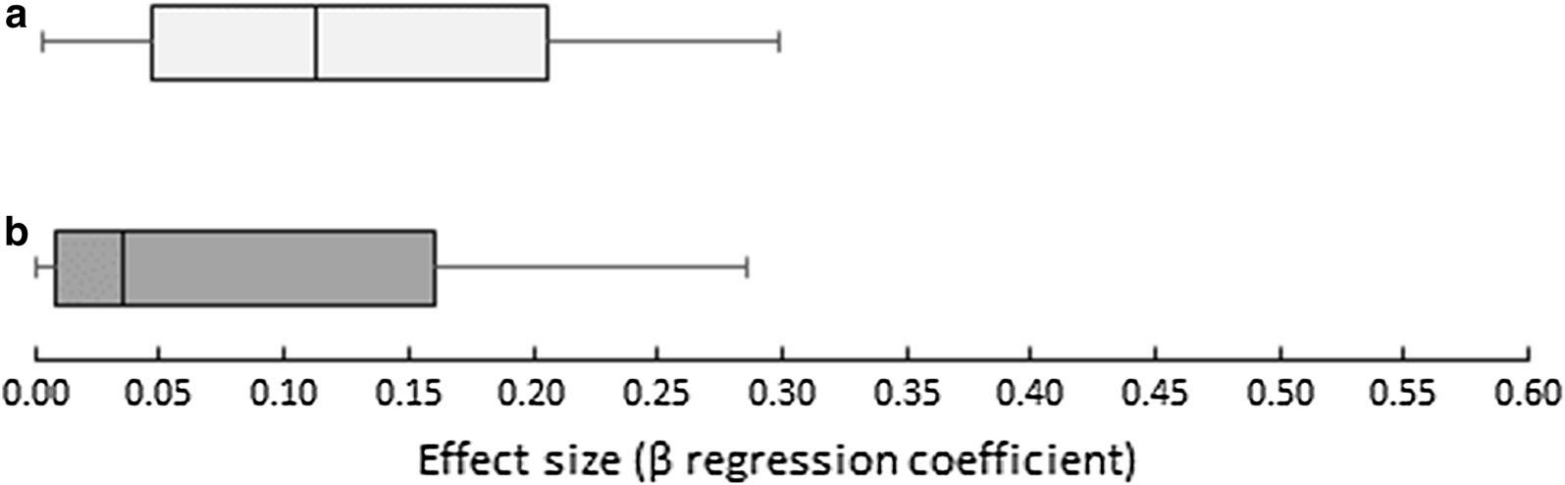
Boxplots displaying the spread of extracted effect sizes from **a** availability and **b** accessibility measures across all studies

Overall, effect sizes were relatively small with 76% (n = 156/205) of associations having an effect estimate (β) less than 0.2 and 33% (n = 68/205) reaching statistical significance, most of which (78%) (n = 53/68), were availability measures comprising mostly of counts in road network buffers and counts in Euclidean buffers (38/53), or Euclidean kernel density estimations (15/53). Figure 3 shows the spread of availability versus accessibility measures across all studies. With the exception of four outliers (4.551, 3.187, 2.240 and 2.120), effect sizes from accessibility measures tended to be smaller (median = 0.035) than availability measures (median = 0.113).

Across all associations (n = 205), the proportion that were significant in the expected direction varied by spatial exposure measure; 61.1% (11/18) that used a Euclidean kernel density estimations were significant, 44% (30/68) involving counts in road network buffers were significant, and 40% (8/20) that used counts in Euclidean buffers were significant (Table 2). For remaining measures, less than 40% of associations were significant in the expected direction with the exception of variety but this consisted of only two associations.

**Table 2:** Description of spatial exposure measures from extracted relationships and associations

Concept	Measure		Description	Number of relationships^a^	Number of associations (n = 202)^b^	% of associations significant in the expected or unexpected direction^c^
Availability	Continuous density	Euclidean kernel density estimation	A continuous density surface across a given area based on defined Euclidean kernels calculated using a probability density function	3	18	61.1
	Variety	Road network buffer	Number of different outlets belonging to the same outlet category type within a defined area calculated using a road network radius from a reference location	2	2	50
	Count	Road network buffer	Number of outlets within a defined area calculated using a road network radius from a reference location	26	68	44
		Euclidean buffer	Number of outlets within a defined circular area calculated using a straight line radius from a reference location	5	20	40
	Presence	Euclidean buffer	Binary presence/absence (1, 0) of outlets within a defined circular area calculated using a straight line radius from a reference location	8	11	9
		Road network buffer	Binary presence/absence (1, 0) of outlets within a defined area calculated using a road network radius from a reference location	5	12	8
	Relative density	Euclidean buffer count/10,000 population	Number of outlets relative to another quantity within a defined circular area calculated using a straight line radius from a reference location	9	13	7.7
	Diversity	Euclidean buffer	Number of different outlet category types within a defined circular area calculated using a straight line radius from a reference location	1	1	0
	Retail food environment index	Euclidean buffer	Ratio of different outlet types within a defined circular area calculated using a straight line radius from a reference location	1	1	0
Accessibility	Proximity	Euclidean distance	Straight line distance from a reference location to the nearest outlet	7	11	36.4
		Road network distance	Road network distance from a reference location to the nearest outlet	36	38	21 (2.6)
	Average proximity	Road network distance	Average road network distance from a reference location to all outlets	7	7	(28.6)

^a^Count of the total number of relationships that employed a particular measure

^b^Dunn (2012) [43] did not report on the technique used to determine proximity, count within a 1 mile buffer, and count within a 3 mile buffer. Therefore, these associations (n = 3) were excluded from the table

^c^The percentage of unexpected associations is placed in brackets

#### Extracted relationships

44 relationships were extracted that involved more than one spatial exposure measure to assess the association between a particular food outlet type and dietary outcome (Tables 1 and 2). The most common dietary outcomes were fruit and/or vegetable intake (n = 16/44) and fast food intake (n = 7/44) assessed via food frequency questionnaires. Five relationships examined a measure of diet quality [i.e., dietary approaches to stop hypertension (DASH) score and Canadian Healthy Eating Index (HEI-C)]. Remaining relationships examined takeaway purchase (n = 7/44), soda and juice intake (n = 3/44), sweet and salty snack intake (n = 3/44), fast food purchase (n = 2/44), and sugar sweetened drinks (n = 1/44).

A total of 16 different food outlet types were examined across all relationships, with fast food outlets (n = 11/44) and supermarkets (n = 15/44) the most commonly used classifications. Others included grocery stores, fruit and vegetable stores, small food stores, convenience stores, and takeaway stores. Most spatial exposure measures were determined relative to a single food outlet type with the exception of one article that examined the diversity and a ratio based on the retail food environment index (RFEI). No study examined the combined total of all food outlet types present.

Table 2 provides a description of the 12 different measures of spatial exposure to the CFE. Accessibility measures consisted of proximity (n = 43/44) and average proximity (n = 7/44), whilst availability measures consisted of a diverse range of measures with counts being the most prevalent (n = 31/44). Relative density, variety, diversity and the RFEI were less frequently employed as were more complex measures involving probability density functions with only three relationships utilising Euclidean kernel density estimations to derive a continuous density measure [23]. Most relationships employed between two and four different spatial exposure measures.

### Assessment of within-study effects

Of the 44 relationships, 18/44 (41%) found at least one statistically significant association with 16/44 of these being in the expected direction (36%). Of those 16 relationships, 8 had a significant association involving only an availability measure and 8 had a significant association for both an availability and accessibility measure. The largest overall effect size from each relationship consisted of the availability measures of count (n = 18/44), presence (n = 4/44), diversity (n = 1/44), relative density (n = 1/44) and variety (n = 1/44), and accessibility measures proximity (n = 13/44) and average proximity (n = 6/44).

Table 3 provides a summary of all within-study pairwise comparisons of spatial exposure measures, stratified by relationship and scale (n = 176). The most frequently compared measures within a study were counts in road network buffers with proximity road network distances (n = 78/176), with counts in road network buffers having the greatest effect size in 77% (60/78) of comparisons. Following this, 19 pairwise comparisons involved counts in Euclidean buffers with proximity road network distances, and counts in Euclidean buffers had the greatest effect size in 79% (15/19) of pairwise comparisons. Overall, availability measure had the greatest effect size in 139 of the 176 pairwise comparisons.

**Table 3:** Summary of within-study pairwise comparisons of availability versus accessibility measures

Pairwise comparison	Total	Measure with greatest effect size (percentage of total)
*Count versus proximity*	*100*	*Count (75%)*
Count road network buffer Proximity road network distance	78	Count road network buffer (77%)
Count Euclidean buffer Proximity road network distance	19	Count Euclidean buffer (79%)
Count road network buffer Proximity Euclidean distance	3	Proximity Euclidean distance (100%)
*Presence versus proximity*	*23*	*Presence (74%)*
Presence road network buffer Proximity road network distance	12	Presence road network buffer (100%)
Presence Euclidean buffer Proximity road network distance	7	Presence Euclidean buffer (71%)
Presence Euclidean buffer Proximity Euclidean distance	4	Proximity Euclidean distance (100%)
*Continuous density versus proximity*	*18*	*Continuous density (89%)*
Proximity road network distance Euclidean kernel density estimation	18	Euclidean kernel density estimation (89%)
*Relative density versus proximity*	*25*	*Density Euclidean buffer/10,000 population (76%)*
Density Euclidean buffer/10,000 population Proximity Euclidean distance	18	Density Euclidean buffer/10,000 population (67%)
Density Euclidean buffer/10,000 population Proximity road network distance	7	Proximity road network distance (100%)
*Relative density versus average proximity*	*7*	*Average proximity (100%)*
Density Euclidean buffer/10,000 population Average proximity road network distance	7	Average proximity road network distance (100%)
*Variety versus proximity*	*2*	*Variety and proximity (50%)*
Variety road network buffer Proximity road network distance	2	50%
*RFEI versus proximity*	*1*	*Proximity (100%)*
RFEI Euclidean buffer Proximity road network distance	1	Proximity road network distance (100%)

Dunn (2012) [43] did not report on the technique used to determine proximity, count within a 1 mile buffer, and count within a 3 mile buffer. Therefore, these associations (n = 3) were excluded from the table

Results varied by food outlet type and dietary outcome measure (see Additional file 4 for a full list of extracted effect sizes). Sixteen relationships (i.e., 16/44) examined either fruit, vegetable, or fruit and vegetable intake, and proximity (accessibility) to supermarkets, small stores and fruit and vegetable stores had the greatest within-study effect size (n = 8/16) followed by the availability measures counts (n = 6/16) and presence (n = 2/16) (Additional file 4a). Fourteen relationships (i.e., 14/44) examined fast food or unhealthy food intake (including soda and juice; sweet and salty snacks), and a count of fast food outlets or convenience stores frequently had the greatest within-study effect on fast food intake (n = 4/14) or unhealthy food intake (n = 4/14), most of which were significant (Additional file 4b). The proximity to takeaway food outlets frequently had the greatest within-study effect on takeaway purchase (Additional file 4c), however all comparisons were from the same study population. Only one article (two relationships) examined fast food purchase, with proximity road network distance to fast food outlets and variety of fast food outlets in road network buffers having the greatest effect on fast food purchase (Additional file 4c). There was no apparent trend in within-study effects of spatial exposure measures and diet quality, yet all five relationships involved differing food outlet types and comparative spatial exposure measures (Additional file 4d).

## Discussion

### Summary of key findings

This systematic review identified 14 articles that met eligibility criteria, from which 44 distinct relationships and 205 individual associations were extracted. The main aim was to determine what influence the different spatial exposure measures of availability and accessibility had on the CFE-diet relationship, by examining within-study effects. This review highlights several key findings including: (1) the overall small statistical effect sizes for associations between spatial exposure to food outlets and dietary outcomes; (2) how few studies have utilised more than one type of spatial exposure measure of the food environment; (3) that availability measures (opposed to accessibility measures) may be more likely to produce statistically significant and greater effect sizes; and (4) the need for future studies to consider the comparative effect on dietary outcomes of spatial exposure measures derived from place-based versus people-based approaches.

Overall, extracted effect sizes were relatively small, but availability measures tended to produce larger effect sizes than accessibility measures and were more likely to reach statistical significance. The most commonly compared measures within studies were counts in road network buffers with proximity road network distances. Within studies involving both availability *and* accessibility measures, availability measures were more likely to reach statistical significance in comparison to accessibility measures. Furthermore, the greatest within-study effect sizes consisted mostly of availability measures and largely from counts in road network buffers. However, results varied by food outlet type and dietary outcomes. Proximity to supermarkets, small stores and fruit and vegetable stores frequently had the greatest within-study effect on fruit, vegetable, and fruit and vegetable intake. Whereas, a count of fast food outlets and convenience stores frequently had the greatest within-study effect on fast food intake and unhealthy food intake. Despite the variation in study quality across the 14 included articles, there was no relationship between study quality and the proportion of associations found to be significant for each article. Other reviews examining the relationship of the CFE with obesity have also found no influence of study quality on results [30].

### Implications for research and practice

Few studies have employed multiple measures of spatial exposure to examine the CFE-diet relationship. As seen in previous reviews [5], the most frequently applied measures were counts and proximity. Findings suggest that both accessibility (e.g., proximity) and availability (e.g., counts) measures are important concepts to consider when measuring spatial exposure to the CFE since they may produce differing effects depending on food outlet type and dietary outcome measures. However, counts tended to return more robust associations with dietary intake. Thus, the number of available food outlets and concepts such as choice and concentration may have a greater influence on diet than the distance required to travel to the closest food outlet. This was more apparent for fast food intake, suggesting living in an area where there are more fast food outlets available may impact fast food intake more than proximity alone. This has been suggested elsewhere, with a greater percentage of studies from a recent review finding a significant association between density rather than proximity to fast food outlets and unhealthy dietary outcomes [3]. This has implications when establishing evidence-based CFE planning and policy interventions since limiting the density of fast food outlets within residential areas may represent a promising strategy for improving population dietary choices.

Yet our findings demonstrate how measures of spatial exposure to the CFE may influence dietary choices differentially, depending on the food outlet type, highlighting the need for a multi-method approach when measuring spatial exposure. For example, we observed that proximity to a supermarket may be more important than the number of supermarkets available when it comes to fruit and vegetable intake. However, natural experiments examining the influence of opening a new supermarket have shown mixed findings [51–56] indicating the relationship between supermarkets and dietary intake is more complex than proximity alone and probably involves multiple factors such as shopping preference, available transport links, access to a motor vehicle, and the presence of other food outlets.

Overall, most effect estimates were relatively small, and over half of extracted associations reported null results only. It is likely that the effects of spatial exposure measures on dietary intake are small, relative to a range of other factors such as within-store characteristics [57], individual preferences [58] and perceptions of access and availability [59]. These may all be meaningful concepts with small to moderate effects, operating in combination, emphasising the complex relationship between the food environment and dietary choices [2, 60]. Moreover, associations between spatial exposure to food outlets and dietary outcomes may be subtly moderated by individual characteristics such as gender, age, education, income and marital status.

Alternatively, the small effect sizes may relate to the methods used to measure exposure. Studies involving within-store assessments of available food sources [61, 62] or use of people-based activity spaces to define exposure [63–65] have shown positive associations with dietary outcomes. These approaches provide a more accurate assessment of an individual’s daily exposure to food sources and are being recognised as the preferred best-practice within the field. Emerging findings from these studies, in comparison to the mostly null findings in this review, may serve to illustrate how the widely used place-based spatial exposure measures of proximity and count to arbitrarily defined food outlet classifications fail to accurately operationalise exposure. From a policy perspective, this has implications when interpreting and synthesising the existing literature as a large majority of the findings may not be representative of actual lived-experiences given the methods used and thus the results should be interpreted with caution. All studies in this review had at least one methodological limitation (e.g., inconsistent classification of food outlet types, lack of validation of food outlet data, and use of error-prone dietary assessment methods), most of which have been extensively cited elsewhere in food environment research [3, 4, 10–12, 25, 66–69]. Too much measurement error in the dependent and independent variables and a largely cross-sectional evidence-base may also be influencing effect sizes.

It is still common for researchers to examine associations between dietary outcomes and spatial exposure to food outlets derived from place-based measurements as this is often the most feasible and realistic approach for large, population based studies [70]. Therefore, improving study designs, and working towards addressing common methodological issues will serve to reduce error and improve precision in place-based measures spatial exposure. Furthermore, where possible, the findings from best-practice research should be used to inform the way ‘neighbourhood’ is operationalised and how food outlets are defined and classified in future studies.

### Strengths and limitations of this review

This review provides the first summary of studies to date to consider the effect of differences in spatial exposure measures when examining the CFE-diet relationship. Previous reviews of the CFE-diet relationship have not distinguished their findings from studies with only one measure and studies with more than one measure of spatial exposure. Given the methodological heterogeneity among studies, summaries made across studies are less robust and subject to bias associated with the ways in which those studies were undertaken. However, by making within-study comparisons, our review provides more reliable findings of the CFE-diet relationship.

Within-study comparisons of standardised β regression coefficients have not been reported in earlier reviews of this nature, and our review is the first to our knowledge to compare within-study effect sizes of different measures of spatial exposure to food outlets. When comparing within-study effects, this review accounted for the scale at which availability measures were derived (i.e., differing buffer sizes) as previous work has indicted the presence of potential scale effects on exposure measures [71, 72]. Previous reviews examining the CFE-diet relationship have not stratified their findings by scale. Further, our review included a measure of study quality which is lacking in the majority of previously published reviews with no previous reviews involving dietary outcomes having measured study quality.

However, several inconsistent methodological issues and contextual differences limited the interpretability of findings. Summaries of effect sizes and statistical significance across studies were subject to bias associated with variation in dietary assessment methods [73]. For example, diet quality indices, (i.e., DASH and HEI-C), are more complex measures subject to greater variance and error versus simple frequency questions, influencing relationships with spatial exposure. Inconsistency in the source, validation, classification and aggregation of food outlet data and derived spatial exposure measures was also likely to influence study outcomes and remains a challenge in the field of food environment research when combining evidence. As such, variance in the outcomes and effect sizes between studies was likely confounded by how those studies were conducted. The statistical findings from included studies may not apply to all individuals, particularly for those studies that made use of large population data sets. The relationships between spatial exposure to food outlets and dietary intake may vary for particular sub-groups or individuals with certain demographic characteristics [74]. Furthermore, it was not possible to standardise all effect sizes, so conclusions regarding the comparative magnitude of some within-study effects should also be interpreted with caution.

Summaries of findings across studies were dominated by the prevalent measures of spatial exposure (i.e., count and proximity). Similarly, within-study pairwise comparisons were dominated by those from one study population [23]. Several spatial exposure measures were less prevalent (i.e., variety, diversity, RFEI and Euclidean kernel density estimations), with minimal comparisons preventing valid conclusions. Limited within-study comparisons involving diet quality, takeaway purchase and fast food purchase, prevented valid conclusions regarding the influence of spatial exposure measurement on these dietary outcomes. More research is required to clarify potential effects, if any, involving these less prevalent exposure measures and dietary outcomes. Furthermore, included studies often employed more than one availability measure, this could likely mean more chances for an effect to be significant, thus contributing to the greater number of significant effects belonging to availability measures.

Results presented in this review were for associations extracted from studies in which the statistical outcomes were provided. Often when studies examined multiple associations using different dietary outcomes, food outlet types and exposure measures, only significant associations were reported, so it was not possible to include these comparisons within this review. Given the preference towards publication of significant results, the omission of non-significant results from multi-method studies is undesirable, creating a biased evidence base and preventing any evaluation of the effect of spatial exposure measurement on study outcomes. Furthermore, no studies included in this review made reference to the consideration of spatial autocorrelation or applied spatial regression and/or analytical techniques. Previous work has identified that the spatial nature of data is infrequently acknowledged or accounted for in analyses within this field of research [75]. The presence for spatially correlated residuals could violate the assumptions of traditional regression methods and influence results, thus altering the findings of this review.

Finally, this review focused on the most commonly used methodology and evaluated studies that assessed exposure to food outlets around the home or within residential areas, in adults and involving dietary outcomes. Findings may differ with different outcomes (i.e., weight status) or for different population groups (i.e., children). New emerging methodological techniques involving the use of global positioning systems (GPS) to track individuals and determine ‘total activity spaces’, or places frequently visited is a developing area [17, 76, 77]. Studies have begun to examine the links between diet and GPS-derived exposure measures, yet findings so far are equally mixed [76, 77]. This review identified no studies that provided a within-study comparison of availability *and* accessibility spatial exposure measures involving the use of total activity spaces. Although GPS technologies offer potential for determining more valid measures of exposure, certain limitations in terms of costs, feasibility within large sample sizes, user compliance and level of processing complexity of GPS data may presently limit their widespread use [73]. Indeed, most research to-date has measured exposure to food outlets relative to an individual’s home address [77] as this represents a feasible approach for quantifying exposure–outcome relationships across large spatial and temporal scales for use in policy and urban planning.

### Recommendations

This review highlights the limited number of studies, relative to the wider literature [3], which have examined and reported on the potential for different measures of spatial exposure to moderate observed CFE-diet relationships. There is still no consensus on the use of different exposure measures within the field of food environment research. Therefore, when employing such measures to examine the CFE-diet relationship, a multi-method approach is recommended. Inclusion of more than one spatial exposure measure is likely to provide a more comprehensive description of exposure by capturing multiple aspects of availability and accessibility. Given the effects of spatial exposure may vary depending on food outlet type, dietary outcome and contextual factors, researchers should aim to include measures that are relevant for different population groups and settings by specifying a priori hypotheses relating to the conceptualisation of exposure. When multiple measures are employed, studies should report on any sensitivity analyses or include observed effect sizes and p-values, to allow researchers to evaluate the importance of results or any non-significant effects. Identifying and providing statistical information for a range of metrics associated with diet will support the development of planning policy and urban design guidelines and serve towards the development of standardised indicators of exposure.

Few studies have examined the use of GPS derived activity spaces or more alternative measures such as kernel density estimations, spatial interaction models and travel time/cost models together with more frequently employed, traditional measures such as proximity and counts within place-based buffers. These alternative approaches of deriving spatial exposure show promise when examining disparities in the availability and accessibility of food outlets [78] and links with diet [23, 79]. No study has examined the comparative performance of spatial interaction models and more traditional measures of spatial exposure, despite their advantages and demonstrated success within other fields of research. For example, in physical activity research, gravity models have been applied to investigate the relationship between public open space and walking [80]. The use of kernel density estimations and spatial interaction models also serve to overcome certain methodological challenges such as defining ‘neighbourhood’ areas and the associated uncertain geographic context problem [81]. Spatial interaction models allow for the incorporation of multiple concepts likely to influence exposure in addition to distance such as quality, attractiveness and size of food outlets. Comparative studies exploring how the use of place-based measures of exposure and less frequently applied methods of spatial modelling moderate exposure-diet relationships will provide further methodological insights.

## Conclusions

This systematic review summarised the within-study evidence from 14 articles to determine the effect of different spatial exposure measures on dietary outcomes. The limited evidence suggests that availability measures may be more likely to produce statistically significant and greater effect sizes than accessibility measures. However, the greater use of availability measures may have contributed to this finding. Furthermore, this may vary depending on the food outlet type and dietary outcomes examined. Findings suggest that proximity to a supermarket (accessibility) may be more important than the count or presence (availability). Whereas a count of fast food and/or convenience stores may influence unhealthy food intake more than the proximity. More research is required to explore the methodological effects of less prevalent exposure measures (e.g., involving the use of GPS derived activity spaces and spatial modelling), food outlet types and dietary outcomes within specific sub-populations and contextual settings. Furthermore, reporting on the results of multi-method studies is needed to differentiate findings by the type of spatial exposure measure, which will establish evidence for the appropriateness of each measure and help discern those which may be more relevant under certain circumstances. These findings will ultimately serve to provide greater clarity and insight into appropriate targets for policy and urban planning aimed at improving dietary outcomes.

## Additional files

**Additional file 1.** PRISMA checklist. PRISMA checklist.

**Additional file 2.** Search strategy. Search strategy and list of excluded citations.

**Additional file 3.** Study quality scores. Criteria and scoring system used to determine study quality.

**Additional file 4a.** Summary of associations with fruit, vegetable, and fruit and vegetable intake by food outlet type *. **b**. Summary of associations with fast food and unhealthy food intake by food outlet type *. **c.** Summary of associations with takeaway purchase and fast food purchase by food outlet type *. **d.** Summary of associations with diet quality by food outlet type*. Summary of extracted effect sizes and p values for associations between food outlet exposure and dietary outcomes.

## Abbreviations

ATA: Asian takeaway restaurant
BMI: body mass index
C: café/coffee shop
CCD: census collection district
CFE: community food environment
CI: confidence interval
CS: convenience store
d/w: days per week
DASH: dietary approaches to stop hypertension
F: fruit
FF: fast food
FFF: major chain fast food franchise
FFQ: food frequency questionnaire
FS: food store
FV: fruit and vegetables
FVS: fruit and vegetable store
GIS: geographic information systems
GS: grocery store
HEI-C: Canadian Healthy Eating Index
HTA: healthier takeaway store
ITA: general independent takeaway store
m/m: meals per month
m/w: meals per week
NFF: non-traditional fast food
NR: not reported
OR: odds ratio
OTA: other ethnic takeaway restaurant
R: restaurant
RF: response fraction
RFEI: retail food environment index
S: supermarket
s/d: serves per day
SD: standard deviation
SE: standard error
SEM: standard error of the mean
SES: socio-economic status
SS: small food store
STA: sweet food takeaway
TA: takeaway
TFF: traditional fast food
V: vegetables
