# Supplementary material for: Food environments and dietary intakes among adults: does the type of spatial exposure measurement matter? A systematic review
Source: Int J Health Geogr. 2018 Jun 9;17:19. doi: 10.1186/s12942-018-0139-7 (PMC5994245; doi:10.1186/s12942-018-0139-7)
Supplement: Supplementary file 2 — Additional file 2. Search strategy. Search strategy and list of excluded citations. [file 12942_2018_139_MOESM2_ESM.docx]

Research question: *Does the choice of within-study spatial exposure measure (availability versus accessibility) influence associations between the community food environment and diet?*

**Combinations of the following search terms**

| **Search concept** | **Terms** |
| --- | --- |
| Search concept 1: exposure | density OR proximity OR GIS OR geographic OR spatial OR exposure OR access* OR location |
| Search concept 2: environment | “food environment” OR neigh* OR “built environment” OR retail OR outlet* OR store* OR “nutrition environment” OR foodscape OR supermarket* OR shop* |
| Search concept 3: outcome | diet* OR intake OR fruit OR vegetable OR food OR consumption OR purchase OR health* OR nutrition |

**Results of search strategy**

| **Database searches** | | | | | |
| --- | --- | --- | --- | --- | --- |
| **Database** | **PubMed** | **Scopus** | **Web of Science** | **Science Direct** | |
| **Fields** | Title, Abstract | Article title, Abstract, Keywords | Title | Title, Abstract, Keywords | |
| **Limits** | - Journal articles - Humans - English language - Publication date 1980 - 31/12/2017 | - Journal articles - English language - Published 1980 - 2017 - Subject areas: medicine, social sciences, environmental sciences, health professionals, multidisciplinary, undefined | - Timespan 1980-2017 - English language | - Timespan 1980-2017 - Journal articles | |
| **Date** | **16/01/2018** | **16/01/2018** | **16/01/2018** | **16/01/2018** | |
| Combined: 1 AND 2 AND 3 AND NOT (child* OR school* OR adolescent*) | 5,699 | 6,637 | 352 | 3,387 | |
| **Reference harvesting** | | | | | |
| **Citations retrieved from reference lists of included articles and the following reviews:** | | | | | **# of citations retreived** |
| - McKinnon et al 2009 - Lytle et al 2017 - Rahmanian et al 2014 - Mhurchu et al 2013 - Larson and Story 2009 - Kamphuis et al 2006 - Giskes et al 2011 - Giskes et al 2007 - Fraser et al 2010 - Fleischhacker et al 2011 - Charreire et al 2010 - Caspi et al 2012 - Casagrande et al 2009 - Black et al 2014 - Beaulac et al 2009 | | | | | 134 |
| **Final citations retrieved: 5,699 + 6,637 + 352 + 3,387 +134 = 16,209** | | | | | |

| **Citation searching in the Scopus database (10/04/2018)** | |
| --- | --- |
| **Reference** | **# of citations** |
| Thornton et al 2012 | 38 |
| Athens et al 2016 | 1 |
| Bernsdorf et al 2017 | 0 |
| Duran et al 2016 | 5 |
| LeDoux et al 2014 | 10 |
| Bodor et al 2008 | 228 |
| Dunn et al 2012 | 37 |
| Layte et al 2011 | 24 |
| Minaker et al 2013 | 31 |
| Sharkey et al 2011 | 24 |
| Thornton at al 2009 | 54 |
| Turrell et al 2008 | 35 |
| Williams et al 2010 | 40 |
| Zenk et al 2009 | 132 |
| **Total** | **659** |
| **Duplicates removed** | **144** |
| **Excluded based on title/abstact (out of study scope)** | **469** |
| **Excluded based on full text review** | **46** |

| **MeSH searches in the PubMed database (13/04/2018)** | |
| --- | --- |
| **Commbinations of MeSH terms** | **# of citations** |
| "Environment Design"[Mesh] AND "Spatial Analysis"[Mesh] | 53 |
| "Food Analysis"[Mesh] AND "Food Supply"[Mesh] | 168 |
| **All retrieved citations (221) were excluded based on review of the title/abstact (out of study scope)** | |

**List of excluded articles**

| **Citation excluded based on abstract review** | **Reason** |
| --- | --- |
| 1. Abusabha, R., Namjoshi, D., & Klein, A. Increasing access and affordability of produce improves perceived consumption of vegetables in low-income seniors. *J Am Diet Assoc*. 2. Adachi-Mejia, A. M., Lee, C., Lee, C., Carlos, H. A., Saelens, B. E., Berke, E. M., & Doescher, M. P. Geographic variation in the relationship between body mass index and the built environment. *Preventive Medicine*. 3. Adam, A., & Jensen, J. D. What is the effectiveness of obesity related interventions at retail grocery stores and supermarkets? - a systematic review. *BMC Public Health*. 4. Adams, A. T., Ulrich, M. J., & Coleman, A. Food deserts. *Journal of Applied Social Science*. 5. Adams, J., Halligan, J., Watson, D. B., Ryan, V., Penn, L., Adamson, A. J., & White, M. The Change4Life Convenience Store Programme to Increase Retail Access to Fresh Fruit and Vegetables: A Mixed Methods Process Evaluation. *PLoS One*. 6. Adebayo, A. A., Musvoto, G. G., & Adebayo, P. Towards the Creation of Healthier City Neighbourhoods for Marginalised Communities in South Africa: A Case Study of the South Durban Industrial Basin in the City of Durban. *Urban Forum*. 7. Adlakha, D., Hipp, A. J., Marx, C., Yang, L., Tabak, R., Dodson, E. A., & Brownson, R. C. Home and workplace built environment supports for physical activity. *Am J Prev Med*. 8. Affuso, O., Singleton, C. R., Brown, S. C., Perrino, T., Huang, S., & Szapocznik, J. Associations between neighborhood socioeconomic environment and physical activity in Cuban immigrants. *SSM - Population Health*. 9. Aggarwal, A., Rehm, C. D., Monsivais, P., & Drewnowski, A. Importance of taste, nutrition, cost and convenience in relation to diet quality: Evidence of nutrition resilience among US adults using National Health and Nutrition Examination Survey (NHANES) 2007–2010. *Preventive Medicine*. 10. Ainsworth, B. E., Wilcox, S., Thompson, W. W., Richter, D. L., & Henderson, K. A. Personal, social, and physical environmental correlates of physical activity in African-American women in South Carolina. *Am J Prev Med*. 11. Alaimo, K., Packnett, E., Miles, R. A., & Kruger, D. J. Fruit and vegetable intake among urban community gardeners. *J Nutr Educ Behav*. 12. Albert, S. L., Langellier, B. A., Sharif, M. Z., Chan-Golston, A. M., Prelip, M. L., Garcia, R. E., . . . Ortega, A. N. A corner store intervention to improve access to fruits and vegetables in two Latino communities. *Public Health Nutrition*. 13. Albrecht, S. S., Osypuk, T. L., Kandula, N. R., Gallo, L. C., Le-Scherban, F., Shrager, S., & Diez Roux, A. V. Change in waist circumference with longer time in the United States among Hispanic and Chinese immigrants: the modifying role of the neighborhood built environment. *Ann Epidemiol*. 14. Algert, S. J., Agrawal, A., & Lewis, D. S. Disparities in access to fresh produce in low-income neighborhoods in Los Angeles. *Am J Prev Med*. 15. AlHasan, D. M., & Eberth, J. M. An ecological analysis of food outlet density and prevalence of type II diabetes in South Carolina counties. *BMC Public Health*. 16. Aloia, C. R., Gasevic, D., Yusuf, S., Teo, K., Chockalingam, A., Patro, B. K., . . . Lear, S. A. Differences in perceptions and fast food eating behaviours between Indians living in high- and low-income neighbourhoods of Chandigarh, India. *Nutr J*. 17. Alter, D. A., & Eny, K. The relationship between the supply of fast-food chains and cardiovascular outcomes. *Canadian Journal of Public Health*. 18. Alves, L., Silva, S., Severo, M., Costa, D., Pina, M. F., Barros, H., & Azevedo, A. Association between neighborhood deprivation and fruits and vegetables consumption and leisure-time physical activity: a cross-sectional multilevel analysis. *BMC Public Health*. 19. An, R., & Sturm, R. School and residential neighborhood food environment and diet among California youth. *Am J Prev Med*. 20. Anderson, A. S., Dewar, J., Marshall, D., Cummins, S., Taylor, M., Dawson, J., & Sparks, L. The development of a healthy eating indicator shopping basket tool (HEISB) for use in food access studies - identification of key food items. *Public Health Nutrition*. 21. Andress, L., & Fitch, C. Juggling the five dimensions of food access: Perceptions of rural low income residents. *Appetite*. 22. Andreyeva, T., Blumenthal, D. M., Schwartz, M. B., Long, M. W., & Brownell, K. D. Availability And Prices Of Foods Across Stores And Neighborhoods: The Case Of New Haven, Connecticut. *Health Affairs*. 23. Anguelovski, I. Healthy Food Stores, Greenlining and Food Gentrification: Contesting New Forms of Privilege, Displacement and Locally Unwanted Land Uses in Racially Mixed Neighborhoods. *International Journal of Urban and Regional Research*. 24. Apparicio, P., Cloutier, M. S., & Shearmur, R. The case of Montreal's missing food deserts: Evaluation of accessibility to food supermarkets. *International Journal of Health Geographics*. 25. Arcan, C., Neumark-Sztainer, D., Hannan, P., van den Berg, P., Story, M., & Larson, N. Parental eating behaviours, No measure of spatial exposure and adolescent intakes of fruits, vegetables and dairy foods: longitudinal findings from Project EAT. *Public Health Nutr*. 26. Ard, J. D., Perumean-Chaney, S., Desmond, R., Sutton, B., Cox, T. L., Butsch, W. S., . . . Baskin, M. L. Fruit and vegetable pricing by demographic factors in the Birmingham, Alabama, metropolitan area, 2004-2005. *Prev Chronic Dis*. 27. Auchincloss, A. H. Neighborhood Resources for Physical Activity and Healthy Foods and Incidence of Type 2 Diabetes Mellitus. *Archives of Internal Medicine*. 28. Auchincloss, A. H., Diez Roux, A. V., Brown, D. G., Erdmann, C. A., & Bertoni, A. G. Neighborhood resources for physical activity and healthy foods and their association with insulin resistance. *Epidemiology*. 29. Auchincloss, A. H., Diez Roux, A. V., Brown, D. G., Raghunathan, T. E., & Erdmann, C. A. Filling the gaps: Spatial interpolation of residential survey data in the estimation of neighborhood characteristics. *Epidemiology*. 30. Auchincloss, A. H., Diez Roux, A. V., Mujahid, M. S., Shen, M., Bertoni, A. G., & Carnethon, M. R. Neighborhood resources for physical activity and healthy foods and incidence of type 2 diabetes mellitus: the Multi-Ethnic study of Atherosclerosis. *Arch Intern Med*. 31. Auchincloss, A. H., Moore, K. A. B., Moore, L. V., & Diez Roux, A. V. Improving retrospective characterization of the food environment for a large region in the United States during a historic time period. *Health & Place*. 32. Auchincloss, A. H., Mujahid, M. S., Shen, M., Michos, E. D., Whitt-Glover, M. C., & Diez Roux, A. V. Neighborhood health-promoting resources and obesity risk (the multi-ethnic study of atherosclerosis). *Obesity (Silver Spring)*. 33. Auerbach, B. J., Katz, R., Tucker, K., Boyko, E. J., Drewnowski, A., Bertoni, A., . . . Young, B. A. Factors associated with maintenance of body mass index in the Jackson Heart Study: A prospective cohort study secondary analysis. *Preventive Medicine*. 34. Ayala, G. X., Rogers, M., Arredondo, E. M., Campbell, N. R., Baquero, B., Duerksen, S. C., & Elder, J. P. Away-from-home food intake and risk for obesity: examining the influence of context. *Obesity (Silver Spring)*. 35. Azeredo, C. M., de Rezende, L. F. M., Canella, D. S., Claro, R. M., Peres, M. F. T., Luiz, O. d. C., . . . Levy, R. B. Food environments in schools and in the immediate vicinity are associated with unhealthy food consumption among Brazilian adolescents. *Preventive Medicine*. 36. Azuma, A. M., Gilliland, S., Vallianatos, M., & Gottlieb, R. Food Access, Availability, and Affordability in 3 Los Angeles Communities, Project CAFE, 2004-2006. *Preventing Chronic Disease*. 37. Bader, M. D. M., Ailshire, J. A., Morenoff, J. D., & House, J. S. Measurement of the Local Food Environment: A Comparison of Existing Data Sources. *American Journal of Epidemiology*. 38. Bader, M. D. M., Purciel, M., Yousefzadeh, P., & Neckerman, K. M. Disparities in Neighborhood Food Environments: Implications of Measurement Strategies. *Economic Geography*. 39. Baek, J., Hirsch, J. A., Moore, K., Tabb, L. P., Barrientos-Gutierrez, T., Lisabeth, L. D., . . . Sáncheza, B. N. Statistical methods to study variation in associations between food store availability and body mass in the multi-ethnic study of atherosclerosis. *Epidemiology*. 40. Baek, J., Sanchez-Vaznaugh, E. V., & Sanchez, B. N. Hierarchical Distributed-Lag Models: Exploring Varying Geographic Scale and Magnitude in Associations Between the Built Environment and Health. *American Journal of Epidemiology*. 41. Bahn, R. A., & Abebe, G. K. Analysis of food retail patterns in urban, peri-urban and rural settings: A case study from Lebanon. *Applied Geography*. 42. Ball, K., Lamb, K. E., Costa, C., Cutumisu, N., Ellaway, A., Kamphuis, C. B., . . . Zenk, S. N. Neighbourhood socioeconomic disadvantage and fruit and vegetable consumption: a seven countries comparison. *Int J Behav Nutr Phys Act*. 43. Baranowski, T., Missaghian, M., Watson, K., Broadfoot, A., Cullen, K., Nicklas, T., . . . O'Donnell, S. Home fruit, juice, and vegetable pantry management and availability scales: a validation. *Appetite*. 44. Barnes, T. L., Bell, B. A., Freedman, D. A., Colabianchi, N., & Liese, A. D. Do people really know what food retailers exist in their neighborhood? Examining GIS-based and perceived presence of retail food outlets in an eight-county region of South Carolina. *Spat Spatiotemporal Epidemiol*. 45. Barnes, T. L., Colabianchi, N., Freedman, D. A., Bell, B. A., & Liese, A. D. Do GIS-derived measures of fast food retailers convey perceived fast food opportunities? Implications for food environment assessment. *Annals of Epidemiology*. 46. Barnes, T. L., Colabianchi, N., Hibbert, J. D., Porter, D. E., Lawson, A. B., & Liese, A. D. Scale effects in food environment research: Implications from assessing socioeconomic dimensions of supermarket accessibility in an eight-county region of South Carolina. *Applied Geography*. 47. Barnes, T. L., Freedman, D. A., Bell, B. A., Colabianchi, N., & Liese, A. D. Geographic measures of retail food outlets and perceived availability of healthy foods in neighbourhoods. *Public Health Nutrition*. 48. Barr, S. Using Mixed Methods to Describe a Spatially Dynamic Food Environment in Rural Dominican Republic. *Human Ecology*. 49. Barrientos-Gutierrez, T., Moore, K. A. B., Auchincloss, A. H., Mujahid, M. S., August, C., Sanchez, B. N., & Diez Roux, A. V. Neighborhood Physical Environment and Changes in Body Mass Index: Results From the Multi-Ethnic Study of Atherosclerosis. *Am J Epidemiol*. 50. Barrington, W. E., Beresford, S. A., Koepsell, T. D., Duncan, G. E., & Moudon, A. V. Worksite neighborhood and obesogenic behaviors: findings among employees in the Promoting Activity and Changes in Eating (PACE) trial. *Am J Prev Med*. 51. Barton, M., Kearney, J., & Stewart-Knox, B. J. Knowledge of food production methods informs attitudes toward food but not food choice in adults residing in socioeconomically deprived rural areas within the United Kingdom. *J Nutr Educ Behav*. 52. Battersby, J., & Crush, J. Africa's Urban Food Deserts. *Urban Forum*. 53. Battersby, J., & Peyton, S. The Geography of Supermarkets in Cape Town: Supermarket Expansion and Food Access. *Urban Forum*. 54. Berg, N., & Murdoch, J. Access to grocery stores in Dallas. *International Journal of Behavioural and Healthcare Research*. 55. Bertrand, L., Thérien, F., & Cloutier, M. S. Measuring and mapping disparities in access to fresh fruits and vegetables in Montréal. *Canadian Journal of Public Health*. 56. Bethlehem, J. R., Mackenbach, J. D., Ben-Rebah, M., Compernolle, S., Glonti, K., Bárdos, H., . . . Lakerveld, J. The SPOTLIGHT virtual audit tool: a valid and reliable tool to assess obesogenic characteristics of the built environment. *International Journal of Health Geographics*. 57. Black, C., Moon, G., & Baird, J. Dietary inequalities: What is the evidence for the effect of the neighbourhood food environment? *Health & Place*. 58. Black, C., Ntani, G., Inskip, H., Cooper, C., Cummins, S., Moon, G., & Baird, J. Measuring the healthfulness of food retail stores: variations by store type and neighbourhood deprivation. *International Journal of Behavioral Nutrition and Physical Activity*. 59. Black, C., Ntani, G., Kenny, R., Tinati, T., Jarman, M., Lawrence, W., . . . Baird, J. Variety and quality of healthy foods differ according to neighbourhood deprivation. *Health & Place*. 60. Black, J. L., & Day, M. Availability of Limited Service Food Outlets Surrounding Schools in British Columbia. *Canadian Journal of Public Health-Revue Canadienne De Sante Publique*. 61. Black, J. L., & Macinko, J. The Changing Distribution and Determinants of Obesity in the Neighborhoods of New York City, 2003–2007. *American Journal of Epidemiology*. 62. Blanck, H. M., Thompson, O. M., Nebeling, L., & Yaroch, A. L. Improving fruit and vegetable consumption: Use of farm-to-consumer venues among US adults. *Preventing Chronic Disease*. 63. Blitstein, J. L., Snider, J., & Evans, W. D. Perceptions of the food shopping environment are associated with greater consumption of fruits and vegetables. *Public Health Nutr*. 64. Block, D. What fills the gaps in food deserts? Mapping independent groceries, food stamp card utilization and chain fast-food restaurants in the Chicago area. *Appetite*. 65. Block, D., & Kouba, J. A comparison of the availability and affordability of a market basket in two communities in the Chicago area. *Public Health Nutrition*. 66. Block, J., Scribner, R., & Desalvo, K. Fast food, race/ethnicity, and incomeA geographic analysis. *American Journal of Preventive Medicine*. 67. Block, J. P., Christakis, N. A., O’Malley, A. J., & Subramanian, S. V. Proximity to Food Establishments and Body Mass Index in the Framingham Heart Study Offspring Cohort Over 30 Years. *American Journal of Epidemiology*. 68. Bodicoat, D. H., Carter, P., Comber, A., Edwardson, C., Gray, L. J., Hill, S., . . . Khunti, K. Is the number of fast-food outlets in the neighbourhood related to screen-detected type 2 diabetes mellitus and associated risk factors? *Public Health Nutr*. 69. Bodor, J. N., Hutchinson, P. L., & Rose, D. Car ownership and the association between fruit and vegetable availability and diet. *Prev Med*. 70. Bodor, J. N., Rice, J. C., Farley, T. A., Swalm, C. M., & Rose, D. The association between obesity and urban food environments. *J Urban Health*. 71. Bodor, J. N., Rice, J. C., Farley, T. A., Swalm, C. M., & Rose, D. Disparities in food access: Does aggregate availability of key foods from other stores offset the relative lack of supermarkets in African-American neighborhoods? *Preventive Medicine*. 72. Boehm, T. P., & Ihlanfeld, K. R. RESIDENTIAL MOBILITY AND NEIGHBORHOOD QUALITY. *Journal of Regional Science*. 73. Boonchoo, W., Takemi, Y., Hayashi, F., Koiwai, K., & Ogata, H. Dietary intake and weight status of urban Thai preadolescents in the context of food environment. *Preventive Medicine Reports*. 74. Boone-Heinonen, J., Diez-Roux, A. V., Goff, D. C., Loria, C. M., Kiefe, C. I., Popkin, B. M., & Gordon-Larsen, P. The neighborhood energy balance equation: does neighborhood food retail environment + physical activity environment = obesity? The CARDIA study. *PLoS One*. 75. Boone-Heinonen, J., & Gordon-Larsen, P. Obesogenic environments in youth: concepts and methods from a longitudinal national sample. *Am J Prev Med*. 76. Borkhoff, C. M., Saskin, R., Rabeneck, L., Baxter, N. N., Liu, Y., Tinmouth, J., & Paszat, L. F. Disparities in receipt of screening tests for cancer, diabetes and high cholesterol in Ontario, Canada: A population-based study using area-based methods. *Canadian Journal of Public Health*. 77. Boruff, B. J., Nathan, A., & Nijenstein, S. Using GPS technology to (re)-examine operational definitions of 'neighbourhood' in place-based health research. *Int J Health Geogr*. 78. Boucher, B. A., Manafò, E., Boddy, M. R., Roblin, L., & Truscott, R. The Ontario food and nutrition strategy: Identifying indicators of food access and food literacy for early monitoring of the food environment. *Health Promotion and Chronic Disease Prevention in Canada*. 79. Boutelle, K. N., Fulkerson, J. A., Neumark-Sztainer, D., Story, M., & French, S. A. Fast food for family meals: relationships with parent and adolescent food intake, home food availability and weight status. *Public Health Nutr*. 80. Bovell-Benjamin, A. C., Hathorn, C. S., Ibrahim, S., Gichuhi, P. N., & Bromfield, E. M. Healthy food choices and physical activity opportunities in two contrasting Alabama cities. *Health & Place*. 81. Bower, K. M., Thorpe, R. J., Rohde, C., & Gaskin, D. J. The intersection of neighborhood racial segregation, poverty, and urbanicity and its impact on food store availability in the United States. *Preventive Medicine*. 82. Breyer, B., & Voss-Andreae, A. Food mirages: Geographic and economic barriers to healthful food access in Portland, Oregon. *Health & Place*. 83. Bridle-Fitzpatrick, S. Food deserts or food swamps?: A mixed-methods study of local food environments in a Mexican city. *Soc Sci Med*. 84. Brimblecombe, J., Mackerras, D., & Clifford, P. Does the store-turnover method still provide a useful guide to food intakes in Aboriginal communities? *Australian and New Zealand Journal of Public Health*. 85. Brinkley, C., Raj, S., & Horst, M. Culturing food deserts: Recognizing the power of community-based solutions. *Built Environment*. 86. Brumback, B. A., Cai, Z., & Dailey, A. B. Methods of estimating or accounting for neighborhood associations with health using complex survey data. *Am J Epidemiol*. 87. Brumback, B. A., Dailey, A. B., & Zheng, H. W. Adjusting for confounding by neighborhood using a proportional odds model and complex survey data. *Am J Epidemiol*. 88. Bryant, M., & Stevens, J. Measurement of Food Availability in the Home. *Nutrition Reviews*. 89. Burga, H. F., & Stoscheck, C. Does the Minnesota food access planning guide address food justice and equity? A content analysis of policy language. *Built Environment*. 90. Burgoine, T. Collecting accurate secondary foodscape data. A reflection on the trials and tribulations. *Appetite*. 91. Burgoine, T., Alvanides, S., & Lake, A. A. Assessing the obesogenic environment of North East England. *Health & Place*. 92. Burgoine, T., Alvanides, S., & Lake, A. A. Creating 'obesogenic realities'; do our methodological choices make a difference when measuring the food environment? *International Journal of Health Geographics*. 93. Burgoine, T., Gallis, J. A., L. Penney, T., Monsivais, P., & Benjamin Neelon, S. E. Association between distance to nearest supermarket and provision of fruits and vegetables in English nurseries. *Health & Place*. 94. Burgoine, T., Mackenbach, J. D., Lakerveld, J., Forouhi, N. G., Griffin, S. J., Brage, S., . . . Monsivais, P. Interplay of socioeconomic status and supermarket distance is associated with excess obesity risk: A UK cross-sectional study. *International Journal of Environmental Research and Public Health*. 95. Burnett, K., Skinner, K., Hay, T., LeBlanc, J., & Chambers, L. Retail food environments, shopping experiences, first nations and the provincial Norths. *Health Promotion and Chronic Disease Prevention in Canada*. 96. Burns, C., Bentley, R., Thornton, L., & Kavanagh, A. Reduced food access due to a lack of money, inability to lift and lack of access to a car for food shopping: a multilevel study in Melbourne, Victoria. *Public Health Nutr*. 97. Burns, C. M., & Inglis, A. D. Measuring food access in Melbourne: Access to healthy and fast foods by car, bus and foot in an urban municipality in Melbourne. *Health and Place*. 98. Buro, B., Gold, A., Contreras, D., Keim, A. L., Mobley, A. R., Oscarson, R., . . . Smathers, C. An Ecological Approach to Exploring Rural Food Access and Active Living for Families With Preschoolers. *J Nutr Educ Behav*. 99. Buscemi, S., Nicolucci, A., Lucisano, G., Galvano, F., Grosso, G., Belmonte, S., . . . Rini, G. B. Habitual fish intake and clinically silent carotid atherosclerosis. *Nutr J*. 100. Bustillos, B., Sharkey, J. R., Anding, J., & McIntosh, A. Availability of More Healthful Food Alternatives in Traditional, Convenience, and Nontraditional Types of Food Stores in Two Rural Texas Counties. *Journal of the American Dietetic Association*. 101. Byker Shanks, C., Ahmed, S., Smith, T., Houghtaling, B., Jenkins, M., Margetts, M., . . . Stephens, L. Availability, Price, and Quality of Fruits and Vegetables in 12 Rural Montana Counties, 2014. *Prev Chronic Dis*. 102. Byrd-Bredbenner, C., & Abbot, J. M. Differences in food supplies of U.S. households with and without overweight individuals. *Appetite*. 103. Caldwell, E. M., Miller Kobayashi, M., DuBow, W. M., & Wytinck, S. M. Perceived access to fruits and vegetables associated with increased consumption. *Public Health Nutrition*. 104. Cameron, A. J., Thornton, L. E., McNaughton, S. A., & Crawford, D. Variation in supermarket exposure to energy-dense snack foods by socio-economic position. *Public Health Nutrition*. 105. Campbell, K. J., Crawford, D. A., Salmon, J., Carver, A., Garnett, S. P., & Baur, L. A. Associations between the No measure of spatial exposure and obesity-promoting eating behaviors in adolescence. *Obesity (Silver Spring)*. 106. Campbell-Arvai, V. Food-related environmental beliefs and behaviours among university undergraduates a mixed-methods study. *International Journal of Sustainability in Higher Education*. 107. Cannuscio, C. C., Hillier, A., Karpyn, A., & Glanz, K. The social dynamics of healthy food shopping and store choice in an urban environment. *Soc Sci Med*. 108. Cannuscio, C. C., Tappe, K., Hillier, A., Buttenheim, A., Karpyn, A., & Glanz, K. Urban food environments and residents' shopping behaviors. *Am J Prev Med*. 109. Cappon, D. Criteria for healthy urban environments. *Canadian Journal of Public Health*. 110. Caraher, M., Lloyd, S., Mansfield, M., Alp, C., Brewster, Z., & Gresham, J. Secondary school pupils' food choices around schools in a London borough: Fast food and walls of crisps. *Appetite*. 111. Carroll, S. J., Paquet, C., Howard, N. J., Coffee, N. T., Adams, R. J., Taylor, A. W., . . . Daniel, M. Local descriptive body weight and dietary norms, food availability, and 10-year change in glycosylated haemoglobin in an Australian population-based biomedical cohort. *BMC Public Health*. 112. Casagrande, S. S., Franco, M., Gittelsohn, J., Zonderman, A. B., Evans, M. K., Fanelli Kuczmarski, M., & Gary-Webb, T. L. Healthy food availability and the association with BMI in Baltimore, Maryland. *Public Health Nutr*. 113. Casey, R., Chaix, B., Weber, C., Schweitzer, B., Charreire, H., Salze, P., . . . Simon, C. Spatial accessibility to physical activity facilities and to food outlets and overweight in French youth. *International Journal of Obesity*. 114. Caspi, C. E., Pelletier, J. E., Harnack, L. J., Erickson, D. J., Lenk, K., & Laska, M. N. Pricing of staple foods at supermarkets versus small food stores. *International Journal of Environmental Research and Public Health*. 115. Cavanaugh, E., Green, S., Mallya, G., Tierney, A., Brensinger, C., & Glanz, K. Changes in food and beverage environments after an urban corner store intervention. *Preventive Medicine*. 116. Cavanaugh, E., Mallya, G., Brensinger, C., Tierney, A., & Glanz, K. Nutrition environments in corner stores in Philadelphia. *Preventive Medicine*. 117. Cerin, E. Statistical approaches to testing the relationships of the built environment with resident-level physical activity behavior and health outcomes in cross-sectional studies with cluster sampling. *Journal of Planning Literature*. 118. Cerin, E., Frank, L. D., Sallis, J. F., Saelens, B. E., Conway, T. L., Chapman, J. E., & Glanz, K. From neighborhood design and food options to residents' weight status. *Appetite*. 119. Cetateanu, A., & Jones, A. How can GPS technology help us better understand exposure to the food environment? A systematic review. *SSM - Population Health*. 120. Chaix, B., Kestens, Y., Duncan, D. T., Brondeel, R., Meline, J., El Aarbaoui, T., . . . Merlo, J. A GPS-Based Methodology to Analyze Environment-Health Associations at the Trip Level: Case-Crossover Analyses of Built Environments and Walking. *Am J Epidemiol*. 121. Chaix, B., Méline, J., Duncan, S., Merrien, C., Karusisi, N., Perchoux, C., . . . Kestens, Y. GPS tracking in neighborhood and health studies: A step forward for environmental exposure assessment, A step backward for causal inference? *Health and Place*. 122. Chaix, B., Merlo, J., Subramanian, S. V., Lynch, J., & Chauvin, P. Comparison of a spatial perspective with the multilevel analytical approach in neighborhood studies: the case of mental and behavioral disorders due to psychoactive substance use in Malmo, Sweden, 2001. *Am J Epidemiol*. 123. Chambers, L. W., Haynes, R. B., Pickering, R., McKibbon, A., Walker-Dilks, C. J., Panton, L., & Goldblatt, E. New approaches to addressing information needs in local Public Health agencies. *Canadian Journal of Public Health*. 124. Chaparro, M. P., Harrison, G. G., Wang, M. C., Seto, E. Y., & Pebley, A. R. The unhealthy food environment does not modify the association between obesity and participation in the Supplemental Nutrition Assistance Program (SNAP) in Los Angeles County. *BMC Public Health*. 125. Charlton, E. L., Kähkönen, L. A., Sacks, G., & Cameron, A. J. Supermarkets and unhealthy food marketing: An international comparison of the content of supermarket catalogues/circulars. *Preventive Medicine*. 126. Charreire, H., Casey, R., Salze, P., Simon, C., Chaix, B., Banos, A., . . . Oppert, J. M. Measuring the food environment using geographical information systems: a methodological review. *Public Health Nutrition*. 127. Charreire, H., Mackenbach, J. D., Ouasti, M., Lakerveld, J., Compernolle, S., Ben-Rebah, M., . . . Oppert, J. M. Using remote sensing to define environmental characteristics related to physical activity and dietary behaviours: A systematic review (the SPOTLIGHT project). *Health and Place*. 128. Charreire, H., Weber, C., Chaix, B., Salze, P., Casey, R., Banos, A., . . . Oppert, J. M. Identifying built environmental patterns using cluster analysis and GIS: relationships with walking, cycling and body mass index in French adults. *Int J Behav Nutr Phys Act*. 129. Chaudhari, L. S., Begay, R. C., & Schulz, L. O. Fifteen years of change in the food environment in a rural Mexican community: the Maycoba project. *Rural and Remote Health*. 130. Cheadle, A., Psaty, B. M., Curry, S., Wagner, E., Diehr, P., Koepsell, T., & Kristal, A. CAN MEASURES OF THE GROCERY STORE ENVIRONMENT BE USED TO TRACK COMMUNITY-LEVEL DIETARY-CHANGES. *Preventive Medicine*. 131. Cheadle, A., Psaty, B. M., Curry, S., Wagner, E., Diehr, P., Koepsell, T., & Kristal, A. Community-level comparisons between the grocery store environment and individual dietary practices. *Preventive Medicine*. 132. Cheadle, A., Psaty, B. M., Diehr, P., Koepsell, T., Wagner, E., Curry, S., & Kristal, A. Evaluating Community-Based Nutrition-Programs: Comparing Grocery Store and Individual-Level Survey Measures of Program Impact. *Preventive Medicine*. 133. Chen, D., Jaenicke, E. C., & Volpe, R. J. Food Environments and Obesity: Household Diet Expenditure Versus Food Deserts. *Am J Public Health*. 134. Chen, H. J., & Wang, Y. The changing food outlet distributions and local contextual factors in the United States. *BMC Public Health*. 135. Chen, X. Take the edge off: A hybrid geographic food access measure. *Applied Geography*. 136. Chen, X., & Clark, J. Interactive three-dimensional geovisualization of space–time access to food. *Applied Geography*. 137. Chi, S.-H., Grigsby-Toussaint, D. S., Bradford, N., & Choi, J. Can Geographically Weighted Regression improve our contextual understanding of obesity in the US? Findings from the USDA Food Atlas. *Applied Geography*. 138. Chiang, P. H., Wahlqvist, M. L., Lee, M. S., Huang, L. Y., Chen, H. H., & Huang, S. T. Fast-food outlets and walkability in school neighbourhoods predict fatness in boys and height in girls: a Taiwanese population study. *Public Health Nutr*. 139. Chodur, G. M., Shen, Y., Kodish, S., Oddo, V. M., Antiporta, D. A., Jock, B., & Jones-Smith, J. C. Food Environments around American Indian Reservations: A Mixed Methods Study. *PLoS One*. 140. Chor, D., Cardoso, L. O., Nobre, A. A., Griep, R. H., Fonseca, M. D. J. M., Giatti, L., . . . Santos, S. M. Association between perceived neighbourhood characteristics, physical activity and diet quality: results of the Brazilian Longitudinal Study of Adult Health (ELSA-Brasil). *BMC Public Health*. 141. Chrisinger, B. Evaluating healthy corner stores: A survey of assessment tools used in the San Francisco Bay Area, 2016. *Preventing Chronic Disease*. 142. Chrisinger, B. A Mixed-Method Assessment of a New Supermarket in a Food Desert: Contributions to Everyday Life and Health. *Journal of Urban Health-Bulletin of the New York Academy of Medicine*. 143. Christian, H., Giles-Corti, B., Knuiman, M., Timperio, A., & Foster, S. The influence of the built environment, social environment and health behaviors on body mass index. results from RESIDE. *Prev Med*. 144. Christine, P. J., Moore, K., Crawford, N. D., Barrientos-Gutierrez, T., Sanchez, B. N., Seeman, T., & Diez Roux, A. V. Exposure to Neighborhood Foreclosures and Changes in Cardiometabolic Health: Results From MESA. *Am J Epidemiol*. 145. Clarke, M. A., Haire-Joshu, D. L., Schwarz, C. D., Tabak, R. G., & Joshu, C. E. Influence of home and school environments on specific dietary behaviors among postpartum, high-risk teens, 27 States, 2007-2009. *Prev Chronic Dis*. 146. Clarke, P., Ailshire, J., Melendez, R., Bader, M., & Morenoff, J. Using Google Earth to conduct a neighborhood audit: Reliability of a virtual audit instrument. *Health & Place*. 147. Cobb, L. K., Appel, L. J., Franco, M., Jones-Smith, J. C., Nur, A., & Anderson, C. A. M. The relationship of the local food environment with obesity: A systematic review of methods, study quality, and results. *Obesity*. 148. Cohen, D. A., & Babey, S. H. Contextual influences on eating behaviours: Heuristic processing and dietary choices. *Obesity Reviews*. 149. Cohen, D. A., Hunter, G., Williamson, S., & Dubowitz, T. Are Food Deserts Also Play Deserts? *J Urban Health*. 150. Cohen, D. A., Schoeff, D., Farley, T. A., Bluthenthal, R., Scribner, R., & Overton, A. Reliability of a store observation tool in measuring availability of alcohol and selected foods. *Journal of Urban Health-Bulletin of the New York Academy of Medicine*. 151. Compernolle, S., Oppert, J. M., Mackenbach, J. D., Lakerveld, J., Charreire, H., Glonti, K., . . . De Bourdeaudhuij, I. Mediating role of energy-balance related behaviors in the association of neighborhood socio-economic status and residential area density with BMI: The SPOTLIGHT study. *Prev Med*. 152. Congdon, P. Variations in obesity rates between US counties: Impacts of activity access, food environments, and settlement patterns. *International Journal of Environmental Research and Public Health*. 153. Coogan, P. F., White, L. F., Adler, T. J., Hathaway, K. M., Palmer, J. R., & Rosenberg, L. Prospective study of urban form and physical activity in the Black Women's Health Study. *Am J Epidemiol*. 154. Coogan, P. F., White, L. F., Evans, S. R., Adler, T. J., Hathaway, K. M., Palmer, J. R., & Rosenberg, L. Longitudinal assessment of urban form and weight gain in African-American women. *Am J Prev Med*. 155. Cook, L. T., O'Reilly, G. A., DeRosa, C. J., Rohrbach, L. A., & Spruijt-Metz, D. Association between home availability and vegetable consumption in youth: a review. *Public Health Nutr*. 156. Cowan, J. A., & Devine, C. M. Diet and body composition outcomes of an environmental and educational intervention among men in treatment for substance addiction. *J Nutr Educ Behav*. 157. Cowburn, G., Matthews, A., Doherty, A., Hamilton, A., Kelly, P., Williams, J., . . . Nelson, M. Exploring the opportunities for food and drink purchasing and consumption by teenagers during their journeys between home and school: a feasibility study using a novel method. *Public Health Nutr*. 158. Crawford, T. W., Pitts, S. B. J., McGuirt, J. T., Keyserling, T. C., & Ammerman, A. S. Conceptualizing and comparing neighborhood and activity space measures for food environment research. *Health & Place*. 159. Creel, J. S., Sharkey, J. R., McIntosh, A., Anding, J., & Huber, J. C. Availability of healthier options in traditional and nontraditional rural fast-food outlets. *BMC Public Health*. 160. Crush, J., & Caesar, M. City Without Choice: Urban Food Insecurity in Msunduzi, South Africa. *Urban Forum*. 161. Crush, J., & McCordic, C. The Hungry Cities Food Purchases Matrix: Household Food Sourcing and Food System Interaction. *Urban Forum*. 162. Cubbin, C., Jun, J., Margerison-Zilko, C., Welch, N., Sherman, J., McCray, T., & Parmenter, B. Social inequalities in neighborhood conditions: spatial relationships between sociodemographic and food environments in Alameda County, California. *Journal of Maps*. 163. Cubbin, C., & Winkleby, M. A. Protective and harmful effects of neighborhood-level deprivation on individual-level health knowledge, behavior changes, and risk of coronary heart disease. *Am J Epidemiol*. 164. Cummins, S., Findlay, A., Petticrew, M., & Sparks, L. Healthy Cities: The Impact of Food Retail-led Regeneration on Food Access, Choice and Retail Structure. *Built Environment*. 165. Cummins, S., Flint, E., & Matthews, S. A. New neighborhood grocery store increased awareness of food access but did not alter dietary habits or obesity. *Health Aff (Millwood)*. 166. Cummins, S., McKay, L., & Macintyre, S. McDonald’s Restaurants and Neighborhood Deprivation in Scotland and England. *American Journal of Preventive Medicine*. 167. Cummins, S., Smith, D. M., Aitken, Z., Dawson, J., Marshall, D., Sparks, L., & Anderson, A. S. Neighbourhood deprivation and the price and availability of fruit and vegetables in Scotland. *Journal of Human Nutrition and Dietetics*. 168. Dai, D. J., & Wang, F. H. Geographic disparities in accessibility to food stores in southwest Mississippi. *Environment and Planning B-Planning & Design*. 169. Dake, F. A. A., Thompson, A. L., Ng, S. W., Agyei-Mensah, S., & Codjoe, S. N. A. The Local Food Environment and Body Mass Index among the Urban Poor in Accra, Ghana. *Journal of Urban Health*. 170. Dammann, K. W., & Smith, C. Food-related environmental, behavioral, and personal factors associated with body mass index among urban, low-income African-American, American Indian, and Caucasian women. *Am J Health Promot*. 171. Dammann, K. W., & Smith, C. Race, homelessness, and other environmental factors associated with the food-purchasing behavior of low-income women. *J Am Diet Assoc*. 172. Dannefer, R., Williams, D. A., Baronberg, S., & Silver, L. Healthy bodegas: Increasing and promoting healthy foods at corner stores in New York City. *American Journal of Public Health*. 173. Day, P. L., & Pearce, J. Obesity-promoting food environments and the spatial clustering of food outlets around schools. *Am J Prev Med*. 174. de Castro, J. M., King, G. A., Duarte-Gardea, M., Gonzalez-Ayala, S., & Kooshian, C. H. Overweight and obese humans overeat away from home. *Appetite*. 175. de Graaf, C., Kramer, F. M., Meiselman, H. L., Lesher, L. L., Baker-Fulco, C., Hirsch, E. S., & Warber, J. Food acceptability in field studies with US army men and women: relationship with food intake and food choice after repeated exposures. *Appetite*. 176. Dean, M., Raats, M. M., Grunert, K. G., & Lumbers, M. Factors influencing eating a varied diet in old age. *Public Health Nutr*. 177. Dean, W. R., Sharkey, J. R., & St. John, J. Alternative components of the retail food environment. Healthy food availability in South Texas pulgas. *Appetite*. 178. DeBono, N. L., Ross, N. A., & Berrang-Ford, L. Does the Food Stamp Program cause obesity? A realist review and a call for place-based research. *Health & Place*. 179. Dehghan, M., Akhtar-Danesh, N., & Merchant, A. T. Factors associated with fruit and vegetable consumption among adults. *Journal of Human Nutrition and Dietetics*. 180. Del Canto, S., Engler-Stringer, R., & Muhajarine, N. Characterizing Saskatoon's food environment: A neighbourhood-level analysis of in-store fruit and vegetable access. *Canadian Journal of Urban Research*. 181. Delva, J., O'Malley, P. M., & Johnston, L. D. Availability of more-healthy and less-healthy food choices in American schools: a national study of grade, racial/ethnic, and socioeconomic differences. *Am J Prev Med*. 182. Dennisuk, L. A., Coutinho, A. J., Suratkar, S., Surkan, P. J., Christiansen, K., Riley, M., . . . Gittelsohn, J. Food expenditures and food purchasing among low-income, urban, African-American youth. *Am J Prev Med*. 183. Díez, J., Conde, P., Sandin, M., Urtasun, M., López, R., Carrero, J. L., . . . Franco, M. Understanding the local food environment: A participatory photovoice project in a low-income area in Madrid, Spain. *Health & Place*. 184. Diez, J., Valiente, R., Ramos, C., Garcia, R., Gittelsohn, J., & Franco, M. The mismatch between observational measures and residents' perspectives on the retail food environment: a mixed-methods approach in the Heart Healthy Hoods study. *Public Health Nutrition*. 185. Dixon, J., Omwega, A. M., Friel, S., Burns, C., Donati, K., & Carlisle, R. The health equity dimensions of urban food systems. *Journal of Urban Health*. 186. Do, D. P., & Finch, B. K. The link between neighborhood poverty and health: context or composition? *Am J Epidemiol*. 187. Donald, B. Food retail and access after the crash: rethinking the food desert problem. *Journal of Economic Geography*. 188. Donkin, A. J. M., Dowler, E. A., Stevenson, S. J., & Turner, S. A. Mapping access to food in a deprived area: the development of price and availability indices. *Public Health Nutrition*. 189. Drewnowski, A. Obesity and the food environment - Dietary energy density and diet costs. *American Journal of Preventive Medicine*. 190. Drewnowski, A., Aggarwal, A., Hurvitz, P. M., Monsivais, P., & Moudon, A. V. Obesity and supermarket access: proximity or price? *Am J Public Health*. 191. Drewnowski, A., Moudon, A. V., Jiao, J., Aggarwal, A., Charreire, H., & Chaix, B. Food environment and socioeconomic status influence obesity rates in Seattle and in Paris. *Int J Obes (Lond)*. 192. Dubowitz, T., Acevedo-Garcia, D., Salkeld, J., Cristina Lindsay, A., Subramanian, S. V., & Peterson, K. E. Lifecourse, immigrant status and acculturation in food purchasing and preparation among low-income mothers. *Public Health Nutrition*. 193. Dubowitz, T., Ghosh-Dastidar, M., Eibner, C., Slaughter, M. E., Fernandes, M., Whitsel, E. A., . . . Escarce, J. J. The Women's Health Initiative: The food environment, neighborhood socioeconomic status, BMI, and blood pressure. *Obesity (Silver Spring)*. 194. Dubowitz, T., Ncube, C., Leuschner, K., & Tharp-Gilliam, S. A natural experiment opportunity in two low-income urban food desert communities: research design, community engagement methods, and baseline results. *Health Educ Behav*. 195. Duran, A. C., Roux, A. V. D., Latorre, M., & Jaime, P. C. Neighborhood socioeconomic characteristics and differences in the availability of healthy food stores and restaurants in Sao Paulo, Brazil. *Health & Place*. 196. Earland, J., Campbell, J., & Srivastava, A. Dietary habits and health status of African-Caribbean adults. *J Hum Nutr Diet*. 197. Eckert, J., & Shetty, S. Food systems, planning and quantifying access: Using GIS to plan for food retail. *Applied Geography*. 198. Eckert, J., & Vojnovic, I. Fast food landscapes: Exploring restaurant choice and travel behavior for residents living in lower eastside Detroit neighborhoods. *Applied Geography*. 199. Economos, C. D., Hatfield, D. P., King, A. C., Ayala, G. X., & Pentz, M. A. Food and Physical Activity Environments: An Energy Balance Approach for Research and Practice. *American Journal of Preventive Medicine*. 200. Edmonds, J., Baranowski, T., Baranowski, J., Cullen, K. W., & Myres, D. Ecological and socioeconomic correlates of fruit, juice, and vegetable consumption among African-American boys. *Prev Med*. 201. Eisenberg, Y., Vanderbom, K. A., & Vasudevan, V. Does the built environment moderate the relationship between having a disability and lower levels of physical activity? A systematic review. *Preventive Medicine*. 202. Elbel, B., Taksler, G. B., Mijanovich, T., Abrams, C. B., & Dixon, L. B. Promotion of healthy eating through public policy: A controlled experiment. *American Journal of Preventive Medicine*. 203. Ellen, I. G., Mijanovich, T., & Dillman, K. N. Neighborhood effects on health: Exploring the links and assessing the evidence. *Journal of Urban Affairs*. 204. Emery, C. F., Olson, K. L., Lee, V. S., Habash, D. L., Nasar, J. L., & Bodine, A. Home environment and psychosocial predictors of obesity status among community-residing men and women. *Int J Obes (Lond)*. 205. Emond, J. A., Madanat, H. N., & Ayala, G. X. Do Latino and non-Latino grocery stores differ in the availability and affordability of healthy food items in a low-income, metropolitan region? *Public Health Nutrition*. 206. Erinosho, T. O., Oh, A. Y., Moser, R. P., Davis, K. L., Nebeling, L. C., & Yaroch, A. L. Association between perceived food environment and self-efficacy for fruit and vegetable consumption among US adults, 2007. *Prev Chronic Dis*. 207. Escaron, A. L., Meinen, A. M., Nitzke, S. A., & Martinez-Donate, A. P. Supermarket and grocery store-based interventions to promote healthful food choices and eating practices: A systematic review. *Preventing Chronic Disease*. 208. Fan, J. X., Hanson, H. A., Zick, C. D., Brown, B. B., Kowaleski-Jones, L., & Smith, K. R. Geographic scale matters in detecting the relationship between neighbourhood food environments and obesity risk: an analysis of driver license records in Salt Lake County, Utah. *BMJ Open*. 209. Farley, T. A., Rice, J., Bodor, J. N., Cohen, D. A., Bluthenthal, R. N., & Rose, D. Measuring the food environment: Shelf space of fruits, vegetables, and snack foods in stores. *Journal of Urban Health*. 210. Feathers, A., Aycinena, A. C., Lovasi, G. S., Rundle, A., Gaffney, A. O., Richardson, J., . . . Greenlee, H. Food environments are relevant to recruitment and adherence in dietary modification trials. *Nutr Res*. 211. Feng, J., Glass, T. A., Curriero, F. C., Stewart, W. F., & Schwartz, B. S. The built environment and obesity: A systematic review of the epidemiologic evidence. *Health & Place*. 212. Ferrer-Garcia, M., Pla-Sanjuanelo, J., Dakanalis, A., Vilalta-Abella, F., Riva, G., Fernandez-Aranda, F., . . . Gutiérrez-Maldonado, J. Eating behavior style predicts craving and anxiety experienced in food-related virtual environments by patients with eating disorders and healthy controls. *Appetite*. 213. Feunekes, G. I., Gortemaker, I. A., Willems, A. A., Lion, R., & van den Kommer, M. Front-of-pack nutrition labelling: testing effectiveness of different nutrition labelling formats front-of-pack in four European countries. *Appetite*. 214. Feunekes, G. I. J., de Graaf, C., Meyboom, S., & van Staveren, W. A. Food Choice and Fat Intake of Adolescents and Adults: Associations of Intakes within Social Networks. *Preventive Medicine*. 215. Findholt, N. E., Izumi, B. T., Shannon, J., & Nguyen, T. Food-related practices and beliefs of rural US elementary and middle school teachers. *Rural Remote Health*. 216. Firdaus, G. Built Environment and Health Outcomes: Identification of Contextual Risk Factors for Mental Well-being of Older Adults. *Ageing International*. 217. Fleischhacker, S. E., Evenson, K. R., Rodriguez, D. A., & Ammerman, A. S. A systematic review of fast food access studies. *Obesity Reviews*. 218. Fleischhacker, S. E., Evenson, K. R., Sharkey, J., Pitts, S. B. J., & Rodriguez, D. A. Validity of Secondary Retail Food Outlet Data: A Systematic Review. *American Journal of Preventive Medicine*. 219. Foltz, J. L., Harris, D. M., & Blanck, H. M. Support among U.S. adults for local and state policies to increase fruit and vegetable access. *Am J Prev Med*. 220. Ford, P. B., & Dzewaltowski, D. A. Limited supermarket availability is not associated with obesity risk among participants in the Kansas WIC Program. *Obesity (Silver Spring)*. 221. Forwood, S. E., Ahem, A. L., Marteau, T. M., & Jebb, S. A. Offering within-category food swaps to reduce energy density of food purchases: a study using an experimental online supermarket. *International Journal of Behavioral Nutrition and Physical Activity*. 222. Franco, M., Diez Roux, A. V., Glass, T. A., Caballero, B., & Brancati, F. L. Neighborhood Characteristics and Availability of Healthy Foods in Baltimore. *American Journal of Preventive Medicine*. 223. Franco, M., Ordunez, P., Caballero, B., Tapia Granados, J. A., Lazo, M., Bernal, J. L., . . . Cooper, R. S. Impact of energy intake, physical activity, and population-wide weight loss on cardiovascular disease and diabetes mortality in Cuba, 1980-2005. *Am J Epidemiol*. 224. Frank, L. D., Saelens, B. E., Chapman, J., Sallis, J. F., Kerr, J., Glanz, K., . . . Cain, K. L. Objective Assessment of Obesogenic Environments in Youth: Geographic Information System Methods and Spatial Findings from the Neighborhood Impact on Kids Study. *American Journal of Preventive Medicine*. 225. Frank, L. D., Schmid, T. L., Sallis, J. F., Chapman, J., & Saelens, B. E. Linking objectively measured physical activity with objectively measured urban form: findings from SMARTRAQ. *Am J Prev Med*. 226. Frankenfeld, C. L., Leslie, T. F., & Makara, M. A. Diabetes, obesity, and recommended fruit and vegetable consumption in relation to food environment sub-types: A cross-sectional analysis of Behavioral Risk Factor Surveillance System, United States Census, and food establishment data. *BMC Public Health*. 227. Franzen, L., & Smith, C. Food system access, shopping behavior, and influences on purchasing groceries in adult Hmong living in Minnesota. *Am J Health Promot*. 228. Fraser, L. K., Edwards, K. L., Cade, J. E., & Clarke, G. P. Fast food, other food choices and body mass index in teenagers in the United Kingdom (ALSPAC): a structural equation modelling approach. *Int J Obes (Lond)*. 229. Fraser, L. K., Edwards, K. L., Tominitz, M., Clarke, G. P., & Hill, A. J. Food outlet availability, deprivation and obesity in a multi-ethnic sample of pregnant women in Bradford, UK. *Soc Sci Med*. 230. Freedman, D. A., & Bell, B. A. Access to healthful foods among an urban food insecure population: Perceptions versus reality. *Journal of Urban Health*. 231. French, S. A., Epstein, L. H., Jeffery, R. W., Blundell, J. E., & Wardle, J. Eating behavior dimensions. Associations with energy intake and body weight. A review. *Appetite*. 232. French, S. A., & Stables, G. Environmental interventions to promote vegetable and fruit consumption among youth in school settings. *Prev Med*. 233. Fuller, D., Engler-Stringer, R., & Muhajarine, N. Examining food purchasing patterns from sales data at a full-service grocery store intervention in a former food desert. *Preventive Medicine Reports*. 234. Furst, T., Connors, M., Bisogni, C. A., Sobal, J., & Falk, L. W. Food choice: a conceptual model of the process. *Appetite*. 235. Galvez, M. P., Morland, K., Raines, C., Kobil, J., Siskind, J., Godbold, J., & Brenner, B. Race and food store availability in an inner-city neighbourhood. *Public Health Nutrition*. 236. Gamba, R. J., Schuchter, J., Rutt, C., & Seto, E. Y. W. Measuring the Food Environment and its Effects on Obesity in the United States: A Systematic Review of Methods and Results. *Journal of Community Health*. 237. Garden, F. L., & Jalaludin, B. B. Impact of urban sprawl on overweight, obesity, and physical activity in Sydney, Australia. *J Urban Health*. 238. Gasevic, D., Vukmirovich, I., Yusuf, S., Teo, K., Chow, C., Dagenais, G., & Lear, S. A. A direct assessment of 'obesogenic' built environments: Challenges and recommendations. *Journal of Environmental and Public Health*. 239. Gauvin, L., Riva, M., Barnett, T., Richard, L., Craig, C. L., Spivock, M., . . . Gagne, S. Association between neighborhood active living potential and walking. *Am J Epidemiol*. 240. Geaney, F., Kelly, C., Di Marrazzo, J. S., Harrington, J. M., Fitzgerald, A. P., Greiner, B. A., & Perry, I. J. The effect of complex workplace dietary interventions on employees' dietary intakes, nutrition knowledge and health status: a cluster controlled trial. *Preventive Medicine*. 241. Geliebter, A., Ang, I., Bernales-Korins, M., Hernandez, D., Ochner, C. N., Ungredda, T., . . . Kolbe, L. Supermarket discounts of low-energy density foods: Effects on purchasing, food intake, and body weight. *Obesity*. 242. Ghirardelli, A., Quinn, V., & Foerster, S. B. Using Geographic Information Systems and Local Food Store Data in California's Low-Income Neighborhoods to Inform Community Initiatives and Resources. *American Journal of Public Health*. 243. Ghosh-Dastidar, B., Cohen, D., Hunter, G., Zenk, S. N., Huang, C., Beckman, R., & Dubowitz, T. Distance to store, food prices, and obesity in urban food deserts. *Am J Prev Med*. 244. Ghosh-Dastidar, M., Hunter, G., Collins, R. L., Zenk, S. N., Cummins, S., Beckman, R., . . . Dubowitz, T. Does opening a supermarket in a food desert change the food environment? *Health & Place*. 245. Gibson, D. M. The neighborhood food environment and adult weight status: estimates from longitudinal data. *Am J Public Health*. 246. Giles-Corti, B., Bull, F., Knuiman, M., McCormack, G., Van Niel, K., Timperio, A., . . . Boruff, B. The influence of urban design on neighbourhood walking following residential relocation: longitudinal results from the RESIDE study. *Soc Sci Med*. 247. Giles-Corti, B., Timperio, A., Cutt, H., Pikora, T. J., Bull, F. C. L., Knuiman, M., . . . Shilton, T. Development of a reliable measure of walking within and outside the local neighborhood: RESIDE's Neighborhood Physical Activity Questionnaire. *Preventive Medicine*. 248. Giskes, K., van Lenthe, F., Avendano-Pabon, M., & Brug, J. A systematic review of environmental factors and obesogenic dietary intakes among adults: are we getting closer to understanding obesogenic environments? *Obes Rev*. 249. Giskes, K., van Lenthe, F. J., Kamphuis, C. B. M., Huisman, M., Brug, J., & Mackenbach, J. P. Household and food shopping environments: do they play a role in socioeconomic inequalities in fruit and vegetable consumption? A multilevel study among Dutch adults. *Journal of Epidemiology & Community Health*. 250. Gittelsohn, J., Kim, E. M., He, S., & Pardilla, M. A food store-based environmental intervention is associated with reduced BMI and improved psychosocial factors and food-related behaviors on the Navajo nation. *J Nutr*. 251. Gittelsohn, J., Laska, M. N., Karpyn, A., Klingler, K., & Ayala, G. X. Lessons Learned From Small Store Programs to Increase Healthy Food Access. *American Journal of Health Behavior*. 252. Gittelsohn, J., Rowan, M., & Gadhoke, P. Interventions in small food stores to change the food environment, improve diet, and reduce risk of chronic disease. *Preventing Chronic Disease*. 253. Gittelsohn, J., & Sharma, S. Physical, Consumer, and Social Aspects of Measuring the Food Environment Among Diverse Low-Income Populations. *American Journal of Preventive Medicine*. 254. Gittelsohn, J., Song, H. J., Suratkar, S., Kumar, M. B., Henry, E. G., Sharma, S., . . . Anliker, J. A. An urban food store intervention positively affects food-related psychosocial variables and food behaviors. *Health Educ Behav*. 255. Giurgescu, C., Zenk, S. N., Templin, T. N., Engeland, C. G., Dancy, B. L., Park, C. G., . . . Misra, D. P. The Impact of Neighborhood Environment, Social Support, and Avoidance Coping on Depressive Symptoms of Pregnant African-American Women. *Womens Health Issues*. 256. Glanz, K., Johnson, L., Yaroch, A. L., Phillips, M., Ayala, G. X., & Davis, E. L. Measures of Retail Food Store Environments and Sales: Review and Implications for Healthy Eating Initiatives. *Journal of Nutrition Education and Behavior*. 257. Glanz, K., Sallis, J. F., & Saelens, B. E. Advances in Physical Activity and Nutrition Environment Assessment Tools and Applications: Recommendations. *American Journal of Preventive Medicine*. 258. Glanz, K., Sallis, J. F., Saelens, B. E., & Frank, L. D. Nutrition environment measures survey in stores (NEMS-S) - Development and evaluation. *American Journal of Preventive Medicine*. 259. Gomez-Lopez, I. N., Clarke, P., Hill, A. B., Romero, D. M., Goodspeed, R., Berrocal, V. J., . . . Veinot, T. C. Using Social Media to Identify Sources of Healthy Food in Urban Neighborhoods. *Journal of Urban Health*. 260. Gordon, C., Purciel-Hill, M., Ghai, N. R., Kaufman, L., Graham, R., & Van Wye, G. Measuring food deserts in New York City's low-income neighborhoods. *Health & Place*. 261. Gould, A. C., Apparicio, P., & Cloutier, M. S. Classifying Neighbourhoods by Level of Access to Stores Selling Fresh Fruit and Vegetables and Groceries: Identifying Problematic Areas in the City of Gatineau, Quebec. *Canadian Journal of Public Health-Revue Canadienne De Sante Publique*. 262. Graham, D. J., Pelletier, J. E., Neumark-Sztainer, D., Lust, K., & Laska, M. N. Perceived social-ecological factors associated with fruit and vegetable purchasing, preparation, and consumption among young adults. *J Acad Nutr Diet*. 263. Grant, E., Gearry, R. B., Wilson, R., Pearson, J., & Skidmore, P. M. L. Home availability of fruit and vegetables and obesogenic foods as an indicator of nutrient intake in 50 year olds from Canterbury, New Zealand. *Asia Pac J Clin Nutr*. 264. Grier, S. A., & Kumanyika, S. K. The context for choice: Health implications of targeted food and beverage marketing to African Americans. *American Journal of Public Health*. 265. Grigsby-Toussaint, D. S., Zenk, S. N., Odoms-Young, A., Ruggiero, L., & Moise, I. Availability of Commonly Consumed and Culturally Specific Fruits and Vegetables in African-American and Latino Neighborhoods. *Journal of the American Dietetic Association*. 266. Grills, C., Villanueva, S., Subica, A. M., & Douglas, J. A. Communities Creating Healthy Environments: Improving access to healthy foods and safe places to play in communities of color. *Preventive Medicine*. 267. Grindal, T., Wilde, P., Schwartz, G., Klerman, J., Bartlett, S., & Berman, D. Does food retail access moderate the impact of fruit and vegetable incentives for SNAP participants? Evidence from western Massachusetts. *Food Policy*. 268. Gustafson, A., Christian, J. W., Lewis, S., Moore, K., & Jilcott, S. Food venue choice, consumer food environment, but not food venue availability within daily travel patterns are associated with dietary intake among adults, Lexington Kentucky 2011. *Nutr J*. 269. Gustafson, A. A., Sharkey, J., Samuel-Hodge, C. D., Jones-Smith, J., Folds, M. C., Cai, J. W., & Ammerman, A. S. Perceived and objective measures of the food store environment and the association with weight and diet among low-income women in North Carolina. *Public Health Nutrition*. 270. Guy, C. M., & David, G. Measuring physical access to 'healthy foods' in areas of social deprivation: a case study in Cardiff. *International Journal of Consumer Studies*. 271. Hallett Iv, L. F., & McDermott, D. Quantifying the extent and cost of food deserts in Lawrence, Kansas, USA. *Applied Geography*. 272. Hamilton, S., Mhurchu, C. N., & Priest, P. Food and nutrient availability in New Zealand: an analysis of supermarket sales data. *Public Health Nutr*. 273. Hampl, J. S., Anderson, J. V., & Mullis, R. Position of the American Dietetic Association: The Role of Dietetics Professionals in Health Promotion and Disease Prevention. *Journal of the American Dietetic Association*. 274. Hanson, N. I., Neumark-Sztainer, D., Eisenberg, M. E., Story, M., & Wall, M. Associations between parental report of the No measure of spatial exposure and adolescent intakes of fruits, vegetables and dairy foods. *Public Health Nutr*. 275. Harris, D. E., Aboueissa, A. M., Jacobus, M. V., Dharod, J., & Walter, K. Mapping food stores &amp; people at risk for food insecurity in Lewiston, Maine. *Journal of Extension*. 276. Haynes-Maslow, L., & Leone, L. A. Examining the relationship between the food environment and adult diabetes prevalence by county economic and racial composition: An ecological study. *BMC Public Health*. 277. Haynes-Maslow, L., Parsons, S. E., Wheeler, S. B., & Leone, L. A. A qualitative study of perceived barriers to fruit and vegetable consumption among low-income populations, North Carolina, 2011. *Prev Chronic Dis*. 278. He, M., Tucker, P., Gilliland, J., Irwin, J. D., Larsen, K., & Hess, P. The influence of local food environments on adolescents' food purchasing behaviors. *Int J Environ Res Public Health*. 279. He, M., Tucker, P., Irwin, J. D., Gilliland, J., Larsen, K., & Hess, P. Obesogenic neighbourhoods: the impact of neighbourhood restaurants and convenience stores on adolescents' food consumption behaviours. *Public Health Nutr*. 280. Hearst, M. O., Pasch, K. E., & Laska, M. N. Urban v. suburban perceptions of the neighbourhood food environment as correlates of adolescent food purchasing. *Public Health Nutr*. 281. Heinrich, K. M., Lee, R. E., Regan, G. R., Reese-Smith, J. Y., Howard, H. H., Haddock, C. K., . . . Ahluwalia, J. S. How does the built environment relate to body mass index and obesity prevalence among public housing residents? *Am J Health Promot*. 282. Helbich, M., Schadenberg, B., Hagenauer, J., & Poelman, M. Food deserts? Healthy food access in Amsterdam. *Applied Geography*. 283. Hemphill, E., Raine, K., Spence, J. C., & Smoyer-Tomic, K. E. Exploring obesogenic food environments in Edmonton, Canada: The association between socioeconomic factors and fast-food outlet access. *American Journal of Health Promotion*. 284. Henderson, C., Diez Roux, A. V., Jacobs, D. R., Jr., Kiefe, C. I., West, D., & Williams, D. R. Neighbourhood characteristics, individual level socioeconomic factors, and depressive symptoms in young adults: the CARDIA study. *J Epidemiol Community Health*. 285. Henry, H., Reicks, M., Smith, C., Reimer, K., Atwell, J., & Thomas, R. Identification of factors affecting purchasing and preparation of fruit and vegetables by stage of change for low-income African American mothers using the think-aloud method. *J Am Diet Assoc*. 286. Herforth, A., & Ahmed, S. The food environment, its effects on dietary consumption, and potential for measurement within agriculture-nutrition interventions. *Food Security*. 287. Héroux, M., Iannotti, R. J., Currie, D., Pickett, W., & Janssen, I. The food retail environment in school neighborhoods and its relation to lunchtime eating behaviors in youth from three countries. *Health & Place*. 288. Hewitt, G., Draper, A., Ismail, S., & Patterson, S. Improving food provision in a Guyanese home for the elderly: a participatory approach. *Public Health Nutr*. 289. Hill, J. L., Chau, C., Luebbering, C. R., Kolivras, K. K., & Zoellner, J. Does availability of physical activity and food outlets differ by race and income? Findings from an enumeration study in a health disparate region. *International Journal of Behavioral Nutrition and Physical Activity*. 290. Hilmers, A., Hilmers, D. C., & Dave, J. Neighborhood Disparities in Access to Healthy Foods and Their Effects on Environmental Justice. *American Journal of Public Health*. 291. Hirai, H., Kondo, N., Sasaki, R., Iwamuro, S., Masuno, K., Ohtsuka, R., . . . Sakata, K. Distance to retail stores and risk of being homebound among older adults in a city severely affected by the 2011 Great East Japan Earthquake. *Age Ageing*. 292. Hirsch, J. A., Diez Roux, A. V., Moore, K. A., Evenson, K. R., & Rodriguez, D. A. Change in walking and body mass index following residential relocation: the multi-ethnic study of atherosclerosis. *Am J Public Health*. 293. Hirsch, J. A., Green, G. F., Peterson, M., Rodriguez, D. A., & Gordon-Larsen, P. Neighborhood sociodemographics and change in built infrastructure. *Journal of Urbanism*. 294. Hirsch, J. A., & Hillier, A. Exploring the role of the food environment on food shopping patterns in Philadelphia, PA, USA: a semiquantitative comparison of two matched neighborhood groups. *Int J Environ Res Public Health*. 295. Hirsch, J. A., Moore, K. A., Clarke, P. J., Rodriguez, D. A., Evenson, K. R., Brines, S. J., . . . Roux, A. V. D. Changes in the built environment and changes in the amount of walking over time: Longitudinal results from the Multi-Ethnic study of Atherosclerosis. *American Journal of Epidemiology*. 296. Hollands, S., Campbell, M. K., Gilliland, J., & Sarma, S. Association between neighbourhood fast-food and full-service restaurant density and body mass index: A cross-sectional study of Canadian adults. *Canadian Journal of Public Health-Revue Canadienne De Sante Publique*. 297. Hollands, S., Campbell, M. K., Gilliland, J., & Sarma, S. A spatial analysis of the association between restaurant density and body mass index in Canadian adults. *Prev Med*. 298. Holmes, B., Dick, K., & Nelson, M. A comparison of four dietary assessment methods in materially deprived households in England. *Public Health Nutr*. 299. Hong, T., & Farley, T. A. Urban residents' priorities for neighborhood features. A survey of New Orleans residents after Hurricane Katrina. *Am J Prev Med*. 300. Horner, M. W., & Wood, B. S. Capturing individuals' food environments using flexible space-time accessibility measures. *Applied Geography*. 301. Horowitz, C. R., Colson, K. A., Hebert, P. L., & Lancaster, K. Barriers to Buying Healthy Foods for People With Diabetes: Evidence of Environmental Disparities. *American Journal of Public Health*. 302. Horwath, C. C., & Worsley, A. ASSESSMENT OF THE VALIDITY OF A FOOD FREQUENCY QUESTIONNAIRE AS A MEASURE OF FOOD USE BY COMPARISON WITH DIRECT OBSERVATION OF DOMESTIC FOOD STORES. *American Journal of Epidemiology*. 303. Hosler, A. S., & Dharssi, A. Identifying Retail Food Stores to Evaluate the Food Environment. *American Journal of Preventive Medicine*. 304. Hosler, A. S., & Kammer, J. R. Trends of fruit and vegetable availability in neighbourhoods in Albany, NY, USA, 2003-2012. *Public Health Nutrition*. 305. Hosler, A. S., Michaels, I. H., & Buckenmeyer, E. M. Food Shopping Venues, Neighborhood Food Environment, and Body Mass Index Among Guyanese, Black, and White Adults in an Urban Community in the US. *J Nutr Educ Behav*. 306. Hosler, A. S., Rajulu, D. T., Fredrick, B. L., & Ronsani, A. E. Assessing retail fruit and vegetable availability in urban and rural underserved communities. *Preventing Chronic Disease*. 307. Howell, N. M., Priest, T. B., & Hayes, J. G. NEIGHBORHOOD CHARACTERISTICS, CHAIN GROCERY STORES, AND FOOD: A Research Note. *Journal of Urban Affairs*. 308. Hsieh, S., Klassen, A. C., Curriero, F. C., Caulfield, L. E., Cheskin, L. J., Davis, J. N., . . . Spruijt-Metz, D. Fast-food restaurants, park access, and insulin resistance among Hispanic youth. *Am J Prev Med*. 309. Hsu, W. C., Bath, P. A., Large, S., & Williams, S. The association of geographical location and neighbourhood deprivation with older people's use of NHS Direct: a population-based study. *Age Ageing*. 310. Hu, H. H., Cho, J., Huang, G., Wen, F., Choi, S., Shih, M., & Lightstone, A. S. Neighborhood environment and health behavior in Los Angeles area. *Transport Policy*. 311. Hubley, T. A. Assessing the proximity of healthy food options and food deserts in a rural area in Maine. *Applied Geography*. 312. Hurvitz, P. M., & Moudon, A. V. Home versus nonhome neighborhood: quantifying differences in exposure to the built environment. *Am J Prev Med*. 313. Hutchinson, P. L., Nicholas Bodor, J., Swalm, C. M., Rice, J. C., & Rose, D. Neighbourhood food environments and obesity in southeast Louisiana. *Health Place*. 314. Ibem, E. O. Accessibility of Services and Facilities for Residents in Public Housing in Urban Areas of Ogun State, Nigeria. *Urban Forum*. 315. Inagami, S., Cohen, D. A., Finch, B. K., & Asch, S. M. You are where you shop: grocery store locations, weight, and neighborhoods. *Am J Prev Med*. 316. Inglis, V., Ball, K., & Crawford, D. Socioeconomic variations in women's diets: what is the role of perceptions of the local food environment? *J Epidemiol Community Health*. 317. Jackson, L., Langille, L., Lyons, R., Hughes, J., Martin, D., & Winstanley, V. Does moving from a high-poverty to lower-poverty neighborhood improve mental health? A realist review of 'Moving to Opportunity'. *Health Place*. 318. Jalbert-Arsenault, É., Robitaille, É., & Paquette, M. C. Development, reliability and use of a food environment assessment tool in supermarkets of four neighbourhoods in Montréal, Canada. *Health Promotion and Chronic Disease Prevention in Canada*. 319. James, P., Arcaya, M. C., Parker, D. M., Tucker-Seeley, R. D., & Subramanian, S. V. Do minority and poor neighborhoods have higher access to fast-food restaurants in the United States? *Health & Place*. 320. James, R. C., & Mustard, C. A. Geographic location of commercial plasma donation clinics in the United States, 1980-1995. *American Journal of Public Health*. 321. Jaskiewicz, L., Block, D., & Chavez, N. Finding Food Deserts: A Comparison of Methods Measuring Spatial Access to Food Stores. *Health Promotion Practice*. 322. Jaskiewicz, L., Dombrowski, R. D., Drummond, H. M., Barnett, G. M., Mason, M., & Welter, W. Partnering with community institutions to increase access to healthful foods across municipalities. *Preventing Chronic Disease*. 323. Jennings, A., Cassidy, A., Winters, T., Barnes, S., Lipp, A., Holland, R., & Welch, A. Positive effect of a targeted intervention to improve access and availability of fruit and vegetables in an area of deprivation. *Health Place*. 324. Jiao, J., Moudon, A. V., Ulmer, J., Hurvitz, P. M., & Drewnowski, A. How to identify food deserts: Measuring physical and economic access to supermarkets in King County, Washington. *American Journal of Public Health*. 325. Jilcott Pitts, S. B., Wu, Q., Demarest, C. L., Dixon, C. E., Dortche, C. J., Bullock, S. L., . . . Ammerman, A. S. Farmers' market shopping and dietary behaviours among Supplemental Nutrition Assistance Program participants. *Public Health Nutr*. 326. Jilcott Pitts, S. B., Wu, Q., Truesdale, K. P., Laska, M. N., Grinchak, T., McGuirt, J. T., . . . Ammerman, A. S. Baseline assessment of a healthy corner store initiative: Associations between food store environments, shopping patterns, customer purchases, and dietary intake in eastern North Carolina. *International Journal of Environmental Research and Public Health*. 327. Jilcott, S. B., Keyserling, T., Crawford, T., McGuirt, J. T., & Ammerman, A. S. Examining Associations among Obesity and Per Capita Farmers' Markets, Grocery Stores/Supermarkets, and Supercenters in US Counties. *Journal of the American Dietetic Association*. 328. Jilcott, S. B., Laraia, B. A., Evenson, K. R., & Ammerman, A. S. Perceptions of the community food environment and related influences on food choice among midlife women residing in rural and urban areas: a qualitative analysis. *Women Health*. 329. Jilcott, S. B., Liu, H., Moore, J. B., Bethel, J. W., Wilson, J., & Ammerman, A. S. Commute times, food retail gaps, and body mass index in North Carolina counties. *Prev Chronic Dis*. 330. Jilcott, S. B., McGuirt, J. T., Imai, S., & Evenson, K. R. Measuring the retail food environment in rural and Urban North carolina counties. *Journal of Public Health Management and Practice*. 331. Jilcott, S. B., Wade, S., McGuirt, J. T., Wu, Q., Lazorick, S., & Moore, J. B. The association between the food environment and weight status among eastern North Carolina youth. *Public Health Nutr*. 332. Johnson, D. B., Quinn, E., Sitaker, M., Ammerman, A., Byker, C., Dean, W., . . . Sharkey, J. Developing an agenda for research about policies to improve access to healthy foods in rural communities: A concept mapping study. *BMC Public Health*. 333. Jokela, M. Are neighborhood health associations causal? A 10-year prospective cohort study with repeated measurements. *Am J Epidemiol*. 334. Jones, A. Residential instability and obesity over time: the role of the social and built environment. *Health Place*. 335. Joost, S., Duruz, S., Marques-Vidal, P., Bochud, M., Stringhini, S., Paccaud, F., . . . Guessous, I. Persistent spatial clusters of high body mass index in a Swiss urban population as revealed by the 5-year geocolaus longitudinal study. *BMJ Open*. 336. Kaiser, P., Auchincloss, A. H., Moore, K., Sánchez, B. N., Berrocal, V., Allen, N., & Roux, A. V. D. Associations of neighborhood socioeconomic and racial/ethnic characteristics with changes in survey-based neighborhood quality, 2000–2011. *Health & Place*. 337. Kamphuis, C. B., van Lenthe, F. J., Giskes, K., Brug, J., & Mackenbach, J. P. Perceived environmental determinants of physical activity and fruit and vegetable consumption among high and low socioeconomic groups in the Netherlands. *Health Place*. 338. Keita, A. D., Casazza, K., Thomas, O., & Fernandez, J. R. Neighborhood Perceptions Affect Dietary Behaviors and Diet Quality. *Journal of Nutrition Education and Behavior*. 339. Kelly, B., Flood, V. M., & Yeatman, H. Measuring local food environments: An overview of available methods and measures. *Health & Place*. 340. Kern, D. M., Auchincloss, A. H., Robinson, L. F., Stehr, M. F., & Pham-Kanter, G. Healthy and Unhealthy Food Prices across Neighborhoods and Their Association with Neighborhood Socioeconomic Status and Proportion Black/Hispanic. *Journal of Urban Health*. 341. Kersten, E., Laraia, B., Kelly, M., Adler, N., & Yen, I. H. Small food stores and availability of nutritious foods: a comparison of database and in-store measures, Northern California, 2009. *Prev Chronic Dis*. 342. Kestens, Y., & Daniel, M. Social inequalities in food exposure around schools in an urban area. *Am J Prev Med*. 343. Kim, K., Hong, S. A., Yun, S. H., Ryou, H. J., Lee, S. S., & Kim, M. K. The effect of a healthy school tuck shop program on the access of students to healthy foods. *Nutrition Research and Practice*. 344. Kim, T. H., Lee, E.-K., & Han, E. Food away from home and body mass outcomes: Taking heterogeneity into account enhances quality of results. *Nutrition*. 345. Kimenju, S. C., Rischke, R., Klasen, S., & Qaim, M. Do supermarkets contribute to the obesity pandemic in developing countries? *Public Health Nutr*. 346. King, A. C., Glanz, K., & Patrick, K. Technologies to measure and modify physical activity and eating environments. *American Journal of Preventive Medicine*. 347. King, T. L., Bentley, R. J., Thornton, L. E., & Kavanagh, A. M. Using kernel density estimation to understand the influence of neighbourhood destinations on BMI. *BMJ Open*. 348. Kirby, R. S., Delmelle, E., & Eberth, J. M. Advances in spatial epidemiology and geographic information systems. *Annals of Epidemiology*. 349. Kirkpatrick, S. I., Reedy, J., Butler, E. N., Dodd, K. W., Subar, A. F., Thompson, F. E., & McKinnon, R. A. Dietary Assessment in Food Environment Research: A Systematic Review. *American Journal of Preventive Medicine*. 350. Kirkpatrick, S. I., & Tarasuk, V. Assessing the relevance of neighbourhood characteristics to the household food security of low-income Toronto families. *Public Health Nutr*. 351. Knuiman, M. W., Christian, H. E., Divitini, M. L., Foster, S. A., Bull, F. C., Badland, H. M., & Giles-Corti, B. A longitudinal analysis of the influence of the neighborhood built environment on walking for transportation: the RESIDE study. *Am J Epidemiol*. 352. Krishnan, S., Cozier, Y. C., Rosenberg, L., & Palmer, J. R. Socioeconomic status and incidence of type 2 diabetes: results from the Black Women's Health Study. *Am J Epidemiol*. 353. Kristal, A. R., Goldenhar, L., Muldoon, J., & Morton, R. F. Evaluation of a supermarket intervention to increase consumption of fruits and vegetables. *American Journal of Health Promotion*. 354. Krukowski, R. A., Sparks, C., DiCarlo, M., McSweeney, J., & West, D. S. There's more to food store choice than proximity: a questionnaire development study. *BMC Public Health*. 355. Krukowski, R. A., West, D. S., Harvey-Berino, J., & Prewitt, T. E. Neighborhood Impact on Healthy Food Availability and Pricing in Food Stores. *Journal of Community Health*. 356. Kubik, M. Y., Lytle, L. A., Hannan, P. J., Perry, C. L., & Story, M. The association of the school food environment with dietary behaviors of young adolescents. *Am J Public Health*. 357. Kumar, G., Jim-Martin, S., Piltch, E., Onufrak, S., McNeil, C., Adams, L., . . . Curley, L. Healthful Nutrition of Foods in Navajo Nation Stores: Availability and Pricing. *American Journal of Health Promotion*. 358. Kwate, N. O., & Loh, J. M. Separate and unequal: the influence of neighborhood and school characteristics on spatial proximity between fast food and schools. *Prev Med*. 359. Kwate, N. O. A., & Loh, J. M. Fast food and liquor store density, co-tenancy, and turnover: Vice store operations in Chicago, 1995-2008. *Applied Geography*. 360. Kwate, N. O. A., Yau, C. Y., Loh, J. M., & Williams, D. Inequality in obesigenic environments: Fast food density in New York City. *Health and Place*. 361. Kwate, N. O. A., Yau, C.-Y., Loh, J.-M., & Williams, D. Inequality in obesigenic environments: Fast food density in New York City. *Health & Place*. 362. Lake, A., & Townshend, T. Obesogenic environments: Exploring the built and food environments. *Journal of The Royal Society for the Promotion of Health*. 363. Lamb, K. E., Thornton, L. E., Cerin, E., & Ball, K. Statistical Approaches Used to Assess the Equity of Access to Food Outlets: A Systematic Review. *Aims Public Health*. 364. Lamb, K. E., Thornton, L. E., Olstad, D. L., Cerin, E., & Ball, K. Associations between major chain fast-food outlet availability and change in body mass index: A longitudinal observational study of women from Victoria, Australia. *BMJ Open*. 365. Lamichhane, A. P., Mayer-Davis, E. J., Puett, R., Bottai, M., Porter, D. E., & Liese, A. D. Associations of built food environment with dietary intake among youth with diabetes. *J Nutr Educ Behav*. 366. Lamichhane, A. P., Warren, J., Puett, R., Porter, D. E., Bottai, M., Mayer-Davis, E. J., & Liese, A. D. Spatial patterning of supermarkets and fast food outlets with respect to neighborhood characteristics. *Health & Place*. 367. Lamichhane, A. P., Warren, J. L., Peterson, M., Rummo, P., & Gordon-Larsen, P. Spatial-Temporal Modeling of Neighborhood Sociodemographic Characteristics and Food Stores. *American Journal of Epidemiology*. 368. Lang, I. A., Llewellyn, D. J., Langa, K. M., Wallace, R. B., & Melzer, D. Neighbourhood deprivation and incident mobility disability in older adults. *Age Ageing*. 369. Laraia, B. A., Downing, J. M., Zhang, Y. T., Dow, W. H., Kelly, M., Blanchard, S. D., . . . Karter, A. J. Food Environment and Weight Change: Does Residential Mobility Matter?: The Diabetes Study of Northern California (DISTANCE). *Am J Epidemiol*. 370. Larsen, K., & Gilliland, J. A farmers' market in a food desert: Evaluating impacts on the price and availability of healthy food. *Health and Place*. 371. Larsen, K., & Gilliland, J. Mapping the evolution of 'food deserts' in a Canadian city: Supermarket accessibility in London, Ontario, 1961-2005. *International Journal of Health Geographics*. 372. Larsen, K., & Merlo, J. Appropriate assessment of neighborhood effects on individual health: integrating random and fixed effects in multilevel logistic regression. *Am J Epidemiol*. 373. Larson, N. I., Neumark-Sztainer, D. R., Harnack, L. J., Wall, M. M., Story, M. T., & Eisenberg, M. E. Fruit and Vegetable Intake Correlates During the Transition to Young Adulthood. *American Journal of Preventive Medicine*. 374. Larson, N. I., Story, M. T., & Nelson, M. C. Neighborhood Environments Disparities in Access to Healthy Foods in the US. *American Journal of Preventive Medicine*. 375. Laska, M. N., Borradaile, K. E., Tester, J., Foster, G. D., & Gittelsohn, J. Healthy food availability in small urban food stores: a comparison of four US cities. *Public Health Nutrition*. 376. Laska, M. N., Graham, D. J., Moe, S. G., & Van Riper, D. Young adult eating and food-purchasing patterns food store location and residential proximity. *Am J Prev Med*. 377. Laska, M. N., Hearst, M. O., Forsyth, A., Pasch, K. E., & Lytle, L. Neighbourhood food environments: are they associated with adolescent dietary intake, food purchases and weight status? *Public Health Nutr*. 378. Lassen, A. D., Thorsen, A. V., Sommer, H. M., Fagt, S., Trolle, E., Biltoft-Jensen, A., & Tetens, I. Improving the diet of employees at blue-collar worksites: results from the 'Food at Work' intervention study. *Public Health Nutr*. 379. Laxer, R. E., & Janssen, I. The proportion of excessive fast-food consumption attributable to the neighbourhood food environment among youth living within 1 km of their school. *Appl Physiol Nutr Metab*. 380. Leal, C., Bean, K., Thomas, F., & Chaix, B. Multicollinearity in associations between multiple environmental features and body weight and abdominal fat: Using matching techniques to assess whether the associations are separable. *American Journal of Epidemiology*. 381. Lebel, A., Noreau, D., Tremblay, L., Oberlé, C., Girard-Gadreau, M., Duguay, M., & Block, J. P. Identifying rural food deserts: Methodological considerations for food environment interventions. *Canadian Journal of Public Health*. 382. Lee, A. J., Darcy, A. M., Leonard, D., Groos, A. D., Stubbs, C. O., Lowson, S. K., . . . Riley, M. D. Food availability, cost disparity and improvement in relation to accessibility and remoteness in Queensland. *Australian and New Zealand Journal of Public Health*. 383. Lee, A. J., Kane, S., Ramsey, R., Good, E., & Dick, M. Testing the price and affordability of healthy and current (unhealthy) diets and the potential impacts of policy change in Australia. *BMC Public Health*. 384. Lee, C., Ory, M. G., Yoon, J., & Forjuoh, S. N. Neighborhood walking among overweight and obese adults: age variations in barriers and motivators. *J Community Health*. 385. Lee, I. M., Ewing, R., & Sesso, H. D. The built environment and physical activity levels: the Harvard Alumni Health Study. *Am J Prev Med*. 386. Lee, L. M., & Thacker, S. B. Public health surveillance and knowing about health in the context of growing sources of health data. *American Journal of Preventive Medicine*. 387. Leonard, T., Ayers, C., Das, S. R., Neeland, I. J., & Powell-Wiley, T. M. Do neighborhoods matter differently for movers and non-movers? Analysis of weight gain in the longitudinal Dallas Heart Study. *Health and Place*. 388. Leonard, T., Shuval, K., de Oliveira, A., Skinner, C. S., Eckel, C., & Murdoch, J. C. Health behavior and behavioral economics: economic preferences and physical activity stages of change in a low-income African-American community. *Am J Health Promot*. 389. Leone, A. F., Rigby, S., Betterley, C., Park, S., Kurtz, H., Johnson, M. A., & Lee, J. S. Store Type and Demographic Influence on the Availability and Price of Healthful Foods, Leon County, Florida, 2008. *Preventing Chronic Disease*. 390. Leslie, T. F., Frankenfeld, C. L., & Makara, M. A. The spatial food environment of the DC metropolitan area: Clustering, co-location, and categorical differentiation. *Applied Geography*. 391. Leung, C. W., Laraia, B. A., Kelly, M., Nickleach, D., Adler, N. E., Kushi, L. H., & Yen, I. H. The Influence of Neighborhood Food Stores on Change in Young Girls' Body Mass Index. *American Journal of Preventive Medicine*. 392. Levasseur, M., Généreux, M., Desroches, J., Carrier, A., Lacasse, F., Chabot, É., . . . Vanasse, A. How to find lessons from the public health literature: Example of a scoping study protocol on the neighborhood environment. *International Journal of Preventive Medicine*. 393. Lewis, L. B., Sloane, D. C., Nascimento, L. M., Diamant, A. L., Guinyard, J. J., Yancey, A. K., & Flynn, G. African Americans’ Access to Healthy Food Options in South Los Angeles Restaurants. *American Journal of Public Health*. 394. Li, F., Harmer, P., Cardinal, B. J., Bosworth, M., & Johnson-Shelton, D. Obesity and the built environment: does the density of neighborhood fast-food outlets matter? *Am J Health Promot*. 395. Li, F., Harmer, P., Cardinal, B. J., Bosworth, M., Johnson-Shelton, D., Moore, J. M., . . . Vongjaturapat, N. Built environment and 1-year change in weight and waist circumference in middle-aged and older adults: Portland Neighborhood Environment and Health Study. *Am J Epidemiol*. 396. Li, F. Z., Harmer, P., Cardinal, B. J., Bosworth, M., & Johnson-Shelton, D. Obesity and the Built Environment: Does the Density of Neighborhood Fast-Food Outlets Matter? *American Journal of Health Promotion*. 397. Li, K. Y., Cromley, E. K., Fox, A. M., & Horowitz, C. R. Evaluation of the placement of mobile fruit and vegetable vendors to alleviate food deserts in New York city. *Preventing Chronic Disease*. 398. Liao, C. X., Tan, Y. Y., Wu, C. Q., Wang, S. F., Yu, C. Q., Cao, W. H., . . . Li, L. M. City Level of Income and Urbanization and Availability of Food Stores and Food Service Places in China. *PLoS One*. 399. Liese, A. D., Barnes, T. L., Lamichhane, A. P., Hibbert, J. D., Colabianchi, N., & Lawson, A. B. Characterizing the Food Retail Environment: Impact of Count, Type, and Geospatial Error in 2 Secondary Data Sources. *Journal of Nutrition Education and Behavior*. 400. Liese, A. D., Weis, K. E., Pluto, D., Smith, E., & Lawson, A. Food store types, availability, and cost of foods in a rural environment. *Journal of the American Dietetic Association*. 401. Lindsay, S., Lambert, J., Penn, T., Hedges, S., Ortwine, K., Mei, A., . . . Wooten, W. J. Monetary matched incentives to encourage the purchase of fresh fruits and vegetables at farmers markets in underserved communities. *Prev Chronic Dis*. 402. Lisabeth, L. D., Sánchez, B. N., Escobar, J., Hughes, R., Meurer, W. J., Zuniga, B., . . . Morgenstern, L. B. The food environment in an urban Mexican American community. *Health & Place*. 403. Litt, J. S., Soobader, M. J., Turbin, M. S., Hale, J. W., Buchenau, M., & Marshall, J. A. The influence of social involvement, neighborhood aesthetics, and community garden participation on fruit and vegetable consumption. *Am J Public Health*. 404. Liu, E., Stephenson, T., Houlihan, J., & Gustafson, A. Marketing strategies to encourage rural residents of high-obesity counties to buy fruits and vegetables in grocery stores. *Preventing Chronic Disease*. 405. Liu, J. L., Han, B., & Cohen, D. A. Beyond Neighborhood Food Environments: Distance Traveled to Food Establishments in 5 US Cities, 2009-2011. *Prev Chronic Dis*. 406. Locher, J. L., Ritchie, C. S., Roth, D. L., Sen, B., Vickers, K. S., & Vailas, L. I. Food choice among homebound older adults: motivations and perceived barriers. *J Nutr Health Aging*. 407. Longacre, M. R., Drake, K. M., Titus, L. J., Peterson, K. E., Beach, M. L., Langeloh, G., . . . Dalton, M. A. School food reduces household income disparities in adolescents' frequency of fruit and vegetable intake. *Prev Med*. 408. Longacre, M. R., Primack, B. A., Owens, P. M., Gibson, L., Beauregard, S., Mackenzie, T. A., & Dalton, M. A. Public Directory Data Sources Do Not Accurately Characterize the Food Environment in Two Predominantly Rural States. *Journal of the American Dietetic Association*. 409. Lovasi, G. S., Bader, M. D., Quinn, J., Neckerman, K., Weiss, C., & Rundle, A. Body mass index, safety hazards, and neighborhood attractiveness. *Am J Prev Med*. 410. Lowery, B., Sloane, D., Payán, D., Illum, J., & Lewis, L. Do Farmers' Markets Increase Access to Healthy Foods for All Communities? Comparing Markets in 24 Neighborhoods in Los Angeles. *Journal of the American Planning Association*. 411. Luan, H., Minaker, L. M., & Law, J. Do marginalized neighbourhoods have less healthy retail food environments? An analysis using Bayesian spatial latent factor and hurdle models. *International Journal of Health Geographics*. 412. Lucan, S. C., Maroko, A. R., Bumol, J., Varona, M., Torrens, L., & Schechter, C. B. Mobile food vendors in urban neighborhoods—Implications for diet and diet-related health by weather and season. *Health & Place*. 413. Lucan, S. C., & Mitra, N. Perceptions of the food environment are associated with fast-food (not fruit-and-vegetable) consumption: findings from multi-level models. *International Journal of Public Health*. 414. Lucumí, D. I., Sarmiento, O. L., Forero, R., Gomez, L. F., & Espinosa, G. Community intervention to promote consumption of fruits and vegetables, smokefree homes, and physical activity among home caregivers in Bogotá, Colombia. *Preventing Chronic Disease*. 415. Lundeen, E. A., VanFrank, B. K., Jackson, S. L., Harmon, B., Uncangco, A., Luces, P., . . . Park, S. Availability and Promotion of Healthful Foods in Stores and Restaurants - Guam, 2015. *Preventing Chronic Disease*. 416. Lytle, L. A. Measuring the Food Environment: State of the Science. *American Journal of Preventive Medicine*. 417. Lytle, L. A., & Sokol, R. L. Measures of the food environment: A systematic review of the field, 2007–2015. *Health & Place*. 418. Ma, X., Barnes, T. L., Freedman, D. A., Bell, B. A., Colabianchi, N., & Liese, A. D. Test–retest reliability of a questionnaire measuring perceptions of neighborhood food environment. *Health & Place*. 419. Ma, X., Battersby, S. E., Bell, B. A., Hibbert, J. D., Barnes, T. L., & Liese, A. D. Variation in low food access areas due to data source inaccuracies. *Applied Geography*. 420. MacDonald, J. M., & Nelson, P. E. Do the poor still pay more? Food price variations in large metropolitan areas. *Journal of Urban Economics*. 421. Macdonald, L., Cummins, S., & Macintyre, S. Neighbourhood fast food environment and area deprivation—substitution or concentration? *Appetite*. 422. Mackenbach, J. D., Lakerveld, J., Van Lenthe, F. J., Teixeira, P. J., Compernolle, S., De Bourdeaudhuij, I., . . . Brug, J. Interactions of individual perceived barriers and neighbourhood destinations with obesity-related behaviours in Europe. *Obes Rev*. 423. Maguire, E. R., Burgoine, T., & Monsivais, P. Area deprivation and the food environment over time: A repeated cross-sectional study on takeaway outlet density and supermarket presence in Norfolk, UK, 1990–2008. *Health & Place*. 424. Maguire, E. R., Burgoine, T., Penney, T. L., Forouhi, N. G., & Monsivais, P. Does exposure to the food environment differ by socioeconomic position? Comparing area-based and person-centred metrics in the Fenland Study, UK. *International Journal of Health Geographics*. 425. Mahendra, A., Polsky, J. Y., Robitaille, É., Lefebvre, M., McBrien, T., & Minaker, L. M. Geographic retail food environment measures for use in public health. *Health Promotion and Chronic Disease Prevention in Canada*. 426. Malmstrom, M., Sundquist, J., & Johansson, S. E. Neighborhood environment and self-reported health status: a multilevel analysis. *Am J Public Health*. 427. Mama, S. K., Diamond, P. M., McCurdy, S. A., Evans, A. E., McNeill, L. H., & Lee, R. E. Individual, social and environmental correlates of physical activity in overweight and obese African American and Hispanic women: A structural equation model analysis. *Preventive Medicine Reports*. 428. Martinez, S. M., Ayala, G. X., Patrick, K., Arredondo, E. M., Roesch, S., & Elder, J. Associated pathways between neighborhood environment, community resource factors, and leisure-Time physical activity among mexican-american adults in san diego, california. *American Journal of Health Promotion*. 429. Martinez-Donate, A. P., Espino, J. V., Meinen, A., Escaron, A. L., Roubal, A., Nieto, J., & Malecki, K. Neighborhood Disparities in the Restaurant Food Environment. *Wmj*. 430. Martinez-Donate, A. P., Riggall, A. J., Meinen, A. M., Malecki, K., Escaron, A. L., Hall, B., . . . Nitzke, S. Evaluation of a pilot healthy eating intervention in restaurants and food stores of a rural community: a randomized community trial. *BMC Public Health*. 431. Matthews, S. A., Moudon, A. V., & Daniel, M. Work Group II: Using Geographic Information Systems for Enhancing Research Relevant to Policy on Diet, Physical Activity, and Weight. *American Journal of Preventive Medicine*. 432. Mayer, V. L., Hillier, A., Bachhuber, M. A., & Long, J. A. Food insecurity, neighborhood food access, and food assistance in Philadelphia. *J Urban Health*. 433. Mayne, S. L., Auchincloss, A. H., & Michael, Y. L. Impact of policy and built environment changes on obesity-related outcomes: A systematic review of naturally occurring experiments. *Obesity Reviews*. 434. McAlexander, K. M., Mama, S. K., Medina, A. V., O'Connor, D. P., & Lee, R. E. Concordance and correlates of direct and indirect built environment measurement among minority women. *American Journal of Health Promotion*. 435. McClain, L., & Todd, C. FOOD STORE ACCESSIBILITY. *American Journal of Occupational Therapy*. 436. McKenzie, B. S. Access to supermarkets among poorer neighborhoods: A comparison of time and distance measures. *Urban Geography*. 437. McKinnon, R. A., Reedy, J., Handy, S. L., & Rodgers, A. B. Measuring the Food and Physical Activity Environments: Shaping the Research Agenda. *American Journal of Preventive Medicine*. 438. McKinnon, R. A., Reedy, J., Morrissette, M. A., Lytle, L. A., & Yaroch, A. L. Measures of the Food Environment A Compilation of the Literature, 1990-2007. *American Journal of Preventive Medicine*. 439. Mehta, N. K., & Chang, V. W. Weight status and restaurant availability a multilevel analysis. *Am J Prev Med*. 440. Mendez, D. D., Kim, K. H., Hardaway, C. R., & Fabio, A. Neighborhood racial and socioeconomic disparities in the food and alcohol environment: Are there differences by commercial data sources? *Journal of Racial and Ethnic Health Disparities*. 441. Mercille, G., Richard, L., Gauvin, L., Kestens, Y., Payette, H., & Daniel, M. Comparison of Two Indices of Availability of Fruits/Vegetable and Fast Food Outlets. *Journal of Urban Health-Bulletin of the New York Academy of Medicine*. 442. Mezuk, B., Li, X. J., Cederin, K., Rice, K., Sundquist, J., & Sundquist, K. Beyond Access: Characteristics of the Food Environment and Risk of Diabetes. *American Journal of Epidemiology*. 443. Mhurchu, C. N., Vandevijvere, S., Waterlander, W., Thornton, L. E., Kelly, B., Cameron, A. J., . . . Swinburn, B. Monitoring the availability of healthy and unhealthy foods and non-alcoholic beverages in community and consumer retail food environments globally. *Obesity Research & Clinical Practice*. 444. Middlestadt, S. E., Lederer, A. M., Smith, N. K., Doss, D., Hung, C. L., Stevenson, L. D., & Fly, A. D. Determinants of middle-school students asking parents for fruits and vegetables: a theory-based salient belief elicitation. *Public Health Nutr*. 445. Milder, I. E., Mikolajczak, J., van den Berg, S. W., van de Veen-van Hofwegen, M., & Bemelmans, W. J. Food supply and actions to improve dietary behaviour of students - a comparison between secondary schools participating or not participating in the 'Healthy School Canteen Program'. *Public Health Nutr*. 446. Miller, C., Bodor, J. N., & Rose, D. Measuring the food environment: A systematic technique for characterizing food stores using display counts. *Journal of Environmental and Public Health*. 447. Miller, E. R., 3rd, Cooper, L. A., Carson, K. A., Wang, N. Y., Appel, L. J., Gayles, D., . . . Yeh, H. C. A Dietary Intervention in Urban African Americans: Results of the 'Five Plus Nuts and Beans' Randomized Trial. *Am J Prev Med*. 448. Miller, V., Yusuf, S., Chow, C. K., Dehghan, M., Corsi, D. J., Lock, K., . . . Mente, A. Availability, affordability, and consumption of fruits and vegetables in 18 countries across income levels: findings from the Prospective Urban Rural Epidemiology (PURE) study. *Lancet Glob Health*. 449. Millichamp, A., & Gallegos, D. Comparing the availability, price, variety and quality of fruits and vegetables across retail outlets and by area-level socio-economic position. *Public Health Nutrition*. 450. Minaker, L. M. Retail food environments in Canada: Maximizing the impact of research, policy and practice. *Canadian Journal of Public Health*. 451. Minaker, L. M., Lynch, M., Cook, B. E., & Mah, C. L. Exploring sales data during a healthy corner store intervention in Toronto: The food retail environments shaping health (FRESH) project. *Health Promotion and Chronic Disease Prevention in Canada*. 452. Moayyed, H., Kelly, B., Flood, V., & Todd, J. Measuring the ‘healthiness’ of food outlet types in Australian suburbs’ community food environment. *Obesity Research & Clinical Practice*. 453. Mobley, L. R., Root, E. D., Finkelstein, E. A., Khavjou, O., Farris, R. P., & Will, J. C. Environment, obesity, and cardiovascular disease risk in low-income women. *Am J Prev Med*. 454. Molaodi, O. R., Leyland, A. H., Ellaway, A., Kearns, A., & Harding, S. Neighbourhood food and physical activity environments in England, UK: does ethnic density matter? *International Journal of Behavioral Nutrition and Physical Activity*. 455. Monge-Rojas, R., Smith-Castro, V., Colon-Ramos, U., Aragon, M. C., & Herrera-Raven, F. Psychosocial factors influencing the frequency of fast-food consumption among urban and rural Costa Rican adolescents. *Nutrition*. 456. Mooney, S. J., Richards, C. A., & Rundle, A. G. There goes the neighborhood effect: Bias owing to nondifferential measurement error in the construction of neighborhood contextual measures. *Epidemiology*. 457. Moore, L. V., Carlson, S. A., Onufrak, S., Carroll, D. D., & Galuska, D. Development and implementation of a local government survey to measure community supports for healthy eating and active living. *Preventive Medicine Reports*. 458. Moore, L. V., & Diez Roux, A. V. Associations of Neighborhood Characteristics With the Location and Type of Food Stores. *American Journal of Public Health*. 459. Moore, L. V., Diez Roux, A. V., & Franco, M. Measuring availability of healthy foods: agreement between directly measured and self-reported data. *Am J Epidemiol*. 460. Moran, A., Krepp, E. M., Curtis, C. J., & Lederer, A. An intervention to increase availability of healthy foods and beverages iN New York City hospitals: The healthy hospital food initiative, 2010-2014. *Preventing Chronic Disease*. 461. Moreira, C. C., Moreira, E. A., & Fiates, G. M. Perceived purchase of healthy foods is associated with regular consumption of fruits and vegetables. *J Nutr Educ Behav*. 462. Morland, K., Diez Roux, A. V., & Wing, S. Supermarkets, other food stores, and obesity: the atherosclerosis risk in communities study. *Am J Prev Med*. 463. Morland, K., & Filomena, S. Disparities in the availability of fruits and vegetables between racially segregated urban neighbourhoods. *Public Health Nutrition*. 464. Morland, K., Wing, S., Diez Roux, A., & Poole, C. Neighborhood characteristics associated with the location of food stores and food service places. *American Journal of Preventive Medicine*. 465. Morland, K. B. An Evaluation of a Neighborhood-Level Intervention to a Local Food Environment. *American Journal of Preventive Medicine*. 466. Morland, K. B., & Evenson, K. R. Obesity prevalence and the local food environment. *Health Place*. 467. Mui, Y., Gittelsohn, J., & Jones-Smith, J. C. Longitudinal Associations between Change in Neighborhood Social Disorder and Change in Food Swamps in an Urban Setting. *Journal of Urban Health*. 468. Mui, Y., Jones-Smith, J. C., Thornton, R. L. J., Porter, K. P., & Gittelsohn, J. Relationships between vacant homes and food swamps: A longitudinal study of an urban food environment. *International Journal of Environmental Research and Public Health*. 469. Mui, Y., Lee, B. Y., Adam, A., Kharmats, A. Y., Budd, N., Nau, C., & Gittelsohn, J. Healthy versus Unhealthy Suppliers in Food Desert Neighborhoods: A Network Analysis of Corner Stores' Food Supplier Networks. *Int J Environ Res Public Health*. 470. Mujahid, M. S., Diez Roux, A. V., Shen, M., Gowda, D., Sanchez, B., Shea, S., . . . Jackson, S. A. Relation between neighborhood environments and obesity in the Multi-Ethnic Study of Atherosclerosis. *Am J Epidemiol*. 471. Mundorf, A. R., Willits-Smith, A., & Rose, D. 10 Years Later: Changes in Food Access Disparities in New Orleans since Hurricane Katrina. *Journal of Urban Health*. 472. Munoz-Plaza, C. E., Morland, K. B., Pierre, J. A., Spark, A., Filomena, S. E., & Noyes, P. Navigating the urban food environment: challenges and resilience of community-dwelling older adults. *J Nutr Educ Behav*. 473. Munt, A. E., Partridge, S. R., & Allman-Farinelli, M. The barriers and enablers of healthy eating among young adults: a missing piece of the obesity puzzle: A scoping review. *Obes Rev*. 474. Murakami, K., Sasaki, S., Okubo, H., & Takahashi, Y. Neighborhood socioeconomic status in relation to dietary intake and body mass index in female Japanese dietetic students. *Nutrition*. 475. Murakami, K., Sasaki, S., Takahashi, Y., & Uenishi, K. Neighborhood socioeconomic status in relation to dietary intake and insulin resistance syndrome in female Japanese dietetic students. *Nutrition*. 476. Myers, C. A., Denstel, K. D., & Broyles, S. T. The context of context: Examining the associations between healthy and unhealthy measures of neighborhood food, physical activity, and social environments. *Preventive Medicine*. 477. Myhre, J. B., Loken, E. B., Wandel, M., & Andersen, L. F. Eating location is associated with the nutritional quality of the diet in Norwegian adults. *Public Health Nutr*. 478. Najafabadi, A. T., & Pourhassan, M. Potential for the use of GIS and spatial analysis techniques in nutrition science. *Indian Journal of Public Health Research and Development*. 479. Namba, A., Auchincloss, A., Leonberg, B. L., & Wootan, M. G. Exploratory analysis of fast-food chain restaurant menus before and after implementation of local calorie-labeling policies, 2005-2011. *Prev Chronic Dis*. 480. Nau, C., Ellis, H., Huang, H., Schwartz, B. S., Hirsch, A., Bailey-Davis, L., . . . Glass, T. A. Exploring the forest instead of the trees: An innovative method for defining obesogenic and obesoprotective environments. *Health Place*. 481. Nayga Jr, R. M., & Weinberg, Z. Supermarket access in the inner cities. *Journal of Retailing and Consumer Services*. 482. Neckerman, K. M., Bader, M. D. M., Richards, C. A., Purciel, M., Quinn, J. W., Thomas, J. S., . . . Rundle, A. Disparities in the Food Environments of New York City Public Schools. *American Journal of Preventive Medicine*. 483. Neckerman, K. M., Lovasi, L., Yousefzadeh, P., Sheehan, D., Milinkovic, K., Baecker, A., . . . Rundle, A. Comparing Nutrition Environments in Bodegas and Fast-Food Restaurants. *Journal of the Academy of Nutrition and Dietetics*. 484. Nelson, M. C., Gordon-Larsen, P., Song, Y., & Popkin, B. M. Built and social environments associations with adolescent overweight and activity. *Am J Prev Med*. 485. Nelson, M. C., Larson, N. I., Barr-Anderson, D., Neumark-Sztainer, D., & Story, M. Disparities in dietary intake, meal patterning, and No measure of spatial exposures among young adult nonstudents and 2- and 4-year college students. *American Journal of Public Health*. 486. Nelson, M. C., & Story, M. Food Environments in University Dorms: 20,000 Calories per Dorm Room and Counting. *American Journal of Preventive Medicine*. 487. Nelson, N. M., & Woods, C. B. Obesogenic environments: Are neighbourhood environments that limit physical activity obesogenic? *Health Place*. 488. Nethery, R. C., Warren, J. L., Herring, A. H., Moore, K. A., Evenson, K. R., & Diez-Roux, A. V. A common spatial factor analysis model for measured neighborhood-level characteristics: The Multi-Ethnic Study of Atherosclerosis. *Health Place*. 489. Neuhouser, M. L., Thompson, B., Coronado, G., Martinez, T., & Qu, P. A Household Food Inventory Is Not a Good Measure of Fruit and Vegetable Intake among Ethnically Diverse Rural Women. *Journal of the American Dietetic Association*. 490. Ngamini Ngui, A., & Vanasse, A. Assessing spatial accessibility to mental health facilities in an urban environment. *Spatial and Spatio-temporal Epidemiology*. 491. Nguyen, K. A., de Villiers, A., Fourie, J. M., Bourne, L. T., & Hendricks, M. K. The feasibility of implementing food-based dietary guidelines in the South African primary-school curriculum. *Public Health Nutr*. 492. Ni Mhurchu, C., Blakely, T., Wall, J., Rodgers, A., Jiang, Y., & Wilton, J. Strategies to promote healthier food purchases: a pilot supermarket intervention study. *Public Health Nutr*. 493. Nianogo, R. A., Kuo, T., Smith, L. V., & Arah, O. A. Associations between self-perception of weight, food choice intentions, and consumer response to calorie information: a retrospective investigation of public health center clients in Los Angeles County before the implementation of menu-labeling regulation. *BMC Public Health*. 494. Nies, M. A., Weber, K. T., Holmes, J., Peterson, T., Serr, K., Arias, J., . . . Force, R. Spatial and Census Data to Evaluate Obese Persons and their Environment (SCOPE). *Am J Health Behav*. 495. Oakes, J. M. Invited commentary: Repeated measures, selection bias, and effect identification in neighborhood effect studies. *American Journal of Epidemiology*. 496. Oakes, J. M., Mậsse, L. C., & Messer, L. C. Work Group III: Methodologic Issues in Research on the Food and Physical Activity Environments: Addressing Data Complexity. *American Journal of Preventive Medicine*. 497. O'Connell, M., Buchwald, D. S., & Duncan, G. E. Food Access and Cost in American Indian Communities in Washington State. *Journal of the American Dietetic Association*. 498. Odoms-Young, A. M., Zenk, S., & Mason, M. Measuring Food Availability and Access in African-American Communities: Implications for Intervention and Policy. *American Journal of Preventive Medicine*. 499. Oemichen, M., & Smith, C. Investigation of the Food Choice, Promoters and Barriers to Food Access Issues, and Food Insecurity Among Low-Income, Free-Living Minnesotan Seniors. *J Nutr Educ Behav*. 500. Ohri-Vachaspati, P., Martinez, D., Yedidia, M. J., & Petlick, N. Improving data accuracy of commercial food outlet databases. *American Journal of Health Promotion*. 501. Oka, M., Link, C. L., & Kawachi, I. Area-based variations in obesity are more than a function of the food and physical activity environment: Area-based variations in obesity. *Journal of Urban Health*. 502. O'Kane, G., & Pamphilon, B. The importance of stories in understanding people's relationship to food: narrative inquiry methodology has much to offer the public health nutrition researcher and practitioner. *Public Health Nutr*. 503. Olsho, L. E., Payne, G. H., Walker, D. K., Baronberg, S., Jernigan, J., & Abrami, A. Impacts of a farmers' market incentive programme on fruit and vegetable access, purchase and consumption. *Public Health Nutr*. 504. Orstad, S. L., McDonough, M. H., Stapleton, S., Altincekic, C., & Troped, P. J. A Systematic Review of Agreement Between Perceived and Objective Neighborhood Environment Measures and Associations With No dietary outcomes. *Environment and Behavior*. 505. Oyeyemi, A. L., Kasoma, S. S., Onywera, V. O., Assah, F., Adedoyin, R. A., Conway, T. L., . . . Sallis, J. F. NEWS for Africa: adaptation and reliability of a built environment questionnaire for physical activity in seven African countries. *Int J Behav Nutr Phys Act*. 506. Paalanen, L., Prattala, R., Palosuo, H., & Laatikainen, T. Socio-economic differences in the consumption of vegetables, fruit and berries in Russian and Finnish Karelia: 1992-2007. *Eur J Public Health*. 507. Park, O.-H., Jun, J., & Hoover, L. Measuring the Food Environment of the East Lubbock Community by Using Geographic Information System. *Journal of Nutrition Education and Behavior*. 508. Parr, C. L., Hjartaker, A., Scheel, I., Lund, E., Laake, P., & Veierod, M. B. Comparing methods for handling missing values in food-frequency questionnaires and proposing k nearest neighbours imputation: effects on dietary intake in the Norwegian Women and Cancer study (NOWAC). *Public Health Nutr*. 509. Partington, S. N., Menzies, T. J., Colburn, T. A., Saelens, B. E., & Glanz, K. Reduced-Item Food Audits Based on the Nutrition Environment Measures Surveys. *American Journal of Preventive Medicine*. 510. Payne, G. H., Wethington, H., Olsho, L., Jernigan, J., Farris, R., & Walker, D. K. Implementing a farmers' market incentive program: perspectives on the New York City Health Bucks Program. *Preventing Chronic Disease*. 511. Pearce, J. Neighbourhoods and health: a GIS approach to measuring community resource accessibility. *Journal of Epidemiology & Community Health*. 512. Pearce, J., Blakely, T., Witten, K., & Bartie, P. Neighborhood Deprivation and Access to Fast-Food Retailing. *American Journal of Preventive Medicine*. 513. Pearce, J., Blakely, T., Witten, K., & Bartie, P. Neighborhood deprivation and access to fast-food retailing - A national study. *American Journal of Preventive Medicine*. 514. Pearce, J., Hiscock, R., Blakely, T., & Witten, K. The contextual effects of neighbourhood access to supermarkets and convenience stores on individual fruit and vegetable consumption. *J Epidemiol Community Health*. 515. Pearce, J., Witten, K., & Bartie, P. Neighbourhoods and health: a GIS approach to measuring community resource accessibility. *Journal of Epidemiology and Community Health*. 516. Pearson, A. L., Bentham, G., Day, P., & Kingham, S. Associations between neighbourhood environmental characteristics and obesity and related behaviours among adult New Zealanders. *BMC Public Health*. 517. Pechey, R., & Monsivais, P. Supermarket Choice, Shopping Behavior, Socioeconomic Status, and Food Purchases. *Am J Prev Med*. 518. Pelletier, J. E., Caspi, C. E., Schreiber, L. R., Erickson, D. J., Harnack, L., & Laska, M. N. Successful customer intercept interview recruitment outside small and midsize urban food retailers. *BMC Public Health*. 519. Pelletier, J. E., & Laska, M. N. Campus food and beverage purchases are associated with indicators of diet quality in college students living off campus. *Am J Health Promot*. 520. Penney, T. L., Jones, N. R. V., Adams, J., Maguire, E. R., Burgoine, T., & Monsivais, P. Utilization of Away-From-Home Food Establishments, Dietary Approaches to Stop Hypertension Dietary Pattern, and Obesity. *American Journal of Preventive Medicine*. 521. Pereira, R. F., Sidebottom, A. C., Boucher, J. L., Lindberg, R., & Werner, R. Assessing the food environment of a rural community: Baseline findings from the heart of New Ulm Project, Minnesota, 2010-2011. *Preventing Chronic Disease*. 522. Peters, E. J., & McCreary, T. A. Poor neighbourhoods and the changing geography of food retailing in Saskatoon, Saskatchewan, 1984-2004. *Canadian Journal of Urban Research*. 523. Phipps, E. J., Stites, S. D., Wallace, S. L., & Braitman, L. E. Fresh fruit and vegetable purchases in an urban supermarket by low-income households. *J Nutr Educ Behav*. 524. Pine, A., & Bennett, J. Food access and food deserts: the diverse methods that residents of a neighborhood in Duluth, Minnesota use to provision themselves. *Community Development*. 525. Pineda, E., & Mindell, J. Association of the food environment with obesity: a systematic review of geographical and statistical methods. *The Lancet*. 526. Pitts, S. B., Carr, L. J., Brinkley, J., Byrd, J. L., 3rd, Crawford, T., & Moore, J. B. Associations between neighborhood amenity density and health indicators among rural and urban youth. *Am J Health Promot*. 527. Pitts, S. B. J., Bringolf, K. R., Lloyd, C. L., McGuirt, J. T., Lawton, K. K., & Morgan, J. Formative evaluation for a healthy corner store initiative in pitt county, north carolina: Engaging stakeholders for a healthy corner store initiative, part 2. *Preventing Chronic Disease*. 528. Poelman, M. P., de Vet, E., Velema, E., Seidell, J. C., & Steenhuis, I. H. The No measure of spatial exposure of overweight gatekeepers in the Netherlands. *Public Health Nutr*. 529. Pollard, C. M., Landrigan, T. J., Ellies, P. L., Kerr, D. A., Lester, M. L., & Goodchild, S. E. Geographic factors as determinants of food security: a Western Australian food pricing and quality study. *Asia Pacific journal of clinical nutrition*. 530. Polsky, J. Y., Moineddin, R., Glazier, R. H., Dunn, J. R., & Booth, G. L. Foodscapes of southern Ontario: Neighbourhood deprivation and access to healthy and unhealthy food retail. *Canadian Journal of Public Health-Revue Canadienne De Sante Publique*. 531. Pomerleau, J., Knai, C., Foster, C., Rutter, H., Darmon, N., Derflerova Brazdova, Z., . . . McKee, M. Measuring the food and built environments in urban centres: Reliability and validity of the EURO-PREVOB Community Questionnaire. *Public Health*. 532. Poulos, N. S., & Pasch, K. E. The Outdoor MEDIA DOT: The development and inter-rater reliability of a tool designed to measure food and beverage outlets and outdoor advertising. *Health & Place*. 533. Powell, L. A., Chaloupka, F. J., & Bao, Y. J. The availability of fast-food and full-service restaurants in the United States - Associations with neighborhood characteristics. *American Journal of Preventive Medicine*. 534. Powell, L. M., Auld, M. C., Chaloupka, F. J., O’Malley, P. M., & Johnston, L. D. Associations Between Access to Food Stores and Adolescent Body Mass Index. *American Journal of Preventive Medicine*. 535. Powell, L. M., Chaloupka, F. J., & Bao, Y. The Availability of Fast-Food and Full-Service Restaurants in the United States. Associations with Neighborhood Characteristics. *American Journal of Preventive Medicine*. 536. Powell, L. M., Slater, S., Mirtcheva, D., Bao, Y. J., & Chaloupka, F. J. Food store availability and neighborhood characteristics in the United States. *Preventive Medicine*. 537. Prinsen, S., de Ridder, D. T., & de Vet, E. Eating by example. Effects of environmental cues on dietary decisions. *Appetite*. 538. Pritchard, W. N., & Pritchard, B. Beyond the modern supermarket: Geographical approaches to the analysis of contemporary Australian retail restructuring. *Australian Geographical Studies*. 539. Privitera, G. J., & Zuraikat, F. M. Proximity of foods in a competitive food environment influences consumption of a low calorie and a high calorie food. *Appetite*. 540. Pruchno, R., Wilson-Genderson, M., & Gupta, A. K. Neighborhood food environment and obesity in community-dwelling older adults: individual and neighborhood effects. *Am J Public Health*. 541. Quinn, E., Johnson, D. B., Krieger, J., MacDougall, E., Payne, E., & Chan, N. L. Developing local board of health guidelines to promote healthy food access - King County, Washington, 2010-2012. *Preventing Chronic Disease*. 542. Quintiliani, L. M., Bishop, H. L., Greaney, M. L., & Whiteley, J. A. Factors across home, work, and school domains influence nutrition and physical activity behaviors of nontraditional college students. *Nutr Res*. 543. Racine, E. F., Batada, A., Solomon, C. A., & Story, M. Availability of Foods and Beverages in Supplemental Nutrition Assistance Program−Authorized Dollar Stores in a Region of North Carolina. *Journal of the Academy of Nutrition and Dietetics*. 544. Rahmanian, E., Gasevic, D., Vukmirovich, I., & Lear, S. A. The association between the built environment and dietary intake - a systematic review. *Asia Pac J Clin Nutr*. 545. Raja, S., Yin, L., Roemmich, J., Ma, C., Epstein, L., Yadav, P., & Ticoalu, A. B. Food environment, built environment, and women's bmi: Evidence from erie county, new york. *Journal of Planning Education and Research*. 546. Ransley, J. K., Donnelly, J. K., Khara, T. N., Botham, H., Arnot, H., Greenwood, D. C., & Cade, J. E. The use of supermarket till receipts to determine the fat and energy intake in a UK population. *Public Health Nutr*. 547. Reedy, J., Krebs-Smith, S. M., & Bosire, C. Evaluating the Food Environment: Application of the Healthy Eating Index-2005. *American Journal of Preventive Medicine*. 548. Reitzel, L. R., Regan, S. D., Nguyen, N., Cromley, E. K., Strong, L. L., Wetter, D. W., & McNeill, L. H. Density and proximity of fast food restaurants and body mass index among African Americans. *Am J Public Health*. 549. Richardson, A. S., Meyer, K. A., Howard, A. G., Boone-Heinonen, J., Popkin, B. M., Evenson, K. R., . . . Gordon-Larsen, P. Neighborhood socioeconomic status and food environment: a 20-year longitudinal latent class analysis among CARDIA participants. *Health Place*. 550. Richter, K. P., Harris, K. J., Paine-Andrews, A., Fawcett, S. B., Schmid, T. L., Lankenau, B. H., & Johnston, J. Measuring the Health Environment for Physical Activity and Nutrition among Youth: A Review of the Literature and Applications for Community Initiatives. *Preventive Medicine*. 551. Rideout, K., Levy-Milne, R., Martin, C., & Ostry, A. S. Food sales outlets, food availability, and the extent of nutrition policy implementation in schools in British Columbia. *Canadian Journal of Public Health-Revue Canadienne De Sante Publique*. 552. Rigby, S., Leone, A. F., Kim, H., Betterley, C., Johnson, M. A., Kurtz, H., & Lee, J. S. Food Deserts in Leon County, FL: Disparate Distribution of Supplemental Nutrition Assistance Program–Accepting Stores by Neighborhood Characteristics. *Journal of Nutrition Education and Behavior*. 553. Robles, B., Montes, C. E., Nobari, T. Z., Wang, M. C., & Kuo, T. Dietary Behaviors among Public Health Center Clients with Electronic Benefit Transfer Access at Farmers' Markets. *J Acad Nutr Diet*. 554. Root, E. D. Moving Neighborhoods and Health Research Forward: Using Geographic Methods to Examine the Role of Spatial Scale in Neighborhood Effects on Health. *Annals of the Association of American Geographers*. 555. Roy, R., Kelly, B., Rangan, A., & Allman-Farinelli, M. Food Environment Interventions to Improve the Dietary Behavior of Young Adults in Tertiary Education Settings: A Systematic Literature Review. *J Acad Nutr Diet*. 556. Ruff, R. R., Akhund, A., & Adjoian, T. Small Convenience Stores and the Local Food Environment: An Analysis of Resident Shopping Behavior Using Multilevel Modeling. *Am J Health Promot*. 557. Rummo, P. E., Guilkey, D. K., Ng, S. W., Popkin, B. M., Evenson, K. R., & Gordon-Larsen, P. Beyond Supermarkets: Food Outlet Location Selection in Four US Cities Over Time. *American Journal of Preventive Medicine*. 558. Rundle, A., Diez Roux, A. V., Freeman, L. M., Miller, D., Neckerman, K. M., & Weiss, C. C. The urban built environment and obesity in New York City: A multilevel analysis. *American Journal of Health Promotion*. 559. Rundle, A., Quinn, J., Lovasi, G., Bader, M. D. M., Yousefzadeh, P., Weiss, C., & Neckerman, K. Associations between body mass index and park proximity, size, cleanliness, and recreational facilities. *American Journal of Health Promotion*. 560. Rundle, A. G., Bader, M. D. M., Richards, C. A., Neckerman, K. M., & Teitler, J. O. Using google street view to audit neighborhood environments. *American Journal of Preventive Medicine*. 561. Rushakoff, J. A., Zoughbie, D. E., Bui, N., DeVito, K., Makarechi, L., & Kubo, H. Evaluation of Healthy2Go: A country store transformation project to improve the food environment and consumer choices in Appalachian Kentucky. *Preventive Medicine Reports*. 562. Saba, A., Cupellaro, E., & Vassallo, M. Which dimensions of food-related lifestyle are likely to be associated with obesity in Italy? *Public Health Nutr*. 563. Sacks, R., Yi, S. S., & Nonas, C. Increasing access to fruits and vegetables: Perspectives from the New York City experience. *American Journal of Public Health*. 564. Sadler, R. C. Integrating expert knowledge in a GIS to optimize siting decisions for small-scale healthy food retail interventions. *International Journal of Health Geographics*. 565. Sadler, R. C., Clark, A. F., Wilk, P., O'Connor, C., & Gilliland, J. A. Using GPS and activity tracking to reveal the influence of adolescents' food environment exposure on junk food purchasing. *Canadian Journal of Public Health-Revue Canadienne De Sante Publique*. 566. Sadler, R. C., Gillil, J. A., & Arku, G. A Food retail-based intervention on food security and consumption. *International Journal of Environmental Research and Public Health*. 567. Sadler, R. C., Gilliland, J. A., & Arku, G. COMMUNITY DEVELOPMENT AND THE INFLUENCE OF NEW FOOD RETAIL SOURCES ON THE PRICE AND AVAILABILITY OF NUTRITIOUS FOOD. *Journal of Urban Affairs*. 568. Sadler, R. C., Gilliland, J. A., & Arku, G. A food retail-based intervention on food security and consumption. *Int J Environ Res Public Health*. 569. Saelens, B. E., & Glanz, K. Work Group I: Measures of the Food and Physical Activity Environment: Instruments. *American Journal of Preventive Medicine*. 570. Saelens, B. E., Glanz, K., Sallis, J. F., & Frank, L. D. Nutrition environment measures study in restaurants (NEMS-R) - Development and evaluation. *American Journal of Preventive Medicine*. 571. Sanchez-Flack, J., Pickrel, J. L., Belch, G., Lin, S. F., Anderson, C. A. M., Martinez, M. E., . . . Ayala, G. X. Examination of the relationship between in-store environmental factors and fruit and vegetable purchasing among Hispanics. *International Journal of Environmental Research and Public Health*. 572. Schneider, S., & Gruber, J. Neighbourhood deprivation and outlet density for tobacco, alcohol and fast food: first hints of obesogenic and addictive environments in Germany. *Public Health Nutrition*. 573. Schootman, M., Nelson, E. J., Werner, K., Shacham, E., Elliott, M., Ratnapradipa, K., . . . McVay, A. Emerging technologies to measure neighborhood conditions in public health: implications for interventions and next steps. *International Journal of Health Geographics*. 574. Scully, J. Y., Vernez Moudon, A., Hurvitz, P. M., Aggarwal, A., & Drewnowski, A. GPS or travel diary: Comparing spatial and temporal characteristics of visits to fast food restaurants and supermarkets. *PLoS One*. 575. Seguin, R. A., Morgan, E. H., Connor, L. M., Garner, J. A., King, A. C., Sheats, J. L., . . . Buman, M. P. Rural Food and Physical Activity Assessment Using an Electronic Tablet-Based Application, New York, 2013-2014. *Prev Chronic Dis*. 576. Seliske, L. M., Pickett, W., Boyce, W. F., & Janssen, I. Association between the food retail environment surrounding schools and overweight in Canadian youth. *Public Health Nutr*. 577. Setala, A., Gittelsohn, J., Speakman, K., Oski, J., Martin, T., Moore, R., . . . Bleich, S. N. Linking farmers to community stores to increase consumption of local produce: a case study of the Navajo Nation. *Public Health Nutr*. 578. Shannon, J. Food deserts: Governing obesity in the neoliberal city. *Progress in Human Geography*. 579. Shareck, M., Kestens, Y., & Frohlich, K. L. Moving beyond the residential neighborhood to explore social inequalities in exposure to area-level disadvantage: Results from the Interdisciplinary Study on Inequalities in Smoking. *Soc Sci Med*. 580. Sharkey, J. R. Measuring Potential Access to Food Stores and Food-Service Places in Rural Areas in the US. *American Journal of Preventive Medicine*. 581. Sharkey, J. R., Dean, W. R., & Nalty, C. Convenience Stores and the Marketing of Foods and Beverages Through Product Assortment. *American Journal of Preventive Medicine*. 582. Sharkey, J. R., Dean, W. R., Nalty, C. C., & Xu, J. Convenience stores are the key food environment influence on nutrients available from household food supplies in Texas Border Colonias. *BMC Public Health*. 583. Sharkey, J. R., Horel, S., & Dean, W. R. Neighborhood deprivation, vehicle ownership, and potential spatial access to a variety of fruits and vegetables in a large rural area in Texas. *International Journal of Health Geographics*. 584. Sharkey, J. R., Horel, S., Han, D., & Huber, J. C. Association between neighborhood need and spatial access to food stores and fast food restaurants in neighborhoods of Colonias. *International Journal of Health Geographics*. 585. Sharkey, J. R., Johnson, C. M., Dean, W. R., & Horel, S. A. Focusing on fast food restaurants alone underestimates the relationship between neighborhood deprivation and exposure to fast food in a large rural area. *Nutrition Journal*. 586. Sharp, G., Denney, J. T., & Kimbro, R. T. Multiple contexts of exposure: Activity spaces, residential neighborhoods, and self-rated health. *Soc Sci Med*. 587. Shearer, C., Rainham, D., Blanchard, C., Dummer, T., Lyons, R., & Kirk, S. Measuring food availability and accessibility among adolescents: Moving beyond the neighbourhood boundary. *Soc Sci Med*. 588. Sheldon, M., Gans, K. M., Tai, R., George, T., Lawson, E., & Pearlman, D. N. Availability, affordability, and accessibility of a healthful diet in a low-income community, Central Falls, Rhode Island, 2007-2008. *Prev Chronic Dis*. 589. Shimotsu, S. T., Jones-Webb, R. J., Nelson, T. F., MacLehose, R. F., Lytle, L. A., Forster, J. L., & Van Riper, D. C. Food and alcohol access in neighborhoods of varying socioeconomic status. *Journal of Alcohol and Drug Education*. 590. Short, A., Guthman, J., & Raskin, S. Food deserts, oases, or mirages?: Small markets and community food security in the San Francisco Bay area. *Journal of Planning Education and Research*. 591. Simon, P. A., & Fielding, J. E. Establishing best practices for changing the built environment to promote physical activity. *American Journal of Preventive Medicine*. 592. Simon, P. A., Kwan, D., Angelescu, A., Shih, M., & Fielding, J. E. Proximity of fast food restaurants to schools: Do neighborhood income and type of school matter? *Preventive Medicine*. 593. Singleton, C. R., Li, Y., Duran, A. C., Zenk, S. N., Odoms-Young, A., & Powell, L. M. Food and Beverage Availability in Small Food Stores Located in Healthy Food Financing Initiative Eligible Communities. *International Journal of Environmental Research and Public Health*. 594. Skinner, K., Burnett, K., Williams, P., Martin, D., Stothart, C., LeBlanc, J., . . . Sheedy, A. Challenges in assessing food environments in northern and remote communities in Canada. *Canadian journal of public health = Revue canadienne de sante publique*. 595. Slack, T., Myers, C. A., Martin, C. K., & Heymsfield, S. B. The geographic concentration of US adult obesity prevalence and associated social, economic, and environmental factors. *Obesity (Silver Spring)*. 596. Slater, J., Epp-Koop, S., Jakilazek, M., & Green, C. Food deserts in Winnipeg, Canada: A novel method for measuring a complex and contested construct. *Health Promotion and Chronic Disease Prevention in Canada*. 597. Smith, C., & Miller, H. Accessing the food systems in urban and rural Minnesotan communities. *J Nutr Educ Behav*. 598. Smith, C., & Morton, L. W. Rural Food Deserts: Low-income Perspectives on Food Access in Minnesota and Iowa. *Journal of Nutrition Education and Behavior*. 599. Smith, D. M., Clarke, G. P., Ransley, J., & Cade, J. Food Access and Health: A Microsimulation Framework for Analysis. *Studies in Regional Science*. 600. Smith, D. M., Cummins, S., Taylor, M., Dawson, J., Marshall, D., Sparks, L., & Anderson, A. S. Neighbourhood food environment and area deprivation: spatial accessibility to grocery stores selling fresh fruit and vegetables in urban and rural settings. *International Journal of Epidemiology*. 601. Smith, K. R., Brown, B. B., Yamada, I., Kowaleski-Jones, L., Zick, C. D., & Fan, J. X. Walkability and Body Mass Index. Density, Design, and New Diversity Measures. *American Journal of Preventive Medicine*. 602. Smoyer-Tomic, K. E., Spence, J. C., & Amrhein, C. Food Deserts in the Prairies? Supermarket Accessibility and Neighborhood Need in Edmonton, Canada*. *The Professional Geographer*. 603. Smoyer-Tomic, K. E., Spence, J. C., Raine, K. D., Amrhein, C., Cameron, N., Yasenovskiy, V., . . . Healy, J. The association between neighborhood socioeconomic status and exposure to supermarkets and fast food outlets. *Health & Place*. 604. Smurthwaite, K., & Bagheri, N. Using geographical convergence of obesity, cardiovascular disease, and type 2 diabetes at the neighborhood level to inform policy and practice. *Preventing Chronic Disease*. 605. Sohi, I., Bell, B. A., Liu, J. H., Battersby, S. E., & Liese, A. D. Differences in Food Environment Perceptions and Spatial Attributes of Food Shopping Between Residents of Low and High Food Access Areas. *Journal of Nutrition Education and Behavior*. 606. Song, H. J., Gittelsohn, J., Kim, M., Suratkar, S., Sharma, S., & Anliker, J. A corner store intervention in a low-income urban community is associated with increased availability and sales of some healthy foods. *Public Health Nutrition*. 607. Sparks, A. L., Bania, N., & Leete, L. Comparative approaches to measuring food access in urban areas: The case of Portland, Oregon. *Urban Studies*. 608. Spees, C. K., Clark, J. E., Hooker, N. H., Watowicz, R. P., & Taylor, C. A. Dietary Intake Contributions of Food and Beverages by Source and Food Security Status in US Adults. *Journal of Nutrition Education and Behavior*. 609. Spence, J. C., Cutumisu, N., Edwards, J., Raine, K. D., & Smoyer-Tomic, K. Relation between local food environments and obesity among adults. *BMC Public Health*. 610. Spielman, S. E., Yoo, E. H., & Linkletter, C. Neighborhood contexts, health, and behavior: Understanding the role of scale and residential sorting. *Environment and Planning B: Planning and Design*. 611. Stafford, M., Cummins, S., Ellaway, A., Sacker, A., Wiggins, R. D., & Macintyre, S. Pathways to obesity: identifying local, modifiable determinants of physical activity and diet. *Soc Sci Med*. 612. Stanton, C. A., Green, S. L., & Fries, E. A. Diet-specific Social Support among Rural Adolescents. *Journal of Nutrition Education and Behavior*. 613. Steeves, E. A., Penniston, E., Rowan, M., Steeves, J., & Gittelsohn, J. A Rural Small Food Store Pilot Intervention Creates Trends Toward Improved Healthy Food Availability. *Journal of Hunger & Environmental Nutrition*. 614. Story, M., Giles-Corti, B., Yaroch, A. L., Cummins, S., Frank, L. D., Huang, T. T. K., & Lewis, L. B. Work Group IV: Future Directions for Measures of the Food and Physical Activity Environments. *American Journal of Preventive Medicine*. 615. Story, M., Kaphingst, K. M., Robinson-O'Brien, R., & Glanz, K. Creating healthy food and eating environments: policy and environmental approaches. *Annu Rev Public Health*. 616. Sturm, R. Disparities in the food environment surrounding US middle and high schools. *Public Health*. 617. Suarez, J. J., Isakova, T., Anderson, C. A. M., Boulware, L. E., Wolf, M., & Scialla, J. J. Food Access, Chronic Kidney Disease, and Hypertension in the U.S. *American Journal of Preventive Medicine*. 618. Sun, B., Yan, H., & Zhang, T. Built environmental impacts on individual mode choice and BMI: Evidence from China. *Journal of Transport Geography*. 619. Svastisalee, C., Pedersen, T. P., Schipperijn, J., Jorgensen, S. E., Holstein, B. E., & Krolner, R. Fast-food intake and perceived and objective measures of the local fast-food environment in adolescents. *Public Health Nutrition*. 620. Svastisalee, C. M., Nordah, H., Glumer, C., Holstein, B. E., Powell, L. M., & Due, P. Supermarket and fast-food outlet exposure in Copenhagen: associations with socio-economic and demographic characteristics. *Public Health Nutrition*. 621. Sweeney, G., Hand, M., Kaiser, M., Clark, J. K., Rogers, C., & Spees, C. The State of Food Mapping: Academic Literature Since 2008 and Review of Online GIS-based Food Mapping Resources. *Journal of Planning Literature*. 622. Sylvie, A. K., Jiang, Q., & Cohen, N. Identification of environmental supports for healthy eating in older adults. *J Nutr Gerontol Geriatr*. 623. Tabb, L. P., Ballester, L., & Grubesic, T. H. The spatio-temporal relationship between alcohol outlets and violence before and after privatization: A natural experiment, Seattle, Wa 2010-2013. *Spat Spatiotemporal Epidemiol*. 624. Taillie, L. S., Ng, S. W., & Popkin, B. M. Walmart and Other Food Retail Chains: Trends and Disparities in the Nutritional Profile of Packaged Food Purchases. *American Journal of Preventive Medicine*. 625. Tamura, K., Elbel, B., Chaix, B., Regan, S. D., Al-Ajlouni, Y. A., Athens, J. K., . . . Duncan, D. T. Residential and GPS-Defined Activity Space Neighborhood Noise Complaints, Body Mass Index and Blood Pressure Among Low-Income Housing Residents in New York City. *Journal of Community Health*. 626. Tamura, K., Puett, R. C., Hart, J. E., Starnes, H. A., Laden, F., & Troped, P. J. Spatial clustering of physical activity and obesity in relation to built environment factors among older women in three U.S. states. *BMC Public Health*. 627. Tantiwong, D., & Wilton, P. C. UNDERSTANDING FOOD STORE PREFERENCES AMONG THE ELDERLY USING HYBRID CONJOINT-MEASUREMENT MODELS. *Journal of Retailing*. 628. Tara Zhang, Y., Larai, B. A., Mujahid, M. S., Tamayo, A., Blanchard, S. D., Margaret Warton, E., . . . Karter, A. J. Does food vendor density mediate the association between neighborhood deprivation and BMI? A G-computation mediation analysis. *Epidemiology*. 629. Terry-McElrath, Y. M., O'Malley, P. M., & Johnston, L. D. Foods and beverages offered in US public secondary schools through the National School Lunch Program from 2011–2013: Early evidence of improved nutrition and reduced disparities. *Preventive Medicine*. 630. Tessier, S., Traissac, P., Bricas, N., Maire, B., Eymard-Duvernay, S., El Ati, J., & Delpeuch, F. Food shopping transition: socio-economic characteristics and motivations associated with use of supermarkets in a North African urban environment. *Public Health Nutr*. 631. Thatcher, E., Johnson, C., Zenk, S. N., & Kulbok, P. Retail Food Store Access in Rural Appalachia: A Mixed Methods Study. *Public Health Nursing*. 632. Thompson, C., Cummins, S., Brown, T., & Kyle, R. Understanding interactions with the food environment: an exploration of supermarket food shopping routines in deprived neighbourhoods. *Health Place*. 633. Thompson, J. L., Bentley, G., Davis, M., Coulson, J., Stathi, A., & Fox, K. R. Food shopping habits, physical activity and health-related indicators among adults aged >/=70 years. *Public Health Nutr*. 634. Thornton, L. E., Cameron, A. J., McNaughton, S. A., Waterlander, W. E., Sodergren, M., Svastisalee, C., . . . Crawford, D. A. Does the availability of snack foods in supermarkets vary internationally? *International Journal of Behavioral Nutrition and Physical Activity*. 635. Thornton, L. E., Cameron, A. J., McNaughton, S. A., Worsley, A., & Crawford, D. A. The availability of snack food displays that may trigger impulse purchases in Melbourne supermarkets. *BMC Public Health*. 636. Thornton, L. E., Crawford, D. A., & Ball, K. Who is eating where? Findings from the SocioEconomic Status and Activity in Women (SESAW) study. *Public Health Nutr*. 637. Thornton, L. E., Crawford, D. A., Lamb, K. E., & Ball, K. Where do people purchase food? A novel approach to investigating food purchasing locations. *Int J Health Geogr*. 638. Thornton, L. E., Lamb, K. E., & Ball, K. Fast food restaurant locations according to socioeconomic disadvantage, urban–regional locality, and schools within Victoria, Australia. *SSM - Population Health*. 639. Thornton, L. E., Pearce, J. R., & Ball, K. Sociodemographic factors associated with healthy eating and food security in socio-economically disadvantaged groups in the UK and Victoria, Australia. *Public Health Nutr*. 640. Tian, X., Zhong, L., von Cramon-Taubadel, S., Tu, H., & Wang, H. Restaurants in the Neighborhood, Eating Away from Home and BMI in China. *PLoS One*. 641. Toft, U., Erbs-Maibing, P., & Glumer, C. Identifying fast-food restaurants using a central register as a measure of the food environment. *Scandinavian Journal of Public Health*. 642. Trowbridge, M. J., Gurka, M. J., & O'Connor, R. E. Urban Sprawl and Delayed Ambulance Arrival in the U.S. *American Journal of Preventive Medicine*. 643. Truong, K., Fernandes, M., An, R., Shier, V., & Sturm, R. Measuring the physical food environment and its relationship with obesity: Evidence from California. *Public Health*. 644. Tsai, W. L., Floyd, M. F., Leung, Y. F., McHale, M. R., & Reich, B. J. Urban Vegetative Cover Fragmentation in the U.S.: Associations with Physical Activity and BMI. *American Journal of Preventive Medicine*. 645. Tung, E. L., Peek, M. E., Makelarski, J. A., Escamilla, V., & Lindau, S. T. Adult BMI and Access to Built Environment Resources in a High-Poverty, Urban Geography. *American Journal of Preventive Medicine*. 646. Turrell, G. Structural, material and economic influences on the food-purchasing choices of socioeconomic groups. *Australian and New Zealand Journal of Public Health*. 647. Turrell, G., & Kavanagh, A. M. Socio-economic pathways to diet: modelling the association between socio-economic position and food purchasing behaviour. *Public Health Nutr*. 648. Ulmer, V. M., Rathert, A. R., & Rose, D. Understanding policy enactment: The new orleans fresh food retailer initiative. *American Journal of Preventive Medicine*. 649. Vafaei, A., Pickett, W., Zunzunegui, M. V., & Alvarado, B. E. Neighbourhood social and built environment factors and falls in community-dwelling canadian older adults: A validation study and exploration of structural confounding. *SSM - Population Health*. 650. Valpiani, N., Wilde, P., Rogers, B., & Stewart, H. Patterns of fruit and vegetable availability and price competitiveness across four seasons are different in local food outlets and supermarkets. *Public Health Nutrition*. 651. Van Assema, P., Steenbakkers, M., Stapel, H., Van Keulen, H., Ronda, G., & Brug, J. Evaluation of a Dutch public-private partnership to promote healthier diet. *American Journal of Health Promotion*. 652. van der Horst, K., Timperio, A., Crawford, D., Roberts, R., Brug, J., & Oenema, A. The school food environment associations with adolescent soft drink and snack consumption. *Am J Prev Med*. 653. Van Meter, E., Lawson, A. B., Colabianchi, N., Nichols, M., Hibbert, J., Porter, D., & Liese, A. D. Spatial accessibility and availability measures and statistical properties in the food environment. *Spatial and Spatio-temporal Epidemiology*. 654. Vandevijvere, S., Mackenzie, T., & Ni Mhurchu, C. Indicators of the relative availability of healthy versus unhealthy foods in supermarkets: a validation study. *International Journal of Behavioral Nutrition and Physical Activity*. 655. Vandevijvere, S., Sushil, Z., Exeter, D. J., & Swinburn, B. Obesogenic Retail Food Environments Around New Zealand Schools: A National Study. *American Journal of Preventive Medicine*. 656. Vandevijvere, S., & Swinburn, B. Pilot test of the Healthy Food Environment Policy Index (Food-EPI) to increase government actions for creating healthy food environments. *BMJ Open*. 657. VanKim, N. A., Erickson, D. J., & Laska, M. N. Food shopping profiles and their association with dietary patterns: a latent class analysis. *J Acad Nutr Diet*. 658. Viola, D., Arno, P. S., Maroko, A. R., Schechter, C. B., Sohler, N., Rundle, A., . . . Maantay, J. Overweight and obesity: Can we reconcile evidence about supermarkets and fast food retailers for public health policy. *Journal of Public Health Policy*. 659. Vogel, C., Ntani, G., Inskip, H., Barker, M., Cummins, S., Cooper, C., . . . Baird, J. Education and the Relationship Between Supermarket Environment and Diet. *American Journal of Preventive Medicine*. 660. Walfoort, N. L., Clark, J. J., Bostock, M. J., & O'Neil, K. Active Louisville: Incorporating active living principles into planning and design. *American Journal of Preventive Medicine*. 661. Walker, R. E., Block, J., & Kawachi, I. Do residents of food deserts express different food buying preferences compared to residents of food oases? A mixed-methods analysis. *Int J Behav Nutr Phys Act*. 662. Walker, R. E., Block, J., & Kawachi, I. The Spatial Accessibility of Fast food Restaurants and Convenience Stores in Relation to Neighborhood Schools. *Applied Spatial Analysis and Policy*. 663. Walker, R. E., Keane, C. R., & Burke, J. G. Disparities and access to healthy food in the United States: A review of food deserts literature. *Health & Place*. 664. Wang, H., Tao, L., Qiu, F., & Lu, W. The role of socio-economic status and spatial effects on fresh food access: Two case studies in Canada. *Applied Geography*. 665. Wang, T. W., Agaku, I. T., Marynak, K. L., & King, B. A. Attitudes Toward Prohibiting Tobacco Sales in Pharmacy Stores Among U.S. Adults. *American Journal of Preventive Medicine*. 666. Wang, Y., Wu, Y., & Zhang, Q. Fast-Food Consumption: Its Association with Food Prices and Dietary Quality. *Diet Quality*. 667. Ward, P. R., Coveney, J., Verity, F., Carter, P., & Schilling, M. Cost and affordability of healthy food in rural South Australia. *Rural and Remote Health*. 668. Webber, C. B., Sobal, J., & Dollahite, J. S. Shopping for fruits and vegetables. Food and retail qualities of importance to low-income households at the grocery store. *Appetite*. 669. Wedick, N. M., Ma, Y., Olendzki, B. C., Procter-Gray, E., Cheng, J., Kane, K. J., . . . Li, W. Access to healthy food stores modifies effect of a dietary intervention. *Am J Prev Med*. 670. Wedick, N. M., Ma, Y. S., Olendzki, B. C., Procter-Gray, E., Cheng, J., Kane, K. J., . . . Li, W. J. Access to Healthy Food Stores Modifies Effect of a Dietary Intervention. *American Journal of Preventive Medicine*. 671. Weiss, L., Ompad, D., Galea, S., & Vlahov, D. Defining Neighborhood Boundaries for Urban Health Research. *American Journal of Preventive Medicine*. 672. Wellard, L., Havill, M., Hughes, C., Watson, W. L., & Chapman, K. The availability and accessibility of nutrition information in fast food outlets in five states post-menu labelling legislation in New South Wales. *Australian and New Zealand Journal of Public Health*. 673. Wellard, L., Havill, M., Hughes, C., Watson, W. L., & Chapman, K. Energy-dense fast food products cost less: An observational study of the energy density and energy cost of Australian fast foods. *Australian and New Zealand Journal of Public Health*. 674. Wheeler, A. L., & Chapman-Novakofski, K. Farmers' markets: costs compared with supermarkets, use among WIC clients, and relationship to fruit and vegetable intake and related psychosocial variables. *J Nutr Educ Behav*. 675. Widener, M. J., Farber, S., Neutens, T., & Horner, M. Spatiotemporal accessibility to supermarkets using public transit: an interaction potential approach in Cincinnati, Ohio. *Journal of Transport Geography*. 676. Widener, M. J., Farber, S., Neutens, T., & Horner, M. W. Using urban commuting data to calculate a spatiotemporal accessibility measure for food environment studies. *Health & Place*. 677. Widener, M. J., Metcalf, S. S., & Bar-Yam, Y. Dynamic Urban Food Environments: A Temporal Analysis of Access to Healthy Foods. *American Journal of Preventive Medicine*. 678. Widener, M. J., Minaker, L., Farber, S., Allen, J., Vitali, B., Coleman, P. C., & Cook, B. How do changes in the daily food and transportation environments affect grocery store accessibility? *Applied Geography*. 679. Widener, M. J., & Shannon, J. When are food deserts? Integrating time into research on food accessibility. *Health & Place*. 680. Wiig, K., & Smith, C. The art of grocery shopping on a food stamp budget: factors influencing the food choices of low-income women as they try to make ends meet. *Public Health Nutr*. 681. Wilkins, E. L., Morris, M. A., Radley, D., & Griffiths, C. Using Geographic Information Systems to measure retail food environments: Discussion of methodological considerations and a proposed reporting checklist (Geo-FERN). *Health & Place*. 682. Wilkins, J. L., Farrell, T. J., & Rangarajan, A. Linking vegetable preferences, health and local food systems through community-supported agriculture. *Public Health Nutr*. 683. Williams, J., Scarborough, P., Matthews, A., Foster, C., Cowburn, G., Roberts, N., & Rayner, M. Influence of the retail food environment around schools on obesity-related outcomes: a systematic review. *The Lancet*. 684. Williams, L. K., Thornton, L., Ball, K., & Crawford, D. Is the objective food environment associated with perceptions of the food environment? *Public Health Nutr*. 685. Williams, L. K., Thornton, L., Crawford, D., & Ball, K. Perceived quality and availability of fruit and vegetables are associated with perceptions of fruit and vegetable affordability among socio-economically disadvantaged women. *Public Health Nutr*. 686. Wineman, J. D., Marans, R. W., Schulz, A. J., van der Westhuizen, D. L., Mentz, G. B., & Max, P. Designing Healthy Neighborhoods: Contributions of the Built Environment to Physical Activity in Detroit. *Journal of Planning Education and Research*. 687. Winkler, E., Turrell, G., & Patterson, C. Does living in a disadvantaged area mean fewer opportunities to purchase fresh fruit and vegetables in the area? Findings from the Brisbane food study. *Health & Place*. 688. Wolf-Powers, L. Food Deserts and Real-Estate-Led Social Policy. *International Journal of Urban and Regional Research*. 689. Wolfson, J. A., & Bleich, S. N. Is cooking at home associated with better diet quality or weight-loss intention? *Public Health Nutr*. 690. Wong, M. S., Chan, K. S., Jones-Smith, J. C., Colantuoni, E., Thorpe Jr, R. J., & Bleich, S. N. The neighborhood environment and obesity: Understanding variation by race/ethnicity. *Preventive Medicine*. 691. Wong, M. S., Peyton, J. M., Shields, T. M., Curriero, F. C., & Gudzune, K. A. Comparing the accuracy of food outlet datasets in an urban environment. *Geospatial Health*. 692. Wood, B. S., & Horner, M. W. Understanding Accessibility to Snap-Accepting Food Store Locations: Disentangling the Roles of Transportation and Socioeconomic Status. *Applied Spatial Analysis and Policy*. 693. Woodruff, R. C., Coleman, A. M., Hermstad, A. K., Honeycutt, S., Munoz, J., Loh, L., . . . Kegler, M. C. Increasing community access to fresh fruits and vegetables: A case study of the farm fresh market pilot program in Cobb County, Georgia, 2014. *Preventing Chronic Disease*. 694. Wrigley, N. 'Food deserts' in British cities: Policy context and research priorities. *Urban Studies*. 695. Xu, Y., Wen, M., & Wang, F. Multilevel built environment features and individual odds of overweight and obesity in Utah. *Applied Geography*. 696. Xue, H., Cheng, X., Zhang, Q., Wang, H., Zhang, B., Qu, W., & Wang, Y. Temporal growth and spatial distribution of the fast food industry and its relationship with economic development in China — 2005–2012. *Preventive Medicine*. 697. Yenerall, J., You, W., & Hill, J. Investigating the Spatial Dimension of Food Access. *Int J Environ Res Public Health*. 698. Yi, S., Kanetkar, V., & Brauer, P. Assessment of heterogeneity in types of vegetables served by main household food preparers and food decision influencers. *Public Health Nutr*. 699. Young, C. R., Aquilante, J. L., Solomon, S., Colby, L., Kawinzi, M. A., Uy, N., & Mallya, G. Improving fruit and vegetable consumption among low-income customers at farmers markets: Philly food bucks, Philadelphia, Pennsylvania, 2011. *Preventing Chronic Disease*. 700. Zenk, S. N., Grigsby-Toussaint, D. S., Curry, S. J., Berbaum, M., & Schneider, L. Short-term Temporal Stability in Observed Retail Food Characteristics. *Journal of Nutrition Education and Behavior*. 701. Zenk, S. N., Horoi, I., McDonald, A., Corte, C., Riley, B., & Odoms-Young, A. M. Ecological momentary assessment of environmental and personal factors and snack food intake in African American women. *Appetite*. 702. Zenk, S. N., Mentz, G., Schulz, A. J., Johnson-Lawrence, V., & Gaines, C. R. Longitudinal Associations Between Observed and Perceived Neighborhood Food Availability and Body Mass Index in a Multiethnic Urban Sample. *Health Education & Behavior*. 703. Zenk, S. N., Odoms-Young, A., Powell, L. M., Campbell, R. T., Block, D., Chavez, N., . . . Armbruster, J. Fruit and Vegetable Availability and Selection: Federal Food Package Revisions, 2009. *American Journal of Preventive Medicine*. 704. Zenk, S. N., Schulz, A. J., Hollis-Neely, T., Campbell, R. T., Holmes, N., Watkins, G., . . . Odoms-Young, A. Fruit and vegetable intake in African Americans income and store characteristics. *Am J Prev Med*. 705. Zenk, S. N., Schulz, A. J., Israel, B. A., James, S. A., Bao, S., & Wilson, M. L. Neighborhood Racial Composition, Neighborhood Poverty, and the Spatial Accessibility of Supermarkets in Metropolitan Detroit. *American Journal of Public Health*. 706. Zenk, S. N., Schulz, A. J., Israel, B. A., Mentz, G., Miranda, P. Y., Opperman, A., & Odoms-Young, A. M. Food shopping behaviours and exposure to discrimination. *Public Health Nutr*. 707. Zenk, S. N., Tarlov, E., Wing, C., Matthews, S. A., Jones, K., Tong, H., & Powell, L. M. Geographic Accessibility Of Food Outlets Not Associated With Body Mass Index Change Among Veterans, 2009-14. *Health Affairs*. 708. Zhang, X., van der Lans, I., & Dagevos, H. Impacts of fast food and the food retail environment on overweight and obesity in China: a multilevel latent class cluster approach. *Public Health Nutr*. 709. Zick, C. D., Smith, K. R., Kowaleski-Jones, L., Uno, C., & Merrill, B. J. Harvesting more than vegetables: the potential weight control benefits of community gardening. *Am J Public Health*. 710. Zuberi, A., Duck, W., Gradeck, B., & Hopkinson, R. NEIGHBORHOODS, RACE, AND HEALTH: EXAMINING THE RELATIONSHIP BETWEEN NEIGHBORHOOD DISTRESS AND BIRTH OUTCOMES IN PITTSBURGH. *Journal of Urban Affairs*. | 1. No measure of spatial exposure 2. No dietary outcome 3. No dietary outcome 4. No dietary outcome 5. No measure of spatial exposure 6. No dietary outcome 7. No dietary outcome 8. No dietary outcome 9. No measure of spatial exposure 10. No dietary outcome 11. No measure of spatial exposure 12. No measure of spatial exposure 13. No dietary outcome 14. No dietary outcome 15. No dietary outcome 16. No measure of spatial exposure 17. No dietary outcome 18. No dietary outcome 19. Exposure not within residential area 20. No dietary outcome 21. No dietary outcome 22. No measure of spatial exposure 23. No measure of spatial exposure 24. No dietary outcome 25. ≤ 18 years of age 26. No dietary outcome 27. No dietary outcome 28. No dietary outcome 29. No dietary outcome 30. No dietary outcome 31. No dietary outcome 32. No dietary outcome 33. No dietary outcome 34. No dietary outcome 35. Exposure not within residential area 36. No dietary outcome 37. No dietary outcome 38. No dietary outcome 39. No measure of spatial exposure 40. No dietary outcome 41. No dietary outcome 42. No dietary outcome 43. No measure of spatial exposure 44. No dietary outcome 45. No dietary outcome 46. No dietary outcome 47. No dietary outcome 48. No dietary outcome 49. No dietary outcome 50. No dietary outcome 51. No dietary outcome 52. No dietary outcome 53. No dietary outcome 54. No measure of spatial exposure 55. No dietary outcome 56. No dietary outcome 57. Review 58. No measure of spatial exposure 59. No dietary outcome 60. Exposure not within residential area 61. No dietary outcome 62. No measure of spatial exposure 63. No measure of spatial exposure 64. No dietary outcome 65. No measure of spatial exposure 66. No dietary outcome 67. No dietary outcome 68. No dietary outcome 69. No dietary outcome 70. No measure of spatial exposure 71. No dietary outcome 72. No measure of spatial exposure 73. No measure of spatial exposure 74. No dietary outcome 75. ≤ 18 years of age 76. No dietary outcome 77. ≤ 18 years of age 78. No dietary outcome 79. No dietary outcome 80. No dietary outcome 81. ≤ 18 years of age 82. No dietary outcome 83. No measure of spatial exposure 84. No dietary outcome 85. No dietary outcome 86. No measure of spatial exposure 87. No dietary outcome 88. No dietary outcome 89. No dietary outcome 90. No measure of spatial exposure 91. No dietary outcome 92. No dietary outcome 93. No dietary outcome 94. No dietary outcome 95. No dietary outcome 96. No dietary outcome 97. No dietary outcome 98. No dietary outcome 99. No dietary outcome 100. Exposure not within residential area 101. No dietary outcome 102. No measure of spatial exposure 103. No dietary outcome 104. No dietary outcome 105. No measure of spatial exposure 106. No dietary outcome 107. No dietary outcome 108. No measure of spatial exposure 109. No measure of spatial exposure 110. No dietary outcome 111. No dietary outcome 112. Exposure not within residential area 113. No dietary outcome 114. No dietary outcome 115. ≤ 18 years of age 116. No measure of spatial exposure 117. No measure of spatial exposure 118. No measure of spatial exposure 119. No dietary outcome 120. No dietary outcome 121. A review 122. No dietary outcome 123. No dietary outcome 124. No dietary outcome 125. No dietary outcome 126. No dietary outcome 127. No dietary outcome 128. A review 129. A review 130. No dietary outcome 131. No dietary outcome 132. No measure of spatial exposure 133. No measure of spatial exposure 134. No measure of spatial exposure 135. No dietary outcome 136. No dietary outcome 137. No dietary outcome 138. No dietary outcome 139. No dietary outcome 140. Exposure not within residential area 141. No dietary outcome 142. No dietary outcome 143. No dietary outcome 144. No measure of spatial exposure 145. No dietary outcome 146. No dietary outcome 147. Exposure not within residential area 148. No dietary outcome 149. No dietary outcome 150. No measure of spatial exposure 151. No dietary outcome 152. No measure of spatial exposure 153. No dietary outcome 154. No dietary outcome 155. No dietary outcome 156. No dietary outcome 157. ≤ 18 years of age 158. No dietary outcome 159. Exposure not within residential area 160. No dietary outcome 161. No dietary outcome 162. No dietary outcome 163. No dietary outcome 164. No dietary outcome 165. No dietary outcome 166. No dietary outcome 167. No dietary outcome 168. No dietary outcome 169. No dietary outcome 170. No dietary outcome 171. No measure of spatial exposure 172. No dietary outcome 173. No dietary outcome 174. No measure of spatial exposure 175. No measure of spatial exposure 176. Exposure not within residential area 177. No measure of spatial exposure 178. No measure of spatial exposure 179. No measure of spatial exposure 180. No dietary outcome 181. No dietary outcome 182. No measure of spatial exposure 183. No measure of spatial exposure 184. Exposure not within residential area 185. ≤ 18 years of age 186. No dietary outcome 187. No dietary outcome 188. No dietary outcome 189. No dietary outcome 190. No dietary outcome 191. No dietary outcome 192. No dietary outcome 193. No dietary outcome 194. No dietary outcome 195. No dietary outcome 196. No dietary outcome 197. No measure of spatial exposure 198. No measure of spatial exposure 199. No dietary outcome 200. No dietary outcome 201. No dietary outcome 202. No dietary outcome 203. A review 204. No dietary outcome 205. No dietary outcome 206. No dietary outcome 207. No measure of spatial exposure 208. No measure of spatial exposure 209. A review 210. No dietary outcome 211. No measure of spatial exposure 212. No dietary outcome 213. No dietary outcome 214. No measure of spatial exposure 215. No dietary outcome 216. ≤ 18 years of age 217. Exposure not within residential area 218. No dietary outcome 219. A review 220. A review 221. No dietary outcome 222. No dietary outcome 223. No measure of spatial exposure 224. No dietary outcome 225. No dietary outcome 226. No dietary outcome 227. ≤ 18 years of age 228. No dietary outcome 229. No dietary outcome 230. No dietary outcome 231. No dietary outcome 232. No dietary outcome 233. Exposure not within residential area 234. A review 235. No measure of spatial exposure 236. No dietary outcome 237. No measure of spatial exposure 238. No dietary outcome 239. No dietary outcome 240. No dietary outcome 241. No dietary outcome 242. No measure of spatial exposure 243. No dietary outcome 244. No measure of spatial exposure 245. No dietary outcome 246. No dietary outcome 247. No dietary outcome 248. No dietary outcome 249. No dietary outcome 250. No dietary outcome 251. A review 252. No measure of spatial exposure 253. No measure of spatial exposure 254. No measure of spatial exposure 255. No measure of spatial exposure 256. No dietary outcome 257. No dietary outcome 258. A review 259. No measure of spatial exposure 260. No dietary outcome 261. No dietary outcome 262. No dietary outcome 263. No measure of spatial exposure 264. No measure of spatial exposure 265. No measure of spatial exposure 266. No dietary outcome 267. No dietary outcome 268. No dietary outcome 269. No dietary outcome 270. No measure of spatial exposure 271. No dietary outcome 272. Exposure not within residential area 273. No dietary outcome 274. No dietary outcome 275. No dietary outcome 276. ≤ 18 years of age 277. No measure of spatial exposure 278. No dietary outcome 279. No measure of spatial exposure 280. ≤ 18 years of age 281. ≤ 18 years of age 282. ≤ 18 years of age 283. No dietary outcome 284. No dietary outcome 285. No dietary outcome 286. No dietary outcome 287. No measure of spatial exposure 288. A review 289. No measure of spatial exposure 290. No dietary outcome 291. No dietary outcome 292. No measure of spatial exposure 293. No dietary outcome 294. No dietary outcome 295. No dietary outcome 296. No dietary outcome 297. No dietary outcome 298. No dietary outcome 299. Diet assessment only 300. No dietary outcome 301. No dietary outcome 302. No dietary outcome 303. No measure of spatial exposure 304. No dietary outcome 305. No dietary outcome 306. No dietary outcome 307. No measure of spatial exposure 308. No measure of spatial exposure 309. ≤ 18 years of age 310. No dietary outcome 311. No dietary outcome 312. No dietary outcome 313. No measure of spatial exposure 314. No dietary outcome 315. No dietary outcome 316. No measure of spatial exposure 317. No dietary outcome 318. A review 319. No dietary outcome 320. No dietary outcome 321. No dietary outcome 322. No measure of spatial exposure 323. No dietary outcome 324. No dietary outcome 325. No dietary outcome 326. No measure of spatial exposure 327. No measure of spatial exposure 328. No measure of spatial exposure 329. No dietary outcome 330. No dietary outcome 331. ≤ 18 years of age 332. No dietary outcome 333. No dietary outcome 334. No dietary outcome 335. No dietary outcome 336. No dietary outcome 337. No dietary outcome 338. No dietary outcome 339. No measure of spatial exposure 340. A review 341. No dietary outcome 342. No measure of spatial exposure 343. Exposure not within residential area 344. No dietary outcome 345. Exposure not within residential area 346. No measure of spatial exposure 347. No dietary outcome 348. No dietary outcome 349. No dietary outcome 350. No dietary outcome 351. No dietary outcome 352. A review 353. No dietary outcome 354. No dietary outcome 355. No measure of spatial exposure 356. No measure of spatial exposure 357. No measure of spatial exposure 358. Exposure not within residential area 359. No measure of spatial exposure 360. No measure of spatial exposure 361. No dietary outcome 362. Exposure not within residential area 363. No dietary outcome 364. No dietary outcome 365. A review 366. No dietary outcome 367. ≤ 18 years of age 368. No measure of spatial exposure 369. No dietary outcome 370. No dietary outcome 371. No dietary outcome 372. No measure of spatial exposure 373. No dietary outcome 374. No dietary outcome 375. No dietary outcome 376. No measure of spatial exposure 377. No measure of spatial exposure 378. ≤ 18 years of age 379. No measure of spatial exposure 380. Exposure not within residential area 381. No dietary outcome 382. No dietary outcome 383. No dietary outcome 384. No dietary outcome 385. No dietary outcome 386. No dietary outcome 387. No dietary outcome 388. No dietary outcome 389. No dietary outcome 390. No measure of spatial exposure 391. No dietary outcome 392. No measure of spatial exposure 393. No dietary outcome 394. No dietary outcome 395. No dietary outcome 396. No dietary outcome 397. Mobile vendor 398. No measure of spatial exposure 399. No measure of spatial exposure 400. No dietary outcome 401. No dietary outcome 402. No dietary outcome 403. No measure of spatial exposure 404. No dietary outcome 405. No dietary outcome 406. No measure of spatial exposure 407. Exposure not within residential area 408. No dietary outcome 409. No dietary outcome 410. No dietary outcome 411. No dietary outcome 412. No measure of spatial exposure 413. Mobile vendors 414. No measure of spatial exposure 415. No dietary outcome 416. No measure of spatial exposure 417. A review 418. A review 419. No dietary outcome 420. No dietary outcome 421. No dietary outcome 422. No dietary outcome 423. No dietary outcome 424. No dietary outcome 425. No dietary outcome 426. No dietary outcome 427. No dietary outcome 428. No dietary outcome 429. No dietary outcome 430. No dietary outcome 431. No measure of spatial exposure 432. No dietary outcome 433. No dietary outcome 434. No dietary outcome 435. No dietary outcome 436. No measure of spatial exposure 437. No dietary outcome 438. A review 439. No dietary outcome 440. No dietary outcome 441. No dietary outcome 442. No dietary outcome 443. No dietary outcome 444. No dietary outcome 445. Exposure not within residential area 446. Exposure not within residential area 447. No measure of spatial exposure 448. No measure of spatial exposure 449. No dietary outcome 450. No dietary outcome 451. No dietary outcome 452. No dietary outcome 453. No dietary outcome 454. No dietary outcome 455. No dietary outcome 456. ≤ 18 years of age 457. No dietary outcome 458. No dietary outcome 459. No dietary outcome 460. No measure of spatial exposure 461. No dietary outcome 462. No measure of spatial exposure 463. No dietary outcome 464. No dietary outcome 465. No dietary outcome 466. No dietary outcome 467. No dietary outcome 468. No dietary outcome 469. No measure of spatial exposure 470. No dietary outcome 471. No measure of spatial exposure 472. No measure of spatial exposure 473. No dietary outcome 474. No dietary outcome 475. No dietary outcome 476. No dietary outcome 477. No dietary outcome 478. No dietary outcome 479. No dietary outcome 480. No measure of spatial exposure 481. No dietary outcome 482. No dietary outcome 483. No dietary outcome 484. No dietary outcome 485. Exposure not within residential area 486. No dietary outcome 487. ≤ 18 years of age 488. No measure of spatial exposure 489. No dietary outcome 490. No dietary outcome 491. No dietary outcome 492. No measure of spatial exposure 493. No dietary outcome 494. Exposure not within residential area 495. No measure of spatial exposure 496. No dietary outcome 497. No dietary outcome 498. No dietary outcome 499. No dietary outcome 500. No dietary outcome 501. No dietary outcome 502. No dietary outcome 503. No dietary outcome 504. No dietary outcome 505. No dietary outcome 506. No measure of spatial exposure 507. A review 508. No dietary outcome 509. No dietary outcome 510. No dietary outcome 511. No dietary outcome 512. No dietary outcome 513. No dietary outcome 514. No dietary outcome 515. No dietary outcome 516. No measure of spatial exposure 517. No dietary outcome 518. No dietary outcome 519. No dietary outcome 520. No dietary outcome 521. No dietary outcome 522. Campus food environment 523. No dietary outcome 524. No dietary outcome 525. No measure of spatial exposure 526. No dietary outcome 527. No dietary outcome 528. No measure of spatial exposure 529. ≤ 18 years of age 530. No measure of spatial exposure 531. No dietary outcome 532. No dietary outcome 533. No dietary outcome 534. No dietary outcome 535. No dietary outcome 536. No measure of spatial exposure 537. ≤ 18 years of age 538. No measure of spatial exposure 539. No dietary outcome 540. No measure of spatial exposure 541. No dietary outcome 542. No dietary outcome 543. Exposure not within residential area 544. No measure of spatial exposure 545. A review 546. No dietary outcome 547. No dietary outcome 548. No measure of spatial exposure 549. No dietary outcome 550. No dietary outcome 551. ≤ 18 years of age 552. Exposure not within residential area 553. No measure of spatial exposure 554. No dietary outcome 555. No dietary outcome 556. A review 557. No measure of spatial exposure 558. No dietary outcome 559. No dietary outcome 560. No dietary outcome 561. No dietary outcome 562. No measure of spatial exposure 563. No dietary outcome 564. No dietary outcome 565. No dietary outcome 566. ≤ 18 years of age 567. No measure of spatial exposure 568. No dietary outcome 569. No dietary outcome 570. No dietary outcome 571. No measure of spatial exposure 572. No dietary outcome 573. No dietary outcome 574. No dietary outcome 575. No dietary outcome 576. Exposure not within residential area 577. No measure of spatial exposure 578. No dietary outcome 579. No dietary outcome 580. No measure of spatial exposure 581. No measure of spatial exposure 582. No dietary outcome 583. No measure of spatial exposure 584. No dietary outcome 585. No measure of spatial exposure 586. No dietary outcome 587. ≤ 18 years of age 588. No dietary outcome 589. No dietary outcome 590. No dietary outcome 591. No dietary outcome 592. Exposure not within residential area 593. No measure of spatial exposure 594. No dietary outcome 595. No dietary outcome 596. No dietary outcome 597. No dietary outcome 598. No dietary outcome 599. No dietary outcome 600. No measure of spatial exposure 601. No dietary outcome 602. No dietary outcome 603. No dietary outcome 604. No dietary outcome 605. No dietary outcome 606. No dietary outcome 607. No measure of spatial exposure 608. No dietary outcome 609. No measure of spatial exposure 610. No dietary outcome 611. No dietary outcome 612. No dietary outcome 613. ≤ 18 years of age 614. No measure of spatial exposure 615. No dietary outcome 616. No dietary outcome 617. Exposure not within residential area 618. No dietary outcome 619. No dietary outcome 620. No dietary outcome 621. ≤ 18 years of age 622. A review 623. No measure of spatial exposure 624. No measure of spatial exposure 625. No dietary outcome 626. No dietary outcome 627. No dietary outcome 628. No measure of spatial exposure 629. No dietary outcome 630. Exposure not within residential area 631. No dietary outcome 632. No measure of spatial exposure 633. No dietary outcome 634. No dietary outcome 635. No dietary outcome 636. No dietary outcome 637. No dietary outcome 638. No dietary outcome 639. No dietary outcome 640. Exposure not within residential area 641. No measure of spatial exposure 642. No dietary outcome 643. No dietary outcome 644. No dietary outcome 645. No dietary outcome 646. No dietary outcome 647. No dietary outcome 648. No dietary outcome 649. No dietary outcome 650. No dietary outcome 651. No dietary outcome 652. No dietary outcome 653. Exposure not within residential area 654. No dietary outcome 655. No dietary outcome 656. No measure of spatial exposure 657. Exposure not within residential area 658. No measure of spatial exposure 659. No dietary outcome 660. No dietary outcome 661. No dietary outcome 662. Exposure not within residential area 663. No dietary outcome 664. A review 665. No dietary outcome 666. No measure of spatial exposure 667. No measure of spatial exposure 668. No dietary outcome 669. No measure of spatial exposure 670. No measure of spatial exposure 671. No dietary outcome 672. No dietary outcome 673. No dietary outcome 674. No measure of spatial exposure 675. No dietary outcome 676. No measure of spatial exposure 677. No dietary outcome 678. No dietary outcome 679. No dietary outcome 680. No dietary outcome 681. A review 682. No dietary outcome 683. Exposure not within residential area 684. No dietary outcome 685. No dietary outcome 686. No dietary outcome 687. No dietary outcome 688. No dietary outcome 689. No measure of spatial exposure 690. No dietary outcome 691. No dietary outcome 692. No measure of spatial exposure 693. No dietary outcome 694. No dietary outcome 695. No dietary outcome 696. No dietary outcome 697. No dietary outcome 698. No dietary outcome 699. No measure of spatial exposure 700. No measure of spatial exposure 701. No dietary outcome 702. No measure of spatial exposure 703. No dietary outcome 704. No dietary outcome 705. No dietary outcome 706. No dietary outcome 707. No dietary outcome 708. No dietary outcome 709. No dietary outcome 710. No dietary outcome |

| **Citations excluded based on full text review** | **Reason** |
| --- | --- |
| 1. Menezes, M. C., Costa, B. V. L., Oliveira, C. D. L., & Lopes, A. C. S. Local food environment and fruit and vegetable consumption: An ecological study. *Preventive Medicine Reports*. 2. Meyer, K. A., Boone-Heinonen, J., Duffey, K. J., Rodriguez, D. A., Kiefe, C. I., Lewis, C. E., & Gordon-Larsen, P. Combined measure of neighborhood food and physical activity environments and weight-related outcomes: The CARDIA study. *Health Place*. 3. Pessoa, M. C., Mendes, L. L., Caiaffa, W. T., Malta, D. C., & Velasquez-Melendez, G. Availability of food stores and consumption of fruit, legumes and vegetables in a Brazilian urban area. *Nutricion Hospitalaria*. 4. Posner, S. F. Eating Patterns, Body Mass Index, and Food Deserts: Does It Matter Where We Live? *Preventing Chronic Disease*. 5. Thornton, L. E., Lamb, K. E., Tseng, M., Crawford, D. A., & Ball, K. Does food store access modify associations between intrapersonal factors and fruit and vegetable consumption? *Eur J Clin Nutr*. 6. Wrigley, N., Warm, D., Margetts, B., & Whelan, A. Assessing the impact of improved retail access on diet in a 'food desert': A preliminary report. *Urban Studies*. 7. Burgoine, T., & Monsivais, P. Characterising food environment exposure at home, at work, and along commuting journeys using data on adults in the UK. *Int J Behav Nutr Phys Act*. 8. Chen, X., & Yang, X. Does food environment influence food choices? A geographical analysis through “tweets”. *Applied Geography*. 9. James, P., Seward, M. W., James O'Malley, A., Subramanian, S. V., & Block, J. P. Changes in the food environment over time: examining 40 years of data in the Framingham Heart Study. *Int J Behav Nutr Phys Act*. 10. Kestens, Y., Lebel, A., Daniel, M., Thériault, M., & Pampalon, R. Using experienced activity spaces to measure foodscape exposure. *Health & Place*. 11. Moore, L. V., Roux, A. V. D., & Brines, S. Comparing perception-based and geographic information system (GIS)-Based characterizations of the local food environment. *Journal of Urban Health-Bulletin of the New York Academy of Medicine*. 12. Budzynska, K., West, P., Savoy-Moore, R. T., Lindsey, D., Winter, M., & Newby, P. K. A food desert in Detroit: associations with food shopping and eating behaviours, dietary intakes and obesity. *Public Health Nutr*. 13. Cheadle, A., Psaty, B. M., Curry, S., Wagner, E., Diehr, P., Koepsell, T., & Kristal, A. Can Measures of the Grocery Store Environment Be Used to Track Community-Level Dietary Changes? *Preventive Medicine*. 14. Clary, C., Matthews, S. A., & Kestens, Y. Between exposure, access and use: Reconsidering foodscape influences on dietary behaviours. *Health & Place*. 15. Cummins, S. Large scale food retailing as an intervention for diet and health: quasi-experimental evaluation of a natural experiment. *Journal of Epidemiology & Community Health*. 16. D'Angelo, H., Suratkar, S., Song, H. J., Stauffer, E., & Gittelsohn, J. Access to food source and food source use are associated with healthy and unhealthy food-purchasing behaviours among low-income African-American adults in Baltimore City. *Public Health Nutr*. 17. Dubowitz, T., Ghosh-Dastidar, M., Cohen, D. A., Beckman, R., Steiner, E. D., Hunter, G. P., . . . Collins, R. L. Diet and perceptions change with supermarket introduction in a food desert, but not because of supermarket use. *Health Affairs*. 18. Dubowitz, T., Subramanian, S. V., Acevedo-Garcia, D., Osypuk, T. L., & Peterson, K. E. Individual and neighborhood differences in diet among low-income foreign and U.S.-born women. *Womens Health Issues*. 19. Iwama, N., Asakawa, T., Tanaka, K., & Komaki, N. Analysis of the factors that disrupt dietary habits in the elderly: A case study of a Japanese food desert. *Urban Studies*. 20. Jilcott Pitts, S. B., Acheson, M. L. M., Ward, R. K., Wu, Q., McGuirt, J. T., Bullock, S. L., . . . Ammerman, A. S. Disparities in healthy food zoning, farmers' market availability, and fruit and vegetable consumption among North Carolina residents. *Archives of Public Health*. 21. Kegler, M. C., Swan, D. W., Alcantara, I., Feldman, L., & Glanz, K. The influence of rural home and neighborhood environments on healthy eating, physical activity, and weight. *Prev Sci*. 22. Mead, E., Gittelsohn, J., Kratzmann, M., Roache, C., & Sharma, S. Impact of the changing food environment on dietary practices of an Inuit population in Arctic Canada. *J Hum Nutr Diet*. 23. Morland, K., & Filomena, S. The utilization of local food environments by urban seniors. *Prev Med*. 24. Rose, D., & Richards, R. Food store access and household fruit and vegetable use among participants in the US Food Stamp Program. *Public Health Nutr*. 25. Smith, C., Butterfass, J., & Richards, R. Environment influences food access and resulting shopping and dietary behaviors among homeless Minnesotans living in food deserts. *Agriculture and Human Values*. 26. Thornton, L. E., Ball, K., Lamb, K. E., McCann, J., Parker, K., & Crawford, D. A. The impact of a new McDonald's restaurant on eating behaviours and perceptions of local residents: A natural experiment using repeated cross-sectional data. *Health & Place*. 27. Velempini, E., & Travers, K. D. Accessibility of Nutritious African Foods for an Adequate Diet in Bulawayo, Zimbabwe. *Journal of Nutrition Education*. 28. Aggarwal, A., Cook, A. J., Jiao, J. F., Seguin, R. A., Moudon, A. V., Hurvitz, P. M., & Drewnowski, A. Access to Supermarkets and Fruit and Vegetable Consumption. *American Journal of Public Health*. 29. Ball, K., Crawford, D., & Mishra, G. Socio-economic inequalities in women's fruit and vegetable intakes: a multilevel study of individual, social and environmental mediators. *Public Health Nutr*. 30. Boone-Heinonen, J., Gordon-Larsen, P., Kiefe, C. I., Shikany, J. M., Lewis, C. E., & Popkin, B. M. Fast food restaurants and food stores: longitudinal associations with diet in young to middle-aged adults: the CARDIA study. *Arch Intern Med*. 31. Burgoine, T., Forouhi, N. G., Griffin, S. J., Wareham, N. J., & Monsivais, P. Associations between exposure to takeaway food outlets, takeaway food consumption, and body weight in Cambridgeshire, UK: population based, cross sectional study. *Bmj*. 32. Burgoine, T., Lake, A. A., Stamp, E., Alvanides, S., Mathers, J. C., & Adamson, A. J. Changing foodscapes 1980-2000, using the ASH30 Study. *Appetite*. 33. Burgoine, T., Sarkar, C., Webster, C., & Monsivais, P. Interplay of takeaway food outlet exposure and income on diet and obesity: a cross-sectional study in UK Biobank. *The Lancet*. 34. Caspi, C. E., Kawachi, I., Subramanian, S. V., Adamkiewicz, G., & Sorensen, G. The relationship between diet and perceived and objective access to supermarkets among low-income housing residents. *Soc Sci Med*. 35. Christian, W. J. Using geospatial technologies to explore activity-based retail food environments. *Spat Spatiotemporal Epidemiol*. 36. Chum, A., Farrell, E., Vaivada, T., Labetski, A., Bohnert, A., Selvaratnam, I., . . . O'Campo, P. The effect of food environments on fruit and vegetable intake as modified by time spent at home: a cross-sectional study. *BMJ Open*. 37. Clary, C., Lewis, D. J., Flint, E., Smith, N. R., Kestens, Y., & Cummins, S. The Local Food Environment and Fruit and Vegetable Intake: A Geographically Weighted Regression Approach in the ORiEL Study. *Am J Epidemiol*. 38. Clary, C. M., Ramos, Y., Shareck, M., & Kestens, Y. Should we use absolute or relative measures when assessing foodscape exposure in relation to fruit and vegetable intake? Evidence from a wide-scale Canadian study. *Prev Med*. 39. Curl, C. L., Beresford, S. A., Hajat, A., Kaufman, J. D., Moore, K., Nettleton, J. A., & Diez-Roux, A. V. Associations of organic produce consumption with socioeconomic status and the local food environment: Multi-Ethnic Study of Atherosclerosis (MESA). *PLoS One*. 40. Dubowitz, T., Zenk, S. N., Ghosh-Dastidar, B., Cohen, D. A., Beckman, R., Hunter, G., . . . Collins, R. L. Healthy food access for urban food desert residents: examination of the food environment, food purchasing practices, diet and BMI. *Public Health Nutr*. 41. Greer, S., Schieb, L., Schwartz, G., Onufrak, S., & Park, S. Association of the neighborhood retail food environment with sodium and potassium intake among US adults. *Prev Chronic Dis*. 42. Gustafson, A., Lewis, S., Perkins, S., Wilson, C., Buckner, E., & Vail, A. Neighbourhood and consumer food environment is associated with dietary intake among Supplemental Nutrition Assistance Program (SNAP) participants in Fayette County, Kentucky. *Public Health Nutr*. 43. Gustat, J., O'Malley, K., Luckett, B. G., & Johnson, C. C. Fresh produce consumption and the association between frequency of food shopping, car access, and distance to supermarkets. *Preventive Medicine Reports*. 44. Hattori, A., An, R., & Sturm, R. Neighborhood food outlets, diet, and obesity among California adults, 2007 and 2009. *Prev Chronic Dis*. 45. Hawkesworth, S., Silverwood, R. J., Armstrong, B., Pliakas, T., Nanchahal, K., Sartini, C., . . . Lock, K. Investigating the importance of the local food environment for fruit and vegetable intake in older men and women in 20 UK towns: a cross-sectional analysis of two national cohorts using novel methods. *International Journal of Behavioral Nutrition and Physical Activity*. 46. Jack, D., Neckerman, K., Schwartz-Soicher, O., Lovasi, G. S., Quinn, J., Richards, C., . . . Rundle, A. Socio-economic status, neighbourhood food environments and consumption of fruits and vegetables in New York City. *Public Health Nutr*. 47. Jaime, P. C., Duran, A. C., Sarti, F. M., & Lock, K. Investigating environmental determinants of diet, physical activity, and overweight among adults in Sao Paulo, Brazil. *J Urban Health*. 48. Kruger, D. J., Greenberg, E., Murphy, J. B., DiFazio, L. A., & Youra, K. R. Local concentration of fast-food outlets is associated with poor nutrition and obesity. *Am J Health Promot*. 49. Kyureghian, G., Nayga, R. M., & Bhattacharya, S. The Effect of Food Store Access and Income on Household Purchases of Fruits and Vegetables: A Mixed Effects Analysis. *Applied Economic Perspectives and Policy*. 50. Laraia, B. Proximity of supermarkets is positively associated with diet quality index for pregnancy. *Preventive Medicine*. 51. Laxy, M., Malecki, K. C., Givens, M. L., Walsh, M. C., & Nieto, F. J. The association between neighborhood economic hardship, the retail food environment, fast food intake, and obesity: findings from the Survey of the Health of Wisconsin. *BMC Public Health*. 52. Ledoux, T., Adamus-Leach, H., O'Connor, D. P., Mama, S., & Lee, R. E. The association of binge eating and neighbourhood fast-food restaurant availability on diet and weight status. *Public Health Nutr*. 53. Longacre, M. R., Drake, K. M., MacKenzie, T. A., Gibson, L., Owens, P., Titus, L. J., . . . Dalton, M. A. Fast-food environments and family fast-food intake in nonmetropolitan areas. *Am J Prev Med*. 54. Lucan, S. C., Hillier, A., Schechter, C. B., & Glanz, K. Objective and self-reported factors associated with food-environment perceptions and fruit-and-vegetable consumption: a multilevel analysis. *Prev Chronic Dis*. 55. Lucan, S. C., & Mitra, N. The food environment and dietary intake: Demonstrating a method for GIS-mapping and policy-relevant research. *Journal of Public Health (Germany)*. 56. Macdonald, L., Ellaway, A., Ball, K., & Macintyre, S. Is proximity to a food retail store associated with diet and BMI in Glasgow, Scotland? *BMC Public Health*. 57. Mackenbach, J. D., Burgoine, T., Lakerveld, J., Forouhi, N. G., Griffin, S. J., Wareham, N. J., & Monsivais, P. Accessibility and Affordability of Supermarkets: Associations With the DASH Diet. *American Journal of Preventive Medicine*. 58. Maddock, J. The Relationship between Obesity and the Prevalence of Fast Food Restaurants: State-Level Analysis. *American Journal of Health Promotion*. 59. Mason, K. E., Bentley, R. J., & Kavanagh, A. M. Fruit and vegetable purchasing and the relative density of healthy and unhealthy food stores: evidence from an Australian multilevel study. *J Epidemiol Community Health*. 60. Mejia, N., Lightstone, A. S., Basurto-Davila, R., Morales, D. M., & Sturm, R. Neighborhood Food Environment, Diet, and Obesity Among Los Angeles County Adults, 2011. *Prev Chronic Dis*. 61. Mercille, G., Richard, L., Gauvin, L., Kestens, Y., Shatenstein, B., Daniel, M., & Payette, H. Associations between residential food environment and dietary patterns in urban-dwelling older adults: results from the VoisiNuAge study. *Public Health Nutr*. 62. Mercille, G., Richard, L., Gauvin, L., Kestens, Y., Shatenstein, B., Daniel, M., & Payette, H. The food environment and diet quality of urban-dwelling older women and men: Assessing the moderating role of diet knowledge. *Canadian Journal of Public Health*. 63. Michimi, A., & Wimberly, M. C. Associations of supermarket accessibility with obesity and fruit and vegetable consumption in the conterminous United States. *Int J Health Geogr*. 64. Minaker, L. M., Raine, K. D., Wild, T. C., Nykiforuk, C. I. J., Thompson, M. E., & Frank, L. D. Construct Validation of 4 Food-Environment Assessment Methods: Adapting a Multitrait-Multimethod Matrix Approach for Environmental Measures. *American Journal of Epidemiology*. 65. Moayyed, H., Kelly, B., Feng, X., & Flood, V. Is Living near Healthier Food Stores Associated with Better Food Intake in Regional Australia? *Int J Environ Res Public Health*. 66. Moore, L. V., Diez Roux, A. V., Nettleton, J. A., & Jacobs, D. R., Jr. Associations of the local food environment with diet quality--a comparison of assessments based on surveys and geographic information systems: the multi-ethnic study of atherosclerosis. *Am J Epidemiol*. 67. Moore, L. V., Diez Roux, A. V., Nettleton, J. A., Jacobs, D. R., & Franco, M. Fast-food consumption, diet quality, and neighborhood exposure to fast food: the multi-ethnic study of atherosclerosis. *Am J Epidemiol*. 68. Morland, K., Wing, S., & Roux, A. D. The contextual effect of the local food environment on residents' diets: The atherosclerosis risk in communities study. *American Journal of Public Health*. 69. Murakami, K., Sasaki, S., Takahashi, Y., & Uenishi, K. Neighborhood restaurant availability and frequency of eating out in relation to dietary intake in young Japanese women. *J Nutr Sci Vitaminol (Tokyo)*. 70. Murakami, K., Sasaki, S., Takahashi, Y., Uenishi, K., & Japan Dietetic Students Study, N. No meaningful association of neighborhood food store availability with dietary intake, body mass index, or waist circumference in young Japanese women. *Nutrition Research*. 71. Murakami, K., Sasaki, S., Takahashi, Y., Uenishi, K., & Japan Dietetic Students' Study, N. Neighborhood food store availability in relation to food intake in young Japanese women. *Nutrition*. 72. Oexle, N., Barnes, T. L., Blake, C. E., Bell, B. A., & Liese, A. D. Neighborhood fast food availability and fast food consumption. *Appetite*. 73. Ollberding, N. J., Nigg, C. R., Geller, K. S., Horwath, C. C., Motl, R. W., & Dishman, R. K. Food outlet accessibility and fruit and vegetable consumption. *Am J Health Promot*. 74. Patel, O., Shahulhameed, S., Shivashankar, R., Tayyab, M., Rahman, A., Prabhakaran, D., . . . Jaacks, L. M. Association between full service and fast food restaurant density, dietary intake and overweight/obesity among adults in Delhi, India. *BMC Public Health*. 75. Pearce, J., Hiscock, R., Blakely, T., & Witten, K. A national study of the association between neighbourhood access to fast-food outlets and the diet and weight of local residents. *Health Place*. 76. Pearson, T., Russell, J., Campbell, M. J., & Barker, M. E. Do ‘food deserts’ influence fruit and vegetable consumption?—a cross-sectional study. *Appetite*. 77. Pessoa, M. C., Mendes, L. L., Gomes, C. S., Martins, P. A., & Velasquez-Melendez, G. Food environment and fruit and vegetable intake in a urban population: a multilevel analysis. *BMC Public Health*. 78. Pitts, S. B. J., Wu, Q., McGuirt, J. T., Crawford, T. W., Keyserling, T. C., & Ammerman, A. S. Associations between access to farmers' markets and supermarkets, shopping patterns, fruit and vegetable consumption and health indicators among women of reproductive age in eastern North Carolina, USA. *Public Health Nutrition*. 79. Reitzel, L. R., Okamoto, H., Hernandez, D. C., Regan, S. D., McNeill, L. H., & Obasi, E. M. The Built Food Environment and Dietary Intake among African-American Adults. *Am J Health Behav*. 80. Richardson, A. S., Boone-Heinonen, J., Popkin, B. M., & Gordon-Larsen, P. Neighborhood fast food restaurants and fast food consumption: a national study. *BMC Public Health*. 81. Richardson, A. S., Meyer, K. A., Howard, A. G., Boone-Heinonen, J., Popkin, B. M., Evenson, K. R., . . . Gordon-Larsen, P. Multiple pathways from the neighborhood food environment to increased body mass index through dietary behaviors: A structural equation-based analysis in the CARDIA study. *Health Place*. 82. Robinson, P. L., Dominguez, F., Teklehaimanot, S., Lee, M., Brown, A., & Goodchild, M. Does distance decay modelling of supermarket accessibility predict fruit and vegetable intake by individuals in a large metropolitan area? *J Health Care Poor Underserved*. 83. Rogus, S., Athens, J., Cantor, J., & Elbel, B. Measuring Micro-Level Effects of a New Supermarket: Do Residents Within 0.5 Mile Have Improved Dietary Behaviors? *Journal of the Academy of Nutrition and Dietetics*. 84. Rummo, P. E., Guilkey, D. K., Ng, S. W., Meyer, K. A., Popkin, B. M., Reis, J. P., . . . Gordon-Larsen, P. Understanding bias in relationships between the food environment and diet quality: The Coronary Artery Risk Development in Young Adults (CARDIA) study. *Journal of Epidemiology and Community Health*. 85. Rummo, P. E., Meyer, K. A., Boone-Heinonen, J., Jacobs, D. R., Jr., Kiefe, C. I., Lewis, C. E., . . . Gordon-Larsen, P. Neighborhood availability of convenience stores and diet quality: findings from 20 years of follow-up in the coronary artery risk development in young adults study. *Am J Public Health*. 86. Rummo, P. E., Meyer, K. A., Howard, A. G., Shikany, J. M., Guilkey, D. K., & Gordon-Larsen, P. Fast food price, diet behavior, and cardiometabolic health: Differential associations by neighborhood SES and neighborhood fast food restaurant availability in the CARDIA study. *Health & Place*. 87. Tarlov, E., Zenk, S. N., Matthews, S. A., Powell, L. M., Jones, K. K., Slater, S., & Wing, C. Neighborhood resources to Support healthy diets and physical activity among US military veterans. *Preventing Chronic Disease*. 88. Thornton, L. E., Jeffery, R. W., & Crawford, D. A. Barriers to avoiding fast-food consumption in an environment supportive of unhealthy eating. *Public Health Nutr*. 89. Thornton, L. E., & Kavanagh, A. M. Association between fast food purchasing and the local food environment. *Nutrition and Diabetes*. 90. Thornton, L. E., Lamb, K. E., & Ball, K. Employment status, residential and workplace food environments: associations with women's eating behaviours. *Health Place*. 91. Wang, M. C., Cubbin, C., Ahn, D., & Winkleby, M. A. Changes in neighbourhood food store environment, food behaviour and body mass index, 1981--1990. *Public Health Nutr.* 92. Zenk, S. N., Schulz, A. J., Izumi, B. T., Mentz, G., Israel, B. A., & Lockett, M. Neighborhood food environment role in modifying psychosocial stress-diet relationships. *Appetite.* 93. McInerney, M., Csizmadi, I., Friedenreich, C. M., Uribe, F. A., Nettel-Aguirre, A., McLaren, L., ... & McCormack, G. R. Associations between the neighbourhood food environment, neighbourhood socioeconomic status, and diet quality: An observational study. *BMC public health.* 94. Thornton, L. E., Crawford, D. A., & Ball, K. Neighbourhood-socioeconomic variation in women's diet: the role of nutrition environments. *European journal of clinical nutrition.* | 1. No reported association 2. No reported association 3. No reported association 4. No reported association 5. No reported association 6. No reported association 7. No dietary outcome 8. No dietary outcome 9. No dietary outcome 10. No dietary outcome 11. No dietary outcome 12. No measure of spatial expsoure 13. No measure of spatial expsoure 14. No measure of spatial expsoure 15. No measure of spatial expsoure 16. No measure of spatial expsoure 17. No measure of spatial expsoure 18. No measure of spatial expsoure 19. No measure of spatial expsoure 20. No measure of spatial expsoure 21. No measure of spatial expsoure 22. No measure of spatial expsoure 23. No measure of spatial expsoure 24. No measure of spatial expsoure 25. No measure of spatial expsoure 26. No measure of spatial expsoure 27. No measure of spatial expsoure 28. Only one reported measure of spatial expsoure 29. Only one reported measure of spatial expsoure 30. Only one reported measure of spatial expsoure 31. Only one reported measure of spatial expsoure 32. Only one reported measure of spatial expsoure 33. Only one reported measure of spatial expsoure 34. Only one reported measure of spatial expsoure 35. Only one reported measure of spatial expsoure 36. Only one reported measure of spatial expsoure 37. Only one reported measure of spatial expsoure 38. Only one reported measure of spatial expsoure 39. Only one reported measure of spatial expsoure 40. Only one reported measure of spatial expsoure 41. Only one reported measure of spatial expsoure 42. Only one reported measure of spatial expsoure 43. Only one reported measure of spatial expsoure 44. Only one reported measure of spatial expsoure 45. Only one reported measure of spatial expsoure 46. Only one reported measure of spatial expsoure 47. Only one reported measure of spatial expsoure 48. Only one reported measure of spatial expsoure 49. Only one reported measure of spatial expsoure 50. Only one reported measure of spatial expsoure 51. Only one reported measure of spatial expsoure 52. Only one reported measure of spatial expsoure 53. Only one reported measure of spatial expsoure 54. Only one reported measure of spatial expsoure 55. Only one reported measure of spatial expsoure 56. Only one reported measure of spatial expsoure 57. Only one reported measure of spatial expsoure 58. Only one reported measure of spatial expsoure 59. Only one reported measure of spatial expsoure 60. Only one reported measure of spatial expsoure 61. Only one reported measure of spatial expsoure 62. Only one reported measure of spatial expsoure 63. Only one reported measure of spatial expsoure 64. Only one reported measure of spatial expsoure 65. Only one reported measure of spatial expsoure 66. Only one reported measure of spatial expsoure 67. Only one reported measure of spatial expsoure 68. Only one reported measure of spatial expsoure 69. Only one reported measure of spatial expsoure 70. Only one reported measure of spatial expsoure 71. Only one reported measure of spatial expsoure 72. Only one reported measure of spatial expsoure 73. Only one reported measure of spatial expsoure 74. Only one reported measure of spatial expsoure 75. Only one reported measure of spatial expsoure 76. Only one reported measure of spatial expsoure 77. Only one reported measure of spatial expsoure 78. Only one reported measure of spatial expsoure 79. Only one reported measure of spatial expsoure 80. Only one reported measure of spatial expsoure 81. Only one reported measure of spatial expsoure 82. Only one reported measure of spatial expsoure 83. Only one reported measure of spatial expsoure 84. Only one reported measure of spatial expsoure 85. Only one reported measure of spatial expsoure 86. Only one reported measure of spatial expsoure 87. Only one reported measure of spatial expsoure 88. Only one reported measure of spatial expsoure 89. Only one reported measure of spatial expsoure 90. Only one reported measure of spatial expsoure 91. Only one reported measure of spatial expsoure 92. Only one reported measure of spatial exposure 93. Only one reported measure of spatial exposure 94. Only one reported measure of spatial expsoure |

| **Citations excluded from cited searches based on full text review** | **Reason** |
| --- | --- |
| 1. Aggarwal A, Cook AJ, Jiao J, Seguin RA, Vernez Moudon A, Hurvitz PM, Drewnowski A. Access to supermarkets and fruit and vegetable consumption. American journal of public health. 2014 May;104(5):917-23. 2. Alber JM, Green SH, Glanz K. Perceived and Observed Food Environments, Eating Behaviors, and BMI. American journal of preventive medicine. 2018 Jan 12. 3. Ball K, Crawford D, Timperio A, Salmon J. Eating Behaviours and the Food Environment. Obesogenic Environments. 2010 Jan 1:149-63. 4. Bodor JN, Hutchinson PL, Rose D. Car ownership and the association between fruit and vegetable availability and diet. Preventive medicine. 2013 Dec 1;57(6):903-5. 5. Boone-Heinonen J, Diez-Roux AV, Goff DC, Loria CM, Kiefe CI, Popkin BM, Gordon-Larsen P. The neighborhood energy balance equation: does neighborhood food retail environment+ physical activity environment= obesity? The CARDIA study. PLoS One. 2013 Dec 27;8(12):e85141. 6. Boone-Heinonen J, Gordon-Larsen P, Kiefe CI, Shikany JM, Lewis CE, Popkin BM. Fast food restaurants and food stores: longitudinal associations with diet in young to middle-aged adults: the CARDIA study. Archives of internal medicine. 2011 Jul 11;171(13):1162-70. 7. Burgoine T, Forouhi NG, Griffin SJ, Brage S, Wareham NJ, Monsivais P. Does neighborhood fast-food outlet exposure amplify inequalities in diet and obesity? A cross-sectional study, 2. The American journal of clinical nutrition. 2016 May 11;103(6):1540-7. 8. Caspi CE, Kawachi I, Subramanian SV, Adamkiewicz G, Sorensen G. The relationship between diet and perceived and objective access to supermarkets among low-income housing residents. Social science & medicine. 2012 Oct 1;75(7):1254-62. 9. Clary C, Lewis DJ, Flint E, Smith NR, Kestens Y, Cummins S. The local food environment and fruit and vegetable intake: a geographically weighted regression approach in the ORiEL Study. American journal of epidemiology. 2016 Dec 1;184(11):837-46. 10. Clary CM, Ramos Y, Shareck M, Kestens Y. Should we use absolute or relative measures when assessing foodscape exposure in relation to fruit and vegetable intake? Evidence from a wide-scale Canadian study. Preventive medicine. 2015 Feb 1;71:83-7. 11. D'Angelo H, Suratkar S, Song HJ, Stauffer E, Gittelsohn J. Access to food source and food source use are associated with healthy and unhealthy food-purchasing behaviours among low-income African-American adults in Baltimore City. Public health nutrition. 2011 Sep;14(9):1632-9. 12. Dubowitz T, Zenk SN, Ghosh-Dastidar B, Cohen DA, Beckman R, Hunter G, Steiner ED, Collins RL. Healthy food access for urban food desert residents: examination of the food environment, food purchasing practices, diet and BMI. Public health nutrition. 2015 Aug;18(12):2220-30. 13. Franco M, Diez-Roux AV, Nettleton JA, Lazo M, Brancati F, Caballero B, Glass T, Moore LV. Availability of healthy foods and dietary patterns: the Multi-Ethnic Study of Atherosclerosis–. The American journal of clinical nutrition. 2009 Jan 14;89(3):897-904. 14. Hawkesworth S, Silverwood RJ, Armstrong B, Pliakas T, Nanchahal K, Sartini C, Amuzu A, Wannamethee G, Atkins J, Ramsay SE, Casas JP. Investigating the importance of the local food environment for fruit and vegetable intake in older men and women in 20 UK towns: a cross-sectional analysis of two national cohorts using novel methods. International Journal of Behavioral Nutrition and Physical Activity. 2017 Dec;14(1):128. 15. Jack D, Neckerman K, Schwartz-Soicher O, Lovasi GS, Quinn J, Richards C, Bader M, Weiss C, Konty K, Arno P, Viola D. Socio-economic status, neighbourhood food environments and consumption of fruits and vegetables in New York City. Public health nutrition. 2013 Jul;16(7):1197-205. 16. Izumi BT, Zenk SN, Schulz AJ, Mentz GB, Wilson C. Associations between neighborhood availability and individual consumption of dark-green and orange vegetables among ethnically diverse adults in Detroit. Journal of the Academy of Nutrition and Dietetics. 2011 Feb 1;111(2):274-9. 17. Jaime PC, Duran AC, Sarti FM, Lock K. Investigating environmental determinants of diet, physical activity, and overweight among adults in Sao Paulo, Brazil. Journal of urban health. 2011 Jun 1;88(3):567-81. 18. Jiao J, Moudon AV, Kim SY, Hurvitz PM, Drewnowski A. Health implications of adults’ eating at and living near fast food or quick service restaurants. Nutrition & diabetes. 2015 Jul;5(7):e171. 19. Kyureghian G, Nayga Jr RM, Bhattacharya S. The effect of food store access and income on household purchases of fruits and vegetables: A mixed effects analysis. Applied Economic Perspectives and Policy. 2012 Nov 27;35(1):69-88. 20. Laska MN, Graham DJ, Moe SG, Van Riper D. Young adult eating and food-purchasing patterns: food store location and residential proximity. American journal of preventive medicine. 2010 Nov 1;39(5):464-7. 21. Ledoux T, Adamus-Leach H, O'Connor DP, Mama S, Lee RE. The association of binge eating and neighbourhood fast-food restaurant availability on diet and weight status. Public health nutrition. 2015 Feb;18(2):352-60. 22. Liese AD, Bell BA, Barnes TL, Colabianchi N, Hibbert JD, Blake CE, Freedman DA. Environmental influences on fruit and vegetable intake: results from a path analytic model. Public health nutrition. 2014 Nov;17(11):2595-604. 23. Lind PL, Jensen PV, Glümer C, Toft U. The association between accessibility of local convenience stores and unhealthy diet. The European Journal of Public Health. 2016 Feb 6;26(4):634-9. 24. Longacre MR, Drake KM, MacKenzie TA, Gibson L, Owens P, Titus LJ, Beach ML, Dalton MA. Fast-food environments and family fast-food intake in nonmetropolitan areas. American journal of preventive medicine. 2012 Jun 1;42(6):579-87. 25. Lucan SC, Mitra N. The food environment and dietary intake: demonstrating a method for GIS-mapping and policy-relevant research. Journal of Public Health. 2012 Aug 1;20(4):375-85. 26. Macdonald L, Ellaway A, Ball K, Macintyre S. Is proximity to a food retail store associated with diet and BMI in Glasgow, Scotland?. BMC public health. 2011 Dec;11(1):464. 27. Mackenbach JD, Burgoine T, Lakerveld J, Forouhi NG, Griffin SJ, Wareham NJ, Monsivais P. Accessibility and affordability of supermarkets: associations with the DASH diet. American journal of preventive medicine. 2017 Jul 1;53(1):55-62. 28. Mason KE, Bentley RJ, Kavanagh AM. Fruit and vegetable purchasing and the relative density of healthy and unhealthy food stores: evidence from an Australian multilevel study. J Epidemiol Community Health. 2012 Oct 1:jech-2012. 29. McInerney M, Csizmadi I, Friedenreich CM, Uribe FA, Nettel-Aguirre A, McLaren L, Potestio M, Sandalack B, McCormack GR. Associations between the neighbourhood food environment, neighbourhood socioeconomic status, and diet quality: An observational study. BMC public health. 2016 Dec;16(1):984. 30. Mercille G, Richard L, Gauvin L, Kestens Y, Shatenstein B, Daniel M, Payette H. Associations between residential food environment and dietary patterns in urban-dwelling older adults: results from the VoisiNuAge study. Public health nutrition. 2012 Nov;15(11):2026-39. 31. Moayyed H, Kelly B, Feng X, Flood V. Is living near healthier food stores associated with better food intake in regional Australia?. International journal of environmental research and public health. 2017 Aug 7;14(8):884. 32. Murakami K, Sasaki S, Takahashi Y, Uenishi K. No meaningful association of neighborhood food store availability with dietary intake, body mass index, or waist circumference in young Japanese women. Nutrition research. 2010 Aug 1;30(8):565-73. 33. Moore LV, Diez Roux AV, Nettleton JA, Jacobs DR, Franco M. Fast-food consumption, diet quality, and neighborhood exposure to fast food: the multi-ethnic study of atherosclerosis. American journal of epidemiology. 2009 May 8;170(1):29-36. 34. Murakami K, Sasaki S, Takahashi Y, Uenishi K. Neighborhood food store availability in relation to food intake in young Japanese women. Nutrition. 2009 Jun 1;25(6):640-6. 35. Murakami K, Sasaki S, Takahashi Y, Uenishi K. Neighborhood restaurant availability and frequency of eating out in relation to dietary intake in young Japanese women. Journal of nutritional science and vitaminology. 2011;57(1):87-94. 36. Oexle N, Barnes TL, Blake CE, Bell BA, Liese AD. Neighborhood fast food availability and fast food consumption. Appetite. 2015 Sep 1;92:227-32. 37. Ollberding NJ, Nigg CR, Geller KS, Horwath CC, Motl RW, Dishman RK. Food outlet accessibility and fruit and vegetable consumption. American Journal of Health Promotion. 2012 Jul;26(6):366-70. 38. Pessoa MC, Loures Mendes L, Teixeira Caiaffa W, Carvalho Malta D, Velásquez-Meléndez G. Availability of food stores and consumption of fruit, legumes and vegetables in a Brazilian urban area. Nutricion hospitalaria. 2015;31(3). 39. Pessoa MC, Mendes LL, Gomes CS, Martins PA, Velasquez-Melendez G. Food environment and fruit and vegetable intake in a urban population: a multilevel analysis. BMC Public Health. 2015 Dec;15(1):1012. 40. Reitzel LR, Okamoto H, Hernandez DC, Regan SD, McNeill LH, Obasi EM. The built food environment and dietary intake among African-American adults. American journal of health behavior. 2016 Jan 1;40(1):3-11. 41. Richardson AS, Boone-Heinonen J, Popkin BM, Gordon-Larsen P. Neighborhood fast food restaurants and fast food consumption: a national study. BMC public health. 2011 Dec;11(1):543. 42. Rummo PE, Guilkey DK, Ng SW, Meyer KA, Popkin BM, Reis JP, Shikany JM, Gordon-Larsen P. Understanding bias in relationships between the food environment and diet quality: the Coronary Artery Risk Development in Young Adults (CARDIA) study. J Epidemiol Community Health. 2017 Dec 1;71(12):1185-90. 43. Rummo PE, Meyer KA, Boone-Heinonen J, Jacobs Jr DR, Kiefe CI, Lewis CE, Steffen LM, Gordon-Larsen P. Neighborhood availability of convenience stores and diet quality: findings from 20 years of follow-up in the coronary artery risk development in young adults study. American journal of public health. 2015 May;105(5):e65-73. 44. Sharkey JR, Johnson CM, Dean WR. Food access and perceptions of the community and household food environment as correlates of fruit and vegetable intake among rural seniors. BMC geriatrics. 2010 Dec;10(1):32. 45. Williams LK, Thornton L, Crawford D. Optimising women’s diets. An examination of factors that promote healthy eating and reduce the likelihood of unhealthy eating. Appetite. 2012 Aug 1;59(1):41-6. 46. Yamashita T, Kunkel SR. Geographic access to healthy and unhealthy foods for the older population in a US metropolitan area. Journal of Applied Gerontology. 2012 Jun;31(3):287-313. | 1. Only one reported measure of spatial exposure 2. No measure of spatial exposure 3. No measure of spatial exposure 4. No measure of spatial exposure 5. Only one reported measure of spatial exposure 6. Only one reported measure of spatial exposure 7. Only one reported measure of spatial exposure 8. Only one reported measure of spatial exposure 9. Only one reported measure of spatial exposure 10. Only one reported measure of spatial exposure 11. No measure of spatial exposure 12. Only one reported measure of spatial exposure 13. Only one reported measure of spatial exposure 14. Only one reported measure of spatial exposure 15. Only one reported measure of spatial exposure 16. Only one reported measure of spatial exposure 17. Only one reported measure of spatial exposure 18. No measure of spatial exposure 19. Only one reported measure of spatial exposure 20. Only one reported measure of spatial exposure 21. Only one reported measure of spatial exposure 22. No reported association 23. Only one reported measure of spatial exposure 24. Only one reported measure of spatial exposure 25. Only one reported measure of spatial exposure 26. Only one reported measure of spatial exposure 27. Only one reported measure of spatial exposure 28. Only one reported measure of spatial exposure 29. Only one reported measure of spatial exposure 30. Only one reported measure of spatial exposure 31. Only one reported measure of spatial exposure 32. Only one reported measure of spatial exposure 33. Only one reported measure of spatial exposure 34. Only one reported measure of spatial exposure 35. Only one reported measure of spatial exposure 36. Only one reported measure of spatial exposure 37. Only one reported measure of spatial exposure 38. No reported association 39. Only one reported measure of spatial exposure 40. Only one reported measure of spatial exposure 41. Only one reported measure of spatial exposure 42. Only one reported measure of spatial exposure 43. Only one reported measure of spatial exposure 44. Only one reported measure of spatial exposure 45. No measure of spatial exposure 46. Only one reported measure of spatial exposure |
