# Supplementary material for: Food environments and dietary intakes among adults: does the type of spatial exposure measurement matter? A systematic review
Source: Int J Health Geogr. 2018 Jun 9;17:19. doi: 10.1186/s12942-018-0139-7 (PMC5994245; doi:10.1186/s12942-018-0139-7)
Supplement: Supplementary file 3 — Additional file 3. Study quality scores. Criteria and scoring system used to determine study quality. [file 12942_2018_139_MOESM3_ESM.docx]

**Additional file 3. Study quality scores.**

| **Author (date)** | **Data aggregation** | **Food outlet data source** | **Food outlet validation** | **Food outlet classification** | **Study design** | **Dietary assessment** | **Response fraction** | **Data analysis** |
| --- | --- | --- | --- | --- | --- | --- | --- | --- |
| Athens (2016) | 1 | 1 | 0 | 2 | 2 | 1 | 1 | 2 |
| Bernsdorf (2017) | 1 | 3 | 2 | 2 | 2 | 1 | 2 | 2 |
| Bodor (2008) | 1 | 1 | 2 | 2 | 2 | 0 | 2 | 2 |
| Dunn (2012) | 1 | 3 | 2 | 2 | 2 | 1 | 3 | 2 |
| Duran (2016) | 1 | 3 | 2 | 2 | 2 | 2 | 0 | 1 |
| Layte (2011) | 1 | 0 | 0 | 0 | 0 | 2 | 3 | 2 |
| LeDoux (2014) | 1 | 2 | 0 | 2 | 0 | 1 | 1 | 2 |
| Minaker (2013) | 1 | 1 | 2 | 1 | 2 | 2 | 2 | 2 |
| Sharkey (2011) | 1 | 3 | 2 | 2 | 2 | 1 | 3 | 2 |
| Thornton (2009) | 1 | 1 | 0 | 2 | 2 | 1 | 2 | 2 |
| Thornton (2012) | 0 | 2 | 1 | 2 | 1 | 1 | 2 | 1 |
| Turrell (2008) | 0 | 1 | 2 | 2 | 2 | 1 | 2 | 2 |
| Williams (2010) | 1 | 2 | 0 | 1 | 0 | 1 | 2 | 1 |
| Zenk (2009) | 0 | 2 | 2 | 1 | 2 | 1 | 2 | 2 |
| **MAX SCORE** | **1** | **3** | **2** | **2** | **2** | **2** | **3** | **2** |
