# Supplementary material for: Food environments and dietary intakes among adults: does the type of spatial exposure measurement matter? A systematic review
Source: Int J Health Geogr. 2018 Jun 9;17:19. doi: 10.1186/s12942-018-0139-7 (PMC5994245; doi:10.1186/s12942-018-0139-7)
Supplement: Supplementary file 4 — Additional file 4a. Summary of associations with fruit, vegetable, and fruit and vegetable intake by food outlet type *. b. Summary of associations with fast food and unhealthy food intake by food outlet type *. c. Summary of associations with takeaway purchase and fast food purchase by food outlet type *. d. Summary of associations with diet quality by food outlet type*. Summary of extracted effect sizes and p values for associations between food outlet exposure and dietary outcomes. [file 12942_2018_139_MOESM4_ESM.docx]

**Additional file 4a. Summary of associations with fruit, vegetable, and fruit and vegetable intake by food outlet type *.**

| **Author (date)** | **Outcome** | **Food outlet** | **GIS Method** | **Effect size (precision)** | **p-value** |
| --- | --- | --- | --- | --- | --- |
| Bodor (2008)^1^ | Fruit | Supermarkets | Presence 1 km Euclidean buffer  **Proximity Euclidean distance** | -0.123 (0.518)  **0.445** (0.955) | 0.813  0.642 |
| Williams (2010)^2^ | Fruit | Supermarkets | **Count 2 km network buffer**  Proximity network distance | **0.049** (-0.09, 0.18)  0.000 (-0.24, 0.27) | 0.54  0.95 |
| Thornton (2012)^3^ | Fruit | Supermarkets | Proximity network distance  **Presence 0.4 km network buffer**  Presence 0.4 km Euclidean buffer  Density 0.4 km Euclidean kernel  Count 0.4 km Euclidean buffer  Count 0.4 km network buffer  Presence 1 km network buffer  Presence 1 km Euclidean buffer  Density 1 km Euclidean kernel  Count 1 km Euclidean buffer  Count 1 km network buffer  Count 2 km Euclidean buffer  Count 2 km network buffer  Density 2 km Euclidean kernel  Count 3 km Euclidean buffer  Count 3 km network buffer  Density 3 km Euclidean kernel  Count 4 km Euclidean buffer  Count 4 km network buffer  Density 4 km Euclidean kernel  Count 5 km Euclidean buffer  Count 5 km network buffer  Density 5 km Euclidean kernel | ­0.043 (0.063)  **0.351** (0.201)  ­0.031 (0.144)  0.033 (0.060)  0.002 (0.059)  0.050 (0.056)  0.067 (0.123)  0.034 (0.137)  0.086 (0.060)  0.184 (0.058)  0.073 (0.059)  0.218 (0.059)  0.293 (0.062)  0.213 (0.056)  0.126 (0.060)  0.230 (0.059)  0.222 (0.058)  0.077 (0.062)  0.143 (0.056)  0.189 (0.061)  0.072 (0.063)  0.072 (0.064)  0.153 (0.125) | 0.503  0.081  0.831  0.587  0.969  0.369  0.587  0.805  0.153  ***0.001***  0.223  ***< 0.001***  ***< 0.001***  ***< 0.001***  ***0.034***  ***< 0.001***  ***< 0.001***  0.19  ***0.017***  ***0.002***  0.284  0.268  ***0.016*** |
| Williams (2010)^2^ | Fruit | Fruit and vegetable stores | Count 2 km network buffer  **Proximity network distance** | 0.030 (-0.07, 0.12)  **-0.105** (-0.25, 0.04) | 0.60  0.15 |
| Bodor (2008)^1^ | Fruit | Small stores | Presence 100 m Euclidean buffer  **Proximity Euclidean distance** | 0.632 (0.369)  **-4.551** (3.532) | 0.090  0.201 |
| Bodor (2008)^1^ | Vegetables | Supermarkets | Presence 1 km Euclidean buffer  **Proximity Euclidean distance** | -0.075 (0.575)  **-0.094** (1.060) | 0.897  0.930 |
| Williams (2010)^2^ | Vegetables | Supermarkets | Count 2 km network buffer  **Proximity network distance** | 0.086 (-0.06, 0.24)  **-0.151** (-0.45, 0.14) | 0.25  0.31 |
| Thornton (2012)^3^ | Vegetables | Supermarkets | Proximity network distance  Presence 0.4 km Euclidean buffer  Presence 0.4 km network buffer  Count 0.4 km Euclidean buffer  Count 0.4 km network buffer  Density 0.4 km Euclidean kernel  Presence 1 km Euclidean buffer  Presence 1 km network buffer  Count 1 km Euclidean buffer  Count 1 km network buffer  Density 1 km Euclidean kernel  Count 2 km Euclidean buffer  **Count 2 km network buffer**  Density 2 km Euclidean kernel  Count 3 km Euclidean buffer  Count 3 km network buffer  Density 3 km Euclidean kernel  Count 4 km Euclidean buffer  Count 4 km network buffer  Density 4 km Euclidean kernel  Count 5 km Euclidean buffer  Count 5 km network buffer  Density 5 km Euclidean kernel | 0.032 (0.068)  ­0.117 (0.150)  0.124 (0.206)  ­0.047 (0.061)  0.015 (0.057)  ­0.055 (0.063)  ­0.093 (0.142)  0.083 (0.516)  0.181 (0.061)  0.025 (0.061)  0.048 (0.063)  0.222 (0.062)  **0.296** (0.068)  0.199 (0.060)  0.120 (0.066)  0.217 (0.064)  0.226 (0.062)  0.015 (0.070)  0.106 (0.062)  0.181 (0.065)  ­0.009 (0.063)  0.016 (0.064)  0.119 (0.070) | 0.639  0.437  0.548  0.444  0.790  0.376  0.514  0.516  ***0.003***  0.687  0.445  ***< 0.001***  ***< 0.001***  ***0.001***  0.063  ***< 0.001***  ***< 0.001***  0.840  0.100  ***0.006***  0.898  0.835  0.082 |
| Bodor (2008)^1^ | Vegetables | Small stores | Presence 100 m Euclidean buffer  **Proximity Euclidean distance**  ­ | 0.515 (0.412)  **-3.187** (3.938) | 0.214  0.420 |
| Williams (2010)^2^ | Vegetables | Fruit and vegetable stores | Count 2 km network buffer  **Proximity network distance** | 0.0295 (-0.07, 0.13)  **-0.072** (-0.23, 0.09) | 0.61  0.37 |
| Thornton (2012)^3^ | Fruit and vegetables | Supermarkets | Proximity network distance  Presence 0.4 km Euclidean buffer  Presence 0.4 km network buffer  Count 0.4 km Euclidean buffer  Count 0.4 km network buffer  Density 0.4 km Euclidean kernel  Presence 1 km Euclidean buffer  Presence 1 km network buffer  Count 1 km Euclidean buffer  Count 1 km network buffer  Density 1 km Euclidean kernel  Count 2 km Euclidean buffer  **Count 2 km network buffer**  Density 2 km Euclidean kernel  Count 3 km Euclidean buffer  Count 3 km network buffer  Density 3 km Euclidean kernel  Count 4 km Euclidean buffer  Count 4 km network buffer  Density 4 km Euclidean kernel  Count 5 km Euclidean buffer  Count 5 km network buffer  Density 5 km Euclidean kernel | ­0.022 (0.118)  ­0.122 (0.264)  0.469 (0.359)  ­0.034 (0.107)  0.065 (0.099)  ­0.014 (0.111)  ­0.056 (0.250)  0.184 (0.224)  0.367 (0.106)  0.112 (0.108)  0.146 (0.111)  0.443 (0.104)  **0.601** (0.113)  0.421 (0.103)  0.257 (0.109)  0.455 (0.110)  0.457 (0.107)  0.108 (0.116)  0.261 (0.112)  0.383 (0.113)  0.072 (0.116)  0.104 (0.120)  0.287 (0.119) | 0.851  0.646  0.192  0.755  0.512  0.896  0.824  0.410  ***0.001***  0.295  0.187  ***< 0.001***  ***< 0.001***  ***< 0.001***  ***0.022***  ***< 0.001***  ***< 0.001***  0.347  ***0.019***  ***0.001***  0.547  0.391  ***0.016*** |
| Zenk (2009)^3^ | Fruit and vegetables | Supermarkets and grocery stores | **Presence 0.5 mile Euclidean buffer**  Proximity network distance | **0.691** (0.21)  0.004 (0.008) | ***0.002***  0.638 |
| Duran (2016)^2^ | Fruit and vegetables | Supermarkets + grocery stores + fruit and vegetables stores | Density 1.6 km Euclidean buffer/10,000 pop. (Q1)  Density 1.6 km Euclidean buffer/10,000 pop. (Q2)  Density 1.6 km Euclidean buffer/10,000 pop. (Q3)  Proximity Euclidean distance (Q1)  Proximity Euclidean distance (Q2)  **Proximity Euclidean distance (Q3)** | -0.223 (-0.446, -0.010)  -0.186 (-0.462, 0.077)  -0.174 (-0.431, 0.077  0.255 (0.113, 0.399)  0.166 (0.020, 0.315)  **0.278** (0.148, 0.405) | ***< 0.05***  > 0.05  > 0.05  ***< 0.05***  ***< 0.05***  ***< 0.05*** |
| LeDoux (2014)^4^ | Fruit and vegetables | Supermarkets | **Count 0.25 mile network buffer**  Count 0.5 mile network buffer  Proximity network distance | **0.428** (0.193)  0.162 (0.124)  -3.230E-04 (1.790E-04) | ***< 0.05***  > 0.05  > 0.05 |
|  |  | Convenience stores | Count 0.25 mile network buffer  **Count 0.5 mile network buffer**  Proximity network distance | -0.032 (0.060)  **0.049** (0.030)  1.860E-04 (3.120E-04) | > 0.05  > 0.05  > 0.05 |
|  |  | Fast food | **Count 0.25 mile network buffer**  Count 0.5 mile network buffer  Proximity network distance | **-0.533** (0.242)  -0.135 (0.054)  3.420E-04 (1.670E-04) | ***< 0.05***  ***< 0.05***  ***< 0.05*** |

*Largest effect size per study in **bold**. Significant p-values in the unexpected direction in **bold** and expected direction in ***bold italics***. ^1^ Unstandardised β coef. (SEM). ^2^ Unstandardised β coef. (95% CI). ^3^ Standardised β coef. (SE). ^4^ Unstandardised β coef. (SE).

**Additional file 4b. Summary of associations with fast food and unhealthy food intake by food outlet type *.**

| **Author (date)** | **Outcome** | **Food outlet** | **GIS Method** | **Effect size (precision)** | **p-value** |
| --- | --- | --- | --- | --- | --- |
| Athens (2016)^5^ | Fast food | Fast food outlets | Count 0.25 mile network buffer  **Presence 0.25 mile network buffer**  Count 0.5 mile network buffer  Presence 0.5 mile network buffer  Count 1 mile network buffer  Presence 1 mile network buffer  Proximity network distance | 0.054 (-0.019, 0.128)  **0.123** (-0.075, 0.321)  ­-0.031 -0.114, 0.053)  -0.080 (-0.241, 0.082)  ­-0.036 (-0.134, 0.063)  ­-0.069 (-0.364, 0.224)  0.011 (-0.063, 0.084) | > 0.05  > 0.05  > 0.05  > 0.05  > 0.05  > 0.05  > 0.05 |
| Bernsdorf (2017)^2^ | Fast food | Fast food outlets | Count 1 km network buffer (1)  Count 1 km network buffer (2)  Count 1 km network buffer (3-5)  **Count 1 km network buffer (6-10)**  Count 1 km network buffer (11+)  Proximity network distance < 1 km  Proximity network distance 1-4 km  Proximity network distance > 4 km | 0.166 (0.049, 0.293)  0.215 (0.086, 0.344)  0.262 (0.148, 0.372)  **0.322** (0.199, 0.438)  0.315 (0.182, 0.451)  -0.030 (-0.041, -0.020)  -0.083 (-0.163, -0.010)  0.049 (0.030, 0.077) | ***< 0.05***  ***< 0.05***  ***< 0.05***  ***< 0.05***  ***< 0.05***  ***< 0.05***  ***< 0.05***  **< 0.05** |
| Dunn (2012)^3^ | Fast food | Fast food outlets | Count 1 mile buffer  **Count 3 mile buffer**  Proximity | ­-0.044 (0.591)  **­-0.046** (0.661)  0.024 (0.230) | > 0.05  > 0.05  > 0.05 |
| Sharkey (2011)^6^ | Fast food | Traditional fast food outlets | **Proximity network distance**  Count 1 mile network buffer  Count 3 mile network buffer  Count 5 mile network buffer | **-0.243** (not reported)  0.182 (not reported)  0.148 (not reported)  0.186 (not reported) | ***< 0.001***  ***< 0.01***  ***< 0.01***  ***< 0.001*** |
| Sharkey (2011)^6^ | Fast food | Traditional and non-traditional fast food outlets | Proximity network distance  Count 1 mile network buffer  Count 3 mile network buffer  **Count 5 mile network buffer** | -0.163 (not reported)  0.170 (not reported)  0.137 (not reported)  **0.185** (not reported) | ***< 0.01***  ***< 0.01***  ***< 0.01***  ***< 0.01*** |
| Sharkey (2011)^6^ | Fast food | Non-traditional fast food outlets | Proximity network distance  Count 1 mile network buffer  Count 3 mile network buffer  **Count 5 mile network buffer** | -0.154 (not reported)  0.147 (not reported)  0.128 (not reported)  **0.178** (not reported) | ***< 0.01***  ***< 0.01***  ***< 0.05***  ***< 0.01*** |
| Athens (2016)^5^ | Fast food | Supermarkets | Count 0.25 mile network buffer  Presence 0.25 mile network buffer  Count 0.5 mile network buffer  Presence 0.5 mile network buffer  Count 1 mile network buffer  **Presence 1 mile network buffer**  Proximity network distance | ­-0.108 (-0.199, -0.017)  -0.197 (-0.409, 0.015)  ­-0.088 (-0.170, -0.006)  -0.168 (-0.331, -0.004)  ­-0.072 (-0.171, 0.030)  ­**-0.214** (-0.467, 0.037)  0.100 (0.017, 0.183) | ***< 0.05***  > 0.05  ***< 0.05***  ***< 0.05***  > 0.05  > 0.05  ***< 0.05*** |
| Duran (2016)^2^ | Sugar sweetened drinks | Supermarkets + grocery stores + fruit and vegetables stores | Density 1.6 km Euclidean buffer/10,000 pop. (Q1)  Density 1.6 km Euclidean buffer/10,000 pop. (Q2)  **Density 1.6 km Euclidean buffer/10,000 pop. (Q3)**  Proximity Euclidean distance (Q1)  Proximity Euclidean distance (Q2)  Proximity Euclidean distance (Q3) | -0.094 (-0.371, 0.174)  0.058 (-0.211, 0.329)  **0.157** (-0.163, 0.470)  -0.010 (-0.211, 0.199)  0.039 (-0.139, 0.223)  0.039 (-0.163, 0.231) | > 0.05  > 0.05  > 0.05  > 0.05  > 0.05  > 0.05 |
| LeDoux (2014)^4^ | Soda and juice | Supermarkets | **Count 0.25 mile network buffer**  Count 0.5 mile network buffer  Proximity network distance | **0.599** (0.305)  0.123 (0.164)  -1.530E-04 (2.710E-04) | ***< 0.05***  > 0.05  > 0.05 |
| LeDoux (2014)^4^ | Soda and juice | Convenience stores | Count 0.25 mile network buffer  **Count 0.5 mile network buffer**  Proximity network distance | -0.004 (0.077)  **-0.011** (0.035)  -8.300E-05 (4.410E-04) | > 0.05  > 0.05  > 0.05 |
| LeDoux (2014)^4^ | Soda and juice | Fast food outlets | **Count 0.25 mile network buffer**  Count 0.5 mile network buffer  Proximity network distance | **-0.336** (-0.336)  -0.090 (0.066)  4.040E-04 (2.310E-04) | > 0.05  > 0.05  > 0.05 |
| LeDoux (2014)^4^ | Sweet and salty snacks | Supermarkets | **Count 0.25 mile network buffer**  Count 0.5 mile network buffer  Proximity network distance | **0.957** (0.311)  0.029 (0.163)  -2.950E-04 (2.980E-04) | ***< 0.05***  > 0.05  > 0.05 |
| LeDoux (2014)^4^ | Sweet and salty snacks | Convenience stores | **Count 0.25 mile network buffer**  Count 0.5 mile network buffer  Proximity network distance | **-0.119** (0.064)  -0.035 (0.032)  2.940E-04 (4.070E-04) | > 0.05  > 0.05  > 0.05 |
| LeDoux (2014)^4^ | Sweet and salty snacks | Fast food outlets | **Count 0.25 mile network buffer**  Count 0.5 mile network buffer  Proximity network distance | **-0.113** (0.302)  -0.092 (0.067)  3.330E-04 (2.210E-04) | > 0.05  > 0.05  > 0.05 |

*Largest effect size per study in **bold**. Significant p-values in the unexpected direction in **bold** and expected direction in ***bold italics***. ^2^ Unstandardised β coef. (95% CI), ^3^ Standardised β coef. (SE), ^4^ Unstandardised β coef. (SE), ^5^ Standardised β coef. (95% CI), ^6^ Standardised β coef. (not reported).

**Additional file 4c. Summary of associations with takeaway purchase and fast food purchase by food outlet type *.**

| **Author (date)** | **Outcome** | **Food outlet** | **GIS Method** | **Effect size (precision)** | **p-value** |
| --- | --- | --- | --- | --- | --- |
| Turrell (2008)^4^ | Takeaway purchase | Sweet food takeaway outlets | Density 2.5 km Euclidean buffer/10 000 pop.  **Average proximity network distance**  Proximity network distance | 0.019 (0.029)  **0.078** (0.038)  0.089 (0.048) | > 0.05  **0.041**  > 0.05 |
|  |  | Healthy takeaway outlets | Density 2.5 km Euclidean buffer/10 000 pop.  **Average proximity network distance**  Proximity network distance | 0.01 (0.015)  **0.038** (0.037)  0.037 (0.042) | > 0.05  > 0.05  > 0.05 |
|  |  | Café/coffee shops | Density 2.5 km Euclidean buffer/10 000 pop.  **Average proximity network distance**  Proximity network distance | 0.003 (0.011)  **0.100** (0.048)  0.098 (0.055) | > 0.05  **0.039**  > 0.05 |
|  |  | Other ethnic takeaway outlets | Density 2.5 km Euclidean buffer/10 000 pop.  **Average proximity network distance**  Proximity network distance | 0.023 (0.018)  **0.058** (0.041)  0.049 (0.046) | > 0.05  > 0.05  > 0.05 |
|  |  | Asian takeaway outlets | Density 2.5 km Euclidean buffer/10 000 pop.  **Average proximity network distance**  Proximity network distance | 0.011 (0.008)  **0.088** (0.06)  0.051 (0.051) | > 0.05  > 0.05  > 0.05 |
|  |  | General independent takeaway outlets | Density 2.5 km Euclidean buffer/10 000 pop.  Average proximity network distance  **Proximity network distance** | 0.005 (0.008)  0.074 (0.061)  **0.090** (0.072) | > 0.05  > 0.05  > 0.05 |
|  |  | Major chain fast food franchises | Density 2.5 km Euclidean buffer/10 000 pop.  **Average proximity network distance**  Proximity network distance | 0.034 (0.021)  **0.048** (0.051)  0.037 (0.045) | > 0.05  > 0.05  > 0.05 |
| Thornton (2009)^2^ | Fast food purchase (weekly) | Fast food outlets | Count 3 km network buffer  Variety 3 km network buffer  **Proximity network distance** | 0.049 (-0.03, 0.13)  0.104 (-0.03, 0.24)  **-0.198** (-0.43, 0.03) | > 0.05  > 0.05  > 0.05 |
|  | Fast food purchase (monthly) |  | Count 3 km network buffer  **Variety 3 km network buffer**  Proximity network distance | 0.049 (-0.02, 0.10)  **0.122** (0.02, 0.22)  -0.105 (-0.27, 0.07) | > 0.05  ***< 0.05***  > 0.05 |

*Largest effect size per study in **bold**. Significant p-values in the unexpected direction in **bold** and expected direction in ***bold italics***. ^4^ Unstandardised β coef. (SE). ^2^ Unstandardised β coef. (95% CI).

**Additional file 4d. Summary of associations with diet quality by food outlet type*.**

| **Author (date)** | **Outcome** | **Food outlet** | **GIS Method** | **Effect size (precision)** | **p-value** |
| --- | --- | --- | --- | --- | --- |
| Layte (2011)^2^ | DASH score | Convenience stores | Proximity network distance  **Count 500 m network buffer**  Count 1 km network buffer  Count 2 km network buffer | ­-0.03 (-0.22, 0.15)  **0.30** (-0.05, 0.65)  0.19 (-0.10, 0.47)  0.03 (-0.29, 0.35) | > 0.05  > 0.05  > 0.05  > 0.05 |
|  |  | Supermarkets | Proximity network distance  Proximity Euclidean distance  Count 500 m network buffer  Count 1 km network buffer  **Count 2 km network buffer** | ­-0.45 (-0.76, -0.15)  ­-0.54 (not reported)  0.32 (-0.42, 1.05)  0.25 (-0.21, 0.71)  **0.59** (0.20, 0.97) | ***< 0.01***  ***< 0.01***  > 0.05  > 0.05  ***< 0.01*** |
| Minaker (2013)^4^ | HEI-C | Convenience stores and supermarkets | **Proximity network distance**  RFEI 1 km Euclidean buffer | **2.24** (1.96)  ­-0.09 (0.07) | > 0.05  > 0.05 |
|  |  | Restaurants | **Proximity network distance**  Count 1 km Euclidean buffer | **2.12** (1.81)  0.01 (0.04) | > 0.05  > 0.05 |
|  |  | Food stores | Count 1 km Euclidean buffer  **Diversity 1 km Euclidean buffer** | ­-0.03 (0.10)  **­-0.06** (0.27) | > 0.05  > 0.05 |

*Largest effect size per study in **bold.** Significant p-values in the unexpected direction in **bold** and expected direction in ***bold italics***. ^2^ Unstandardised β coef. (95% CI). ^4^ Unstandardised β coef. (SE).
